# Reusable and highly enantioselective water-soluble Ru(II)-*Amm*-Pheox catalyst for intramolecular cyclopropanation of diazo compounds

**DOI:** 10.3762/bjoc.15.31

**Published:** 2019-02-06

**Authors:** Hamada S A Mandour, Yoko Nakagawa, Masaya Tone, Hayato Inoue, Nansalmaa Otog, Ikuhide Fujisawa, Soda Chanthamath, Seiji Iwasa

**Affiliations:** 1 Department of Chemistry, Faculty of Science, Tanta University, Tanta 31527, Egypt; 2 Department of Environmental and Life Sciences, Toyohashi University of Technology, 1-1 Hibarigaoka, Tempaku-Cho, Toyohashi 441-8580, Japan

**Keywords:** asymmetric synthesis, carbene transfer, cyclopropanation, diazoester, intramolecular diazoamide, Ru(II)-Pheox, water-soluble catalyst, Weinreb amide

## Abstract

A reusable and highly enantioselective catalyst for the intramolecular cyclopropanation of various diazo ester and Weinreb amide derivatives was developed. The reactions catalyzed by a water-soluble Ru(II)-*Amm*-Pheox catalyst proceeded smoothly at room temperature, affording the corresponding bicyclic cyclopropane ring-fused lactones and lactams in high yields (up to 99%) with excellent enantioselectivities (up to 99% ee). After screening of various catalysts, the Ru(II)-*Amm*-Pheox complex having an ammonium group proved to be crucial for the intramolecular cyclopropanation reaction in a water/ether biphasic medium. The water-soluble catalyst could be reused at least six times with little loss in yield and enantioselectivity.

## Introduction

**Figure 1:**
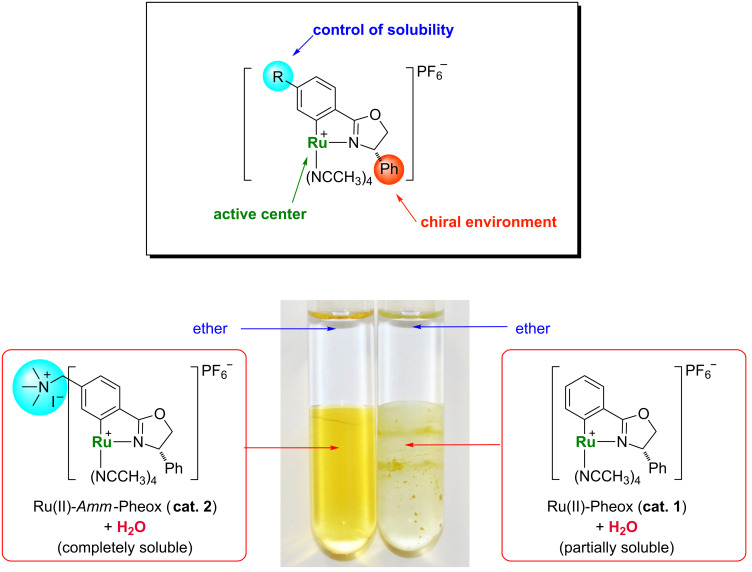
A comparison of the solubility of Ru(II)-Pheox (**cat. 1**) and Ru(II)-*Amm*-Pheox (**cat. 2**).

Water-soluble transition metal complexes have been attracting increasing interest for catalytic applications because of their many advantages such as simple product separation, low cost, safety, and environmentally friendly processing [1–13]. Thus, the study of organic reactions in water is an important area of research [14–21]. Nevertheless, only a few catalytic cyclopropanation reactions were carried out in aqueous media [22–28]. In our previous work, we developed Ru(II)-Pheox catalysts for the intramolecular cyclopropanation of *trans*-allylic diazoacetates in a biphasic medium [29–31]. During the course of our continuous study on the development of a series of Ru(II)-Pheox catalysts, we found the introduction of an ammonium group on the aromatic ring connected with Ru gave high solubility in the water phase compared to a normal Ru(II)-Pheox complex as shown below (Figure 1) [32–33].

Then, Ru(II)-*Amm*-Pheox (**cat. 2**) was found to be soluble in water but not in diethyl ether. This fact prompted us to explore the asymmetric intramolecular cyclopropanation of a variety of diazo compounds such as diazoacetates and diazoacetamides in a biphasic medium. Diazoacetates were tested in our catalytic system because they are widely used for intramolecular cyclopropanation reactions and also the resulted lactones are widely distributed in nature and have excellent biological activity, including strong antibiotic, antihelmetic, antifungal, antitumor, antiviral and anti-inflammatory, which make them interesting lead structures for new drugs [34]. That is why a great deal of attention has been paid to the synthesis of the lactone ring [35–36]. Diazoacetamides, in particular, diazo Weireb amides were tested in our catalytic system because the resulting cyclopropane products can be easily converted into the corresponding aldehydes, ketones, and alcohols [37–48].

## Results and Discussion

### Asymmetric cyclopropanation using various diazo compounds with Ru(II)-*Amm*-Pheox

Several diazoacetates and diazoacetamides were tested for asymmetric intramolecular cyclopropanation reactions using Ru(II)-*Amm*-Pheox catalyst (**cat. 2**) in H_2_O/ether biphasic medium as shown in Table 1. A diazo compound derived from allyl diazoacetate could be cyclopropanated affording the corresponding lactone with low yield and good enantioselectivity (Table 1, entry 1).

**Table 1:** Asymmetric intramolecular cyclopropanation using various substrates.^a^

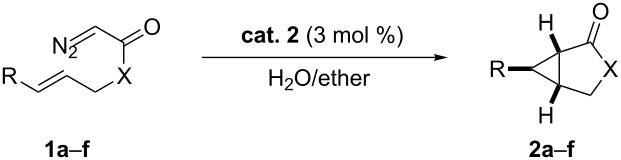				

entry	product		yield [%]^b^	ee [%]^c^

1	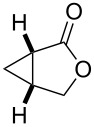	**2a**	18	88
2	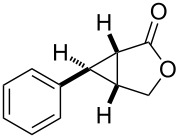	**2b**	93	93
3	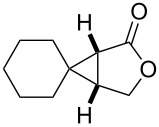	**2c**	56	97
4	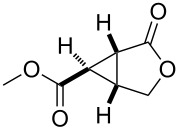	**2d**	38	96
5	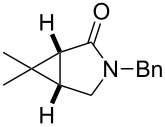	**2e**	50	43
6	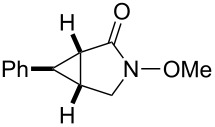	**2f**	99	99

^a^Reaction conditions: 3 mol % of the catalyst was dissolved in water (1 mL) and a solution of the diazo compound in ether (1 mL) was added. The reaction was stirred until the reaction was finished at room temperature. ^b^Isolated yield. ^c^Determined by chiral HPLC analysis.

In case of the diazo compound derived from cinnamyl diazoacetate the corresponding lactone was obtained in high yield with high enantioselectivity (Table 1, entry 2). A spirocyclopropanation product and a functionalized cyclopropane were obtained with high enantioselectivities (Table 1, entries 3 and 4). *N*-Benzyl-diazoacetamide underwent the asymmetric cyclopropanation reaction affording the corresponding lactam in moderate yield and moderate enantioselectivity (Table 1, entry 5). Interestingly, Ru(II)-*Amm*-Pheox complex (**cat. 2**) catalyzes the asymmetric intramolecular cyclopropanation of diazo Weinreb amide *(N*-cinnamyl-2-diazo-*N*-methoxyacetamide) in a water/ether mixture, giving the corresponding lactam in high yield with high enantioselectivity (99% yield, 99% ee, Table 1, entry 6). These results encouraged us to explore the asymmetric intramolecular cyclopropanation of diazo Weinreb amides in a biphasic medium.

### Asymmetric cyclopropanation using diazo Weinreb amide with Ru(II)-*Amm*-Pheox

Ru(II)-Pheox (**cat. 1**) was slightly soluble in water, and afforded only a 53% yield of product in 5 h (Table 2, entry 1). On the other hand, hydroxymethyl Ru(II)-Pheox (**cat. 3**), which is completely soluble in water, catalyzed the cyclization of **1f** affording chiral cyclopropylamide **2f** in 97% yield with a high enantioselectivity of 96% in 5 h (Table 2, entry 5) [49].

**Table 2:** Catalyst screening.^a^

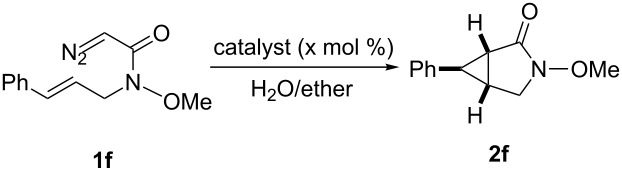				

entry	catalyst	X mol [%]	yield [%]^b^	ee [%]^c^

1	**cat. 1**	3	53	91
2	**cat. 2**	3	99	99
3	**cat. 2**	2	99	99
4	**cat. 2**	1	99	99
5	**cat. 3**	3	97	96
6	**cat. 4**	5	41	95
7	**cat. 5**	5	37	88
8	**cat. 6**	5	61	96

^a^Reaction conditions: (3 mol %) of the catalyst was dissolved in water (1 mL) and a solution of diazo compound **1f** in ether was added. The reaction was stirred until the reaction was finished at room temperature. ^b^Isolated yield. ^c^Determined by chiral HPLC analysis.
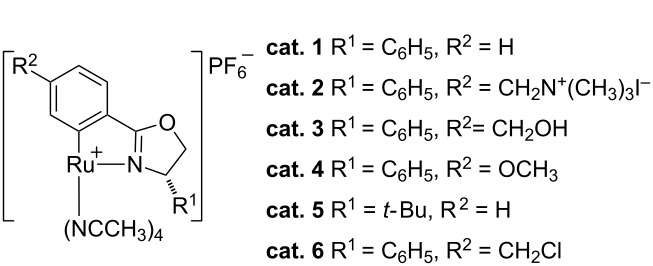

Next, the intramolecular cyclization of *trans*-allylic diazo Weinreb amide **1f** catalyzed by Ru(II)-*Amm*-Pheox (**cat. 2**) was examined in various solvents, as shown in Table S1 (Supporting Information File 1). Notably, the catalytic reaction proceeded in a variety of solvents, including aromatic, aliphatic, polar, non-polar, and halogenated solvents (Table S1, entries 1–8). In non-polar solvents (Table S1, entries 1−4), in which Ru(II)-*Amm*-Pheox (**cat. 2**) was poorly soluble, low yields and moderate enantioselectivities were obtained. On the other hand, the reaction in halogenated solvents proceeded in moderate yields with high enantioselectivities (Table S1, entries 5−7).

Among the solvents examined, water/diethyl ether was identified as the solvent system of choice because it afforded the desired product in the highest yield and enantioselectivity at room temperature.

**Scheme 1:**
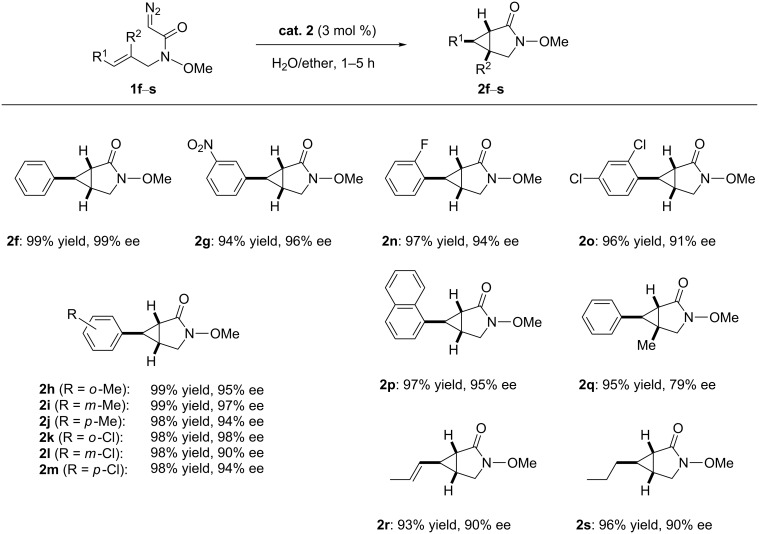
Intramolecular cyclopropanation of various *trans*-allylic diazo Weinreb amide derivatives catalyzed. Reaction conditions: to a solution of Ru(II)-*Amm*-Pheox (**cat. 2**, 3 mol %) in water (1 mL) was added a solution of *trans*-allylic diazo Weinreb amide derivatives **1f**–**s** and the reaction mixture was stirred at room temperature for 1–5 h.

**Scheme 2:**
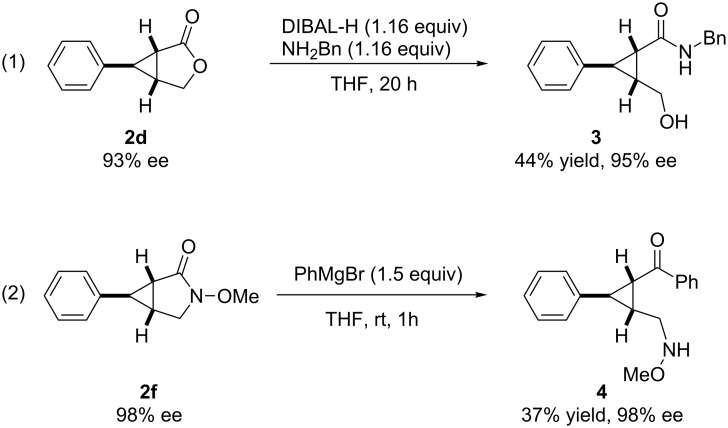
Synthetic transformation of cyclopropane products **2d** and **2f**.

With the optimal conditions in hand, various *trans*-allylic diazo Weinreb amide derivatives, prepared from the corresponding allylic alcohols by the Fukuyama method [50] were investigated as shown in Scheme 1. The catalytic system was applicable to a wide variety of substrates, which reacted smoothly to give the corresponding bicyclic products. For example, diazo Weinreb amide derivatives bearing electron-donating or electron-withdrawing groups at the *ortho*, *meta*, or *para* positions could be converted to the corresponding bicyclic products in excellent yields (up to 99%) and enantioselectivities (up to 98% ee, Scheme 1, **2g**−**m**).

A diazo compound with two chlorine substituents on the aromatic ring also underwent the cyclopropanation reaction affording the desired bicyclic compound in high yield and enantioselectivity (Scheme 1, **2o**). Remarkably, the derivative featuring the bulky naphthyl group instead of the phenyl group was also well tolerated and afforded the corresponding bicyclic product in excellent yield with high enantioselectivity (Scheme 1, **2p**).

Even the sterically demanding trisubstituted olefin-based allyl diazo Weinreb amide was found to be an effective substrate for the intramolecular cyclopropanation in this catalytic system, providing the corresponding product in high yield with moderate enantioselectivity (Scheme 1, **2q**). Similar results were obtained with *trans*-allylic diazo Weinreb amide derivatives bearing aliphatic substituents (Scheme 1, **2r** and **2s**). As shown in Figure S1 (Supporting Information File 1), the absolute configuration of product **2g** was determined to be (1*S*,5*R*,6*R*) by single-crystal X-ray diffraction analysis (Supporting Information File 1) [51]. Since the stereoselectivity of products depends on the *cis*/*trans* geometry of the reactants, other stereoisomers such as the (1*S*,5*R*,6*S*) product was not formed in this catalytic cyclopropanation. During the reaction process, no other stereoisomers were obtained.

Next, the reusability of water-soluble catalyst **cat. 2** was examined. After separation of the ether layer, the aqueous phase was extracted with diethyl ether several times until no product remained. A new solution of *trans*-allylic diazo Weinreb amide **1f** in ether was added, and the mixture was stirred until completion of the reaction. Interestingly, Ru(II)-*Amm*-Pheox catalyst (**cat. 2**) could be reused at least six times with little loss of reactivity and enantioselectivity (Table 3). It is expected that the decreasing activity depended on the catalyst leakage during the work-up.

**Table 3:** Reusability of Ru(II)-*Amm*-Pheox (**cat. 2**).^a^

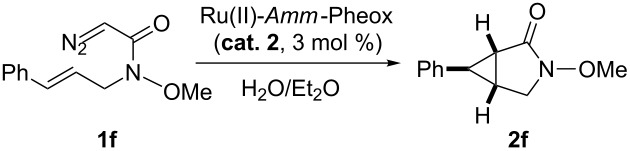				

entry	run	time [h]	yield [%]^b^	ee [%]^c^

1	1	50 min	99	99
2	2	2	97	99
3	3	16	95	97
4	4	24	94	95
5	5	24	96	92
6	6	36	93	90

^a^Reaction conditions: After the use of the catalyst in the first run, the ether layer was separated and the aqueous layer was washed 3 times with ether. A new amount of *trans*-allylic diazo Weinreb amide **1f** dissolved in Et_2_O (2.0 mL) was added and the reaction mixture was stirred until the end of the reaction. ^b^Isolated yield. ^c^Determined by chiral HPLC analysis.

### Synthetic transformations of **2d** and **2f**

In order to demonstrate the advantages of products, further synthetic transformations of the cyclopropane products were investigated. A ring opening and direct amidation of the chiral cyclopropane-fused γ-lacton **2d** using DIBAL-H with benzylamine afforded the desired product **3** in 44% yield with 95% ee (Scheme 2, reaction 1) [52]. We then investigated the arylation of chiral cyclopropylamide **2f** with Grignard reagent PhMgBr (Scheme 2, reaction 2). Only 37% of cyclopropyl ketone **4** were observed at room temperature with unaltered enantioselectivity (98% ee).

## Conclusion

In conclusion, we have developed an efficient water-soluble Ru(II)-*Amm*-Pheox (**cat. 2**) for the intramolecular cyclopropanation of *trans*-allylic diazo Weinreb amide derivatives. The water-soluble catalyst provided excellent yields of the bicyclic products (up to 99%) with excellent enantioselectivities (up to 99% ee). The easy separation of the ether layer containing the cyclopropane product allowed for simple reuse of the catalyst in the water phase for at least six times with little loss of reactivity and enantioselectivity.

## Supporting Information

File 1 Full experimental details and analytical data (reaction method, ^1^H NMR, ^13^C NMR, HPLC, X-ray analysis).
