# Supplementary material for: Reusable and highly enantioselective water-soluble Ru(II)-Amm-Pheox catalyst for intramolecular cyclopropanation of diazo compounds
Source: Beilstein J Org Chem. 2019 Feb 6;15:357–63. doi: 10.3762/bjoc.15.31 (PMC6369996; doi:10.3762/bjoc.15.31)
Supplement: File 1 — Full experimental details and analytical data (reaction method, 1H NMR, 13C NMR, HPLC, X-ray analysis). [file Beilstein_J_Org_Chem-15-357-s001.pdf]

BEILSTEIN JOURNAL OF ORGANIC CHEMISTRY

Supporting Information

for

Reusable and highly enantioselective water-soluble Ru(II)-*Amm*-Pheox catalyst for intramolecular cyclopropanation of diazo compounds

Hamada S. A. Mandour, Yoko Nakagawa, Masaya Tone, Hayato Inoue, Nansalmaa Otog, Ikuhide Fujisawa, Soda Chanthamath and Seiji Iwasa

Beilstein J. Org. Chem. **2019,** *15,* 357–363. doi:10.3762/bjoc.15.31

Full experimental details and analytical data (reaction method, ^{1}H NMR, ^{13}C NMR, HPLC, X-ray analysis)

License and Terms: This is a supporting information file under the terms of the Creative Commons Attribution License (http://creativecommons.org/licenses/by/4.0). Please note that the reuse, redistribution and reproduction in particular requires that the authors and source are credited.
The license is subject to the *Beilstein Journal of Organic Chemistry* terms and conditions: (https://www.beilstein-journals.org/bjoc)

1. Analytical data for diazo compounds (1a–1e).. S2
2. Typical procedure for the synthesis of trans-allylic diazo-Weinreb amide derivatives (1f–s)... S4
3. Analytical data for various *trans*-allylic diazo-Weinreb amide derivatives products (1f–s) S5
4. Typical procedures for intramolecular cyclopropanation reactions of various trans-allylic diazo-Weinreb amide derivatives using Ru(II)-Amm-Pheox as a catalyst (2a–s)... S6

5. Analytical data for various products (2a–s)... S6

6. Solubility of catalyst S13

7. Solvent screening (Table S1).. S14

8. Reaction mechanism (Figure S1) S15

9. X-ray analysis (Figure S2) S16

10. NMR spectral data S17

11. HPLC spectral data S53

12. References... S84

Materials and general methods

All reactions were performed under air atmosphere unless otherwise noted. Diethyl ether (Et_2O) was purchased from Kanto Chemical Co., Inc. All reactions were monitored by thin layer chromatography (TLC), glass plates pre-coated with silica gel Merck KGaA 60 F254, layer thickness 0.2 mm. All the starting materials are commercially available and were used after purification. The products were visualized by irradiation with UV light or by treatment with a solution of phosphomolybdic acid or by treatment with a solution of *p*-anisaldehyde. Flash column chromatography was performed using silica gel (Merck, Art. No.7734). ^{1}H NMR and ^{13}C NMR (500 MHz, 400 MHz) spectra were recorded on JEOL JNM-ECX500, JEOL JNM-ECS400 spectrometer. Chemical shifts are reported as δ values (ppm) relative to $CDCl_3$ (7.26 ppm). The following abbreviations were used to explain the multiplicities: s = singlet, d = doublet, t = triplet, q = quartet, m = multiplet and b = broad. Optical rotations were performed

with a JASCO P-1030 polarimeter at the sodium D line (1.0 mL sample cell). DART mass (positive mode) analyses were performed on a LC-TOF JMS-T100LP. Infrared (IR) spectra were recorded on an FT/IR-4600 instrument (JASCO Co., Ltd., Tokyo, Japan). Enantiomeric excesses were determined by high-performance liquid chromatography (HPLC) analyses with a JASCO GULLIVER using Daicel CHIRALPAK or CHIRALCEL columns.

1. Analytical data for various diazo compounds

Allyl 2-diazoacetate[1] (1a)

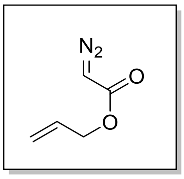

Yellow liquid; ^{1}H NMR (500 MHz, $CDCl_3$) δ 5.93 (ddt, *J* = 16.05, 10.32, 5.35 Hz, 1H), 5.32 (dd, *J* = 17.20, 1.53 Hz, 1H), 5.25 (dd, *J* = 10.32, 1.15 Hz, 1H), 4.77 (brs, 1H), 4.68–4.64 (m, 2H) ppm.

Cinnamyl 2-diazoacetate[2] (1b)

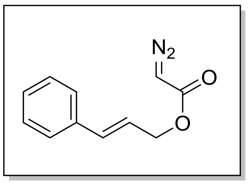

Yellow liquid; ^{1}H NMR (500 MHz, $CDCl_3$) δ 7.39 (d, *J* = 7.26 Hz, 2H), 7.32 (t, *J* = 7.45 Hz, 2H), 7.28–7.23 (m, 1H), 6.66 (d, *J* = 16.05 Hz, 1H), 6.28 (dt, *J* = 16.05, 6.50 Hz, 1H), 4.81 (dd, *J* = 6.50, 1.15 Hz, 2H), 4.78 (brs, 1H) ppm.

***N*-Benzyl-2-diazo-*N*-(3-methylbut-2-en-1-yl)acetamide[3] (1e)**

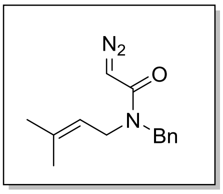

Yellow liquid; ^{1}H NMR (500 MHz, $CDCl_3$) δ 7.19–7.37 (m, 5H), 5.11 (brs, 1H), 4.93 (brs, 1H), 4.45 (brs, 2H), 3.78 (brs, 2H), 1.71 (s, 3H), 1.58 (s, 3H) ppm.

Methyl (*E*)-4-(2-diazoacetoxy)but-2-enoate[4] (1d)

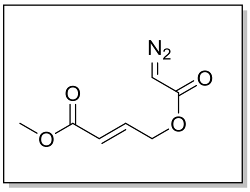

Yellow liquid; ^{1}H NMR (500 MHz, $CDCl_3$) δ 6.95 (dt, *J* = 16.05, 4.59 Hz, 1H), 6.02 (dt, *J* = 15.67, 1.91 Hz, 1H), 4.83 (dd, *J* = 4.59, 1.91 Hz, 2H), 4.83 (brs, 1H), 3.75 (s, 3H) ppm.

2-Cyclohexylideneethan-1-ol

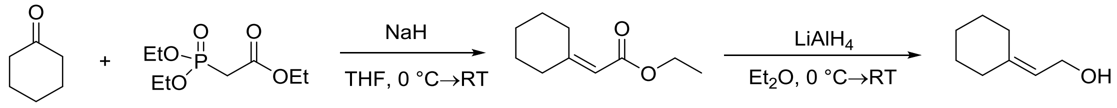

NaH in oil (132 mg, 5.5 mmol, 1.1 equiv) was washed by hexane, and THF was added (5 mL) at 0 °C. To a mixture was added triethyl phosphonoacetate (1.0 mL, 5 mmol, 1 equiv) and cyclohexanone in THF (5 mL). The mixture was stirred at 0 °C to rt for 8 h. The reaction mixture was quenched with NH_4Cl aq. (5 mL) and extracted with Et_2O. The organic layer was dried over Na_2SO_4, and concentrated under reduced pressure to give ethyl 2-cyclohexylideneacetate as a colorless oil (93% yield, 783.8 mg, 4.7 mmol). This product was used for next reaction without further purification.

A solution of $LiAlH_4$ (353.7 mg, 9.3 mmol, 2 equiv) in Et_2O (5 mL) was added a solution of reactant (783.8 mg, 4.7 mmol, 1 equiv) in Et_2O (5 mL) at 0 °C. The mixture was stirred at 0 °C to rt for 2 h. The reaction mixture was quenched with H_2O and extracted with CH_2Cl_2. Purification was performed by silica gel column chromatography (Hex/EA = 9/1) to give 2-cyclohexylideneethan-1-ol as a colorless oil (71% yield, 418.0 mg, 3.3 mmol). ^{1}H NMR (500 MHz, $CDCl_3$) δ 5.36 (t, *J* = 7.26 Hz, 1H), 4.14 (d, *J* = 7.26 Hz, 2H), 2.23–2.08 (m, 4H), 1.58–1.51 (m, 6H) ppm.

2-Cyclohexylideneethyl 2-diazoacetate (1c)

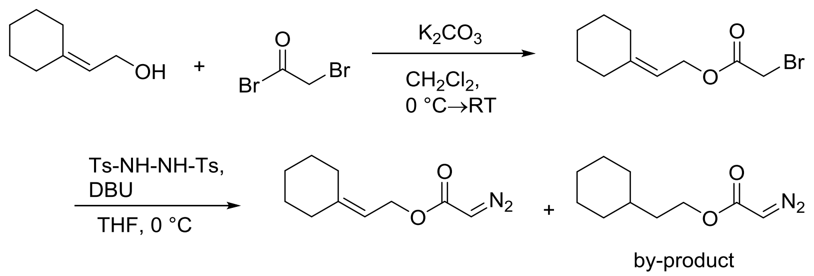

Bromoacetyl bromide (0.85 mL, 9.93 mmol, 3 equiv) was slowly added to a stirred solution of 2-cyclohexylideneethan-1-ol (418 mg, 3.31 mmol, 1 equiv) and K_2CO_3 in CH_2Cl_2 (15 mL). The mixture was stirred at 0 °C to rt for 1 h. The reaction mixture was quenched with H_2O and extracted with CH_2Cl_2. Purification was performed by silica gel column chromatography to give 2-cyclohexylideneethyl 2-bromoacetate (85% yield, 696.4 mg, 2.81 mmol).

To a solution of 2-cyclohexylideneethyl 2-bromoacetate (696.0 mg, 2.81 mmol, 1 equiv) and *N*,*N*'-ditosylhydrazine (1.4 g, 4.21 mmol, 1.5 equiv) were dissolved in THF (8.5 mL) and cooled down to 0 °C for 30 min. The reaction mixture was quenched with $NaHCO_3$ aq. and extracted with Et_2O, the organic layer was dried over $NaSO_4$ and evaporated to give the crude product. Purification was performed by column chromatography (Hex/EA = 50/1, Hex/CH_2Cl_2 = 4/1) to give 2-cyclohexylideneethyl 2-diazoacetate as a yellow oil (187.4 mg, 34% yield, 0.96 mmol). This yield was determined by ^{1}H NMR, because desired product was not purified completely by column chromatography. ^{1}H NMR (500 MHz, $CDCl_3$) δ 5.29 (t, *J* = 7.26 Hz, 1H), 4.74 (brs, 1H), 4.67 (d, *J* = 7.26 Hz, 2H), 2.09–2.23 (m, 4H), 1.50–1.58 (m, 6H) ppm. IR (neat) ν 2930, 2109, 1695 cm^{-1}. HRMS (DART) calcd for $C_{10}H_{15}N_2O_2$ $[M+H]^+$: 195.1133 found: 195.1132.

2. Typical procedure for the synthesis of *trans*-allylic diazo-Weinreb amide derivatives [6]

According to the Fukuyama method, *O*-methylhydroxylamine hydrochloride was added to a solution of *trans*-allylic bromide and K_2CO_3 in CH_3CN, the reaction mixture was stirred at room temperature until the reaction was completed. After the evaporation of the solvent, the residue was used for the next step without further purification. K_2CO_3 and bromoacetyl bromide were added to the residue at 0 °C. The reaction mixture was stirred for 15 min and then the reaction was quenched with H_2O and extracted with CH_2Cl_2. The organic layer was washed with brine and dried over Na_2SO_4. After the evaporation of the solvent, the residue was used for the next step without further purification. The resulting bromoacetate was dissolved in THF. *N*,*N*`-ditosylhydrazine and DBU were added dropwise at 0 °C and the reaction was stirred for 10 min. The reaction was quenched by the addition of saturated solution of $NaHCO_3$ and extracted with diethyl ether. The organic layer was washed with brine, dried over Na_2SO_4 and evaporated to give crude *trans*-allylic diazo-Weinreb amide derivatives that were purified by column chromatography to give the desired products.

3. Analytical data for various *trans*-allylic diazo-Weinreb amide derivatives products

Data for compounds 1f–h were already reported. [5]

(*E*)-2-Diazo-*N*-methoxy-*N*-[3-(3-nitrophenyl)allyl]acetamide (1g) [6]

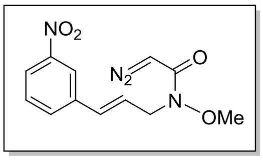

Yellow liquid; IR: ν = 2984, 1715 cm^{-1}. ^{1}H NMR (500 MHz, $CDCl_3$): δ = 3.70 (s, 3H), 4.39 (dd, *J* = 1.15 Hz, *J* = 6.31 Hz, 2H), 5.37 (s, 1H), 6.34–6.40 (m, 1H), 6.63 (d, *J* = 16.05 Hz, 1H), 7.47 (t, *J* = 16.05 Hz, 1H), 7.66 (d, *J* = 7.64 Hz, 1H), 8.07 (d, *J* = 8.03 Hz, 1H), 8.21 (s, 1H) ppm. ^{13}C NMR (125 MHz, $CDCl_3$): δ = 46.9, 48.7, 62.6, 121.2, 122.5, 127.0, 129.6, 131.4, 132.4, 138.3, 148.7, 168.8 ppm. HRMS (DART) m/z calcd for $C_{12}H_{14}N_4O_3$ $[M+H]^+$ 262.1066 found: 262.1062.

(*E*)-2-Diazo-*N*-[3-(2-fluorophenyl)allyl]-*N*-methoxyacetamide (1n) [6]

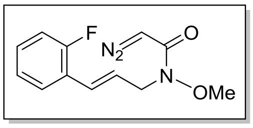

Yellow liquid; IR: ν = 2954, 1719 cm^{-1}. ^{1}H NMR (500 MHz, $CDCl_3$): δ = 3.68 (s, 3H), 4.37 (dd, *J* = 1.53, *J* = 6.05 Hz, 2H), 5.36 (s, 1H), 6.27–6.33 (m, 1H), 6.73 (d, *J* = 16.05 Hz, 1H), 6.99–7.03 (m, 1H), 7.07 (t, *J* = 15.29 Hz, 1H), 7.17–7.22 (m, 1H), 7.42 (t, *J* = 17.20 Hz, 1H) ppm. ^{13}C NMR (125 MHz, $CDCl_3$): δ = 46.8, 49.4, 62.7, 115.7, 115.9, 124.2, 126.3, 127.6, 129.2, 159.3, 161.3, 168.7 ppm. HRMS (DART) m/z calcd for $C_{12}H_{14}FN_3O$ $[M+H]^+$ 236.1199, found 236.1192.

Data for compound 1o were already reported. [5]

(*E*)-2-Diazo-*N*-methoxy-*N*-[3-(naphthalen-1-yl)allyl]acetamide (1p) [6]

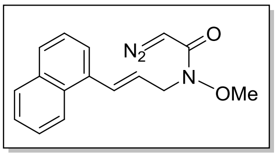

Yellow liquid; IR: ν = 2967, 1694 cm^{-1}. ^{1}H NMR (500 MHz, $CDCl_3$): δ = 4.84 (d, *J* = 6.5 Hz, 2H), 5.38 (s, 1H), 6.23–6.29 (m, 1H), 7.35 (d, *J* = 15.67 Hz, 1H), 7.43 (t, *J* = 15.29 Hz, 1H), 7.47–7.35 (m, 1H), 7.58 (d, *J* = 7.26 Hz, 1H), 7.77 (d, *J* = 8.41 Hz, 1H), 7.84 (d, *J* = 8.03 Hz, 1H), 8.1(d, *J* = 8.41 Hz, 1H) ppm. ^{13}C NMR (125 MHz, $CDCl_3$): δ = 46.8, 49.6, 62.9, 123.8, 124.1, 125.7, 125.9, 126.2, 126.8, 128.3, 128.6, 131.2, 131.2, 133.7, 134.3, 168.8 ppm. HRMS (DART) m/z calcd for $C_{16}H_{17}N_3O$ $[M+H]^+$ 268.1450, found 268.1453.

Data for compounds **1q–s** were already reported. [5]

4. Typical procedures for intramolecular cyclopropanation reactions of various *trans*-allylic diazo-Weinreb amide derivatives using Ru(II)-*Amm*-Pheox as a catalyst [6]

To a solution of Ru(II)-*Amm*-Pheox catalyst (0.003 mmol) in H_2O (1.00 mL), a solution of diazo Weinreb amide (1 mmol) in ether (1.00 mL) was added at room temperature. The progress of the reaction was monitored by TLC. After the reaction was completed, the two layers were separated and the aqueous layer was washed 3 times with ether. The collected ether layers were evaporated and the residue was purified using column chromatography on silica gel (Hex/EtOAc = 10:1) to give the desired product. The enantiomeric excesses of products were determined by HPLC analysis.

5. Analytical data for products

(1*S*,5*R*)-3-Oxabicyclo[3.1.0]hexan-2-one[1] (2a)

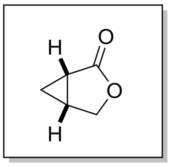

Colorless liquid; $[\alpha]^{26}_D$ = –54.4 (c = 0.2 , $CHCl_3$). ^{1}H NMR (500 MHz, $CDCl_3$) δ 4.36 (dd, *J* = 9.17, 4.97 Hz, 1H), 4.28 (brd, *J* = 9.17 Hz, 1H), 2.27–2.20 (m, 1H), 2.10–2.05 (m, 1H), 1.30–1.25 (m, 1H), 0.91–0.86 (m, 1H) ppm. The *ee* value was determined by chiral HPLC analysis. Column (CHIRALPAK IC-3), UV detector 220 nm, eluent: Hex/IPA = 7/3 , Flow late = 1.2 mL/min, tR = 18.5 min (major product), tR = 17.7 min (minor product).

(1*S*,5*R*,6*R*)-6-Phenyl-3-oxabicyclo[3.1.0]hexan-2-one[2] (2b)

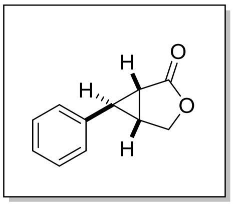

White Solid; $[\alpha]^{26}_D$ = –102.7 (c = 1.0, $CHCl_3$). ^{1}H NMR (500 MHz, $CDCl_3$) δ 7.31 (t, *J* = 7.64 Hz, 2H), 7.25 (t, *J* = 7.26 Hz, 1H), 7.06 (d, *J* = 6.88 Hz, 2H), 4.47 (dd, *J* = 9.56, 4.97 Hz, 1H), 4.41 (d, *J* = 9.56 Hz, 1H), 2.55–2.51 (m, 1H), 2.36–2.31 (m, 2H) ppm. The *ee* value was determined by chiral HPLC analysis. Column (CHIRALPAK OJ-H), UV detector 230 nm, eluent: Hex/IPA = 4/1, Flow late = 1.0 mL/min, tR = 20.4 min (major product), tR = 23.6 min (minor product).

(1*R*,5*S*)-3-Oxaspiro[bicyclo[3.1.0]hexane-6,1'-cyclohexan]-2-one (2c)

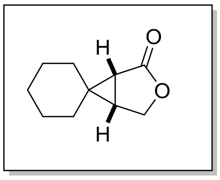

White solid; $[\alpha]^{26}_{D}$ = –43.4 (c = 0.7 , $CHCl_3$). ^1H NMR (500 MHz, $CDCl_3$) δ 4.36 (dd, *J* = 9.56, 4.78 Hz, 1H), 4.13 (d, *J* = 9.94 Hz, 1H), 2.05 (t, *J* = 5.35 Hz, 1H), 1.94 (d, *J* = 6.50 Hz, 1H), 1.64–1.41 (m, 8H), 1.40–1.29 (m, 2H) ppm. ^{13}C NMR (125 MHz, $CDCl_3$) 174.8, 66.2, 35.6, 30.2, 29.8, 29.3, 25.9, 25.2, 25.1, 24.9 ppm. The *ee* value was determined by chiral HPLC analysis. Column (CHIRALPAK ID), UV detector 230 nm, eluent: Hex/IPA = 20/1, Flow late = 1.0 mL/min, tR = 20.2 min (major product), tR = 24.8 min (minor product). IR (neat) ν 2920, 2849, 1770, 1176 cm^{-1}. HRMS (DART) calcd for $C_{10}H_{16}O_2$ $[M+H]^+$: 167.1072 found: 167.1074.

Methyl (1*R*,5*R*,6*R*)-2-oxo-3-oxabicyclo[3.1.0]hexane-6-carboxylate[4] (2d)

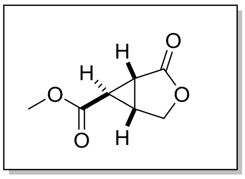

White solid; $[\alpha]^{28}_{D}$ = –52.4 (c = 0.3, $CHCl_3$). ^1H NMR (500 MHz, $CDCl_3$) δ 4.44 (dd, *J* = 9.94, 4.59 Hz, 1H), 4.32 (d, *J* = 9.94 Hz, 1H), 3.74 (s, 3H), 2.73–2.69 (m, 1H), 2.59 (dd, *J* = 6.50, 2.68 Hz, 1H), 2.00 (t, *J* = 3.06 Hz, 1H) ppm. The *ee* value was determined by chiral HPLC analysis. Column (CHIRALPAK IC), UV detector 220 nm, eluent: Hex/IPA = 7/3 (product was dissolved by CH_2Cl_2.), Flow late = 1.2 mL/min, tR = 83.8 min (major product), tR = 76.1 min (minor product).

(1*R*,5*S*)-3-Benzyl-6,6-dimethyl-3-azabicyclo[3.1.0]hexan-2-one[3] (2e)

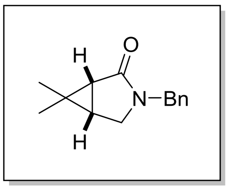

White solid; $[\alpha]^{28}_{D}$ = –25.8 (c = 1.0, $CHCl_3$). ^1H NMR (500 MHz, $CDCl_3$) δ7.22–7.34 (m, 5H), 4.50 (d, *J* = 14.52 Hz, 1H), 4.15 (d, *J* = 14.52 Hz, 1H), 3.37 (dd, *J* = 11.08, 6.88 Hz, 1H), 2.99 (d, *J* = 11.08 Hz, 1H), 1.83 (dd, *J* = 6.50, 1.91 Hz, 1H), 1.56 (t, *J* = 6.50 Hz, 1H), 1.08 (s, 3H), 0.94 (s, 3H) ppm. The *ee* value was determined by chiral HPLC analysis. Column (CHIRALPAK OJ-H), UV detector 254 nm, eluent: Hex/IPA = 30/1, Flow late = 1.0 mL/min, tR = 12.7 min (major product), tR = 15.6 min (minor product).

(1*S*,5*R*,6*R*)-3-Methoxy-6-phenyl-3-azabicyclo[3.1.0]hexane-2-one (2f)

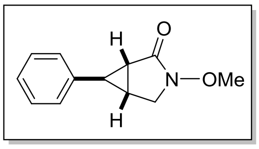

Yellow liquid; $[\alpha]_D^{28}$= −53.6 (c = 1.5, $CHCl_3$).

Spectral data of this compound were already reported.[5]

(1*S*,5*R*,6*R*)-6-(2-Methylphenyl)-3-methoxy-3-azabicyclo[3.1.0]hexane-2-one (2h)

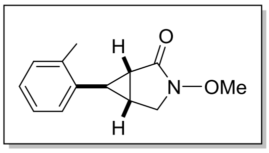

Yellow liquid; $[\alpha]_D^{27}$= −37.8 (c = 1, $CHCl_3$).

Spectral data of this compound were already reported.[5]

(1*S*,5*R*,6*R*)-6-(3-Methylphenyl)-3-methoxy-3-azabicyclo[3.1.0]hexane-2-one (2i)

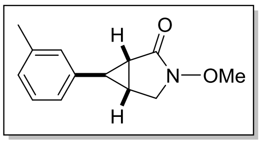

Yellow liquid; $[\alpha]_D^{27}$= −35.3 (c = 1, $CHCl_3$).

Spectral data of this compound were already reported.[5]

(1*S*,5*R*,6*R*)-6-(4-Methylphenyl)-3-methoxy-3-aza-bicyclo[3.1.0]hexane-2-one (2j)

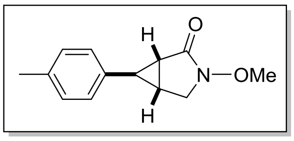

Yellow liquid; $[\alpha]_D^{25}$= −34.2 (c = 0.5, $CHCl_3$). Spectral data of this compound were already reported.[5]

(2-Chlorophenyl)-3-methoxy-3-azabicyclo[3.1.0]hexane-2-one (2k)

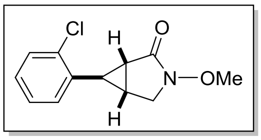

Yellow liquid; $[\alpha]_D^{26}$= −54.9 (c = 1, $CHCl_3$).

Spectral data of this compound were already reported.[5]

(3-Chlorophenyl)-3-methoxy-3-azabicyclo[3.1.0]hexan-2-one (2l)

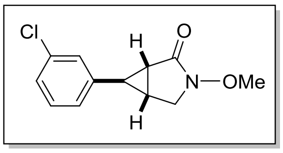

Yellow liquid; $[\alpha]_D^{28}$= −43.8 (c = 1, $CHCl_3$).

Spectral data of this compound were already reported.[5]

(1*S*,5*R*,6*R*)-6-(4-Chlorophenyl)-3-methoxy-3-azabicyclo[3.1.0] hexane-2-one (2m)

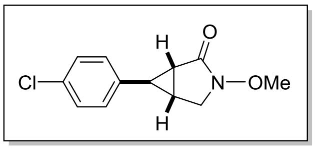

Yellow liquid; $[\alpha]_D^{25}$= −57.0 (c = 0.64, $CHCl_3$).

Spectral data of this compound were already reported.[5]

(1*S*,5*R*,6*R*)-3-Methoxy-6-(3-nitrophenyl)-3 azabicyclo[3.1.0] hexan-2-one (2g)[6]

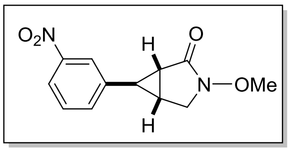

White solid; IR: ν = 2937, 1719 cm-1. $[\alpha]_D^{25}$= −41.2 (c = 1, $CHCl_3$). ^{1}H NMR (500 MHz, $CDCl_3$): δ = 2.17−2.21 (m, 2H), 2.28 (t, *J* = 6.50 Hz, 1H), 3.69 (d, *J* = 9.56 Hz, 1H), 3.77 (s, 3H), 3.89−3.93 (m, 1H) , 7.40 (d, *J* = 7.64 Hz, 1H), 7.64 (t, *J* = 15.67 Hz, 1H), 7.89 (s, 1H), 8.07 (d, 8.03) ppm. ^{13}C NMR (125 MHz, $CDCl_3$): δ = 19.3, 27.4, 29.4, 47.7, 62.4, 120.8, 122.0, 129.7, 132.5, 140.5, 148.5, 168.3 ppm. HRMS (DART) m/z calcd for $C_{12}H_{12}N_2O_4$ $[M+H]^+$ 284.0797, found 284.0796.

(1*S*,5*R*)-6-(2-Fluorophenyl)-3-methoxy-3-azabicyclo[3.1.0]hexan-2-one (2n)[6]

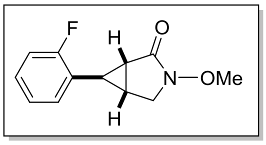

Yellow liquid; IR: ν = 2983, 1711 cm^{-1}. $[\alpha]_D^{25}$= −53.0 (c = 0.8, $CHCl_3$). ^{1}H NMR (500 MHz, $CDCl_3$): δ = 2.09−2.13 (m, 1H), 2.19−2.21 (m, 1H), 2.32 (t, *J* = 6.88 Hz, 1H), 3.69 (dd, *J* = 1.15 Hz, *J* = 9.75 Hz, 1H), 3.84−3.87 (m, 1H) , 6.89 (t, *J* = 15.29 Hz, 1H), 7.00−7.06 (m, 2H), 7.16−7.21 (m,1H) ppm. ^{13}C NMR (125 MHz, $CDCl_3$): δ = 18.2, 24.0, 25.4, 47.8, 62.3, 124.3, 125.3, 126.8, 128.5, 169.1 ppm. HRMS (DART) m/z calcd for $C_{12}H_{12}FNO_2$ $[M+H]^+$ 222.0930, found 222.0938.

(1*S*,5*R*,6*R*)-6-(2,4-dichlorophenyl)-3-methoxy-3-azabicyclo[3.1.0]hexan-2-one (2o)

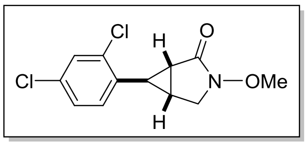

Yellow liquid; $[\alpha]_D^{27}$= −25.3 (c = 1, $CHCl_3$).

Spectral data of this compound were already reported.[5]

(1*S*,5*R*)-3-Methoxy-6-(naphthalen-1-yl)-3-azabicyclo[3.1.0]hexan-2-one (2p)[6]

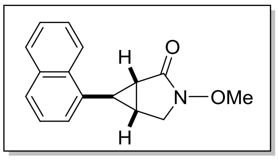

Yellow liquid; IR: ν = 2926, 1689 cm^{-1}. $[\alpha]_D^{26}$= −48 (c = 1, $CHCl_3$). ^{1}H NMR (500 MHz, $CDCl_3$) δ = 2.18−2.21 (m, 1H), 2.24−2.27 (m, 1H), 2.63 (t, *J* = 6.88 Hz, 1H), 3.82 (s, 3H), 3.84 (d, *J* = 1.15 Hz, 1H), 3.95−3.98 (m, 1H), 7.27 (d, *J* = 7.26 Hz, 1H), 7.38 (t, *J* = 15.67 Hz, 1H), 7.52 (t, *J* = 14.91 Hz, 1H), 7.58 (t, *J* = 15.29 Hz, 1H), 7.77 (d, *J* = 8.03 Hz, 1H), 7.86 (d, *J* = 7.64, 1H), 8.24 (d, *J* = 8.41 Hz, 1H). ^{13}C NMR (125 MHz, $CDCl_3$): δ = 17.0, 24.9, 28.3, 47.8, 62.3, 123.9, 124.5, 125.3, 126.2, 126.7, 128.2, 128.8, 132. 8, 133.2, 133.7, 169.6 ppm. HRMS (DART) m/z calcd for $C_{16}H_{15}NO_2$ $[M+H]^+$ 253.1103, found 253.1107.

(1*S*,5*R*,6*R*)-3-Methoxy-1-methyl-6-phenyl-3-aza-bicyclo[3.1.0]hexane-2-one (2q)

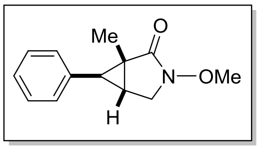

Yellow liquid; $[\alpha]_D^{28}$= −19.5 (c = 0.94, $CHCl_3$).

Spectral data of this compound were already reported.[5]

(1*S*,5*R*,6*R*)-3-Methoxy-6-((E)-prop-1-en-1-yl)-3-azabicyclo[3.1.0]hexan-2-one (2r)

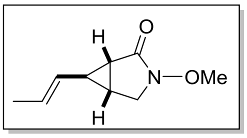

Yellow liquid; $[\alpha]_D^{26}$= −27.6 (c = 0.82, $CHCl_3$).

Spectral data of this compound were already reported.[5]

(1*S*,5*R*,6*R*)-3-Methoxy-6-propyl-3-azabicyclo[3.1.0] hexan-2-one (2s)

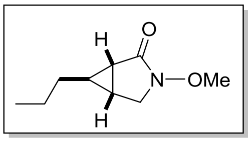

Yellow liquid; $[\alpha]_D^{26}$= −32.3 (c = 1, $CHCl_3$).

Spectral data of this compound were already reported.[5]

(1*S*,2*R*,3*R*)-*N*-Benzyl-2-(hydroxymethyl)-3-phenylcyclopropane-1-carboxamide (3)

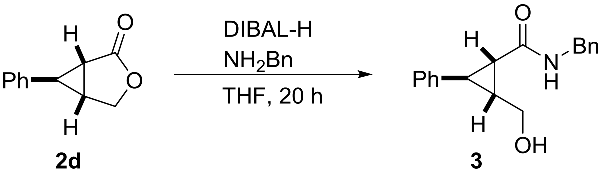

Benzylamine (23 µL, 0.218 mmol, 0.218 mmol, 1.16 equiv) was dissolved in dry THF (0.5 mL), cooled to –15 °C, and DIBAL-H (200 µL, 0.218 mmol, 1.16 equiv) was injected. After the mixture was stirred for 20 min, the solution was allowed to warm to 30 °C and left to react for 3 h. Then, it was cooled at –5 °C, and a solution of **2d** (32.7 mg, 0.188 mmol, 1 equiv) in THF (1 mL) was added with stirring for 10 min. After 20 h at room temperature, the reaction was quenched with water and 4N HCl, and the product was extracted three times with Et_2O. The combined organic phase was washed with $NaHCO_3$ aq., then dried Na_2SO_4, filtered and concentrated. The residue was purified by flash column chromatography with Hexane/EtOAc to give product **3** as white solid (44% yield, 95% ee, 16.0 mg, 0.056 mmol). $[\alpha]^{23}_{D}$ = –104.5 (c 0.8, $CHCl_3$). ^{1}H NMR (500 MHz, $CDCl_3$) δ 7.33 (dd, *J* = 7.64, 7.26 Hz, 2H), 7.29–7.23 (m, 5H), 7.19 (t, *J* = 7.26 Hz, 1H), 7.09 (d, *J* = 6.88 Hz, 2H), 6.20 (s, 1H), 4.49 (dd, *J* = 14.52, 5.73 Hz, 1H), 4.43 (dd, *J* = 14.52, 5.73 Hz, 1H), 4.12 (dd, *J* = 12.13, 3.63 Hz, 1H), 3.95 (dd, *J* = 12.13, 7.26 Hz, 1H), 2.90 (s, 1H), 2.79 (t, *J* = 5.73 Hz, 1H), 1.98–1.90 (m, 1H), 1.79 (dd, *J* = 8.79, 4.97 Hz, 1H) ppm. ^{13}C NMR (125MHz, $CDCl_3$) δ 171.72, 140.15, 137.91, 128.93, 128.68, 127.94, 127.82, 126.66, 126.26, 59.79, 44.18, 32.01, 31.09, 28.26 ppm. The ee value was determined by chiral HPLC analysis. Column (CHIRALPAK IC-3), UV detector 254 nm, eluent: Hex/IPA = 5/1, flow late = 1.0 mL/min, tR = 11.3 min (minor product), tR = 13.4 min (major product). IR (neat) ν 3307, 3063, 3029, 1639, 1544, 1028 cm^{-1}. HRMS (DART) calcd for $C_{18}H_{20}N_1O_2$ $[M+H]^+$: 282.1493 found: 282.1494.

((1*S*,2*R*,3*R*)-2-(Methoxyamino)methyl)-3-(*p*-tolyl)cyclopropyl)(phenyl)methanone (4) [6]

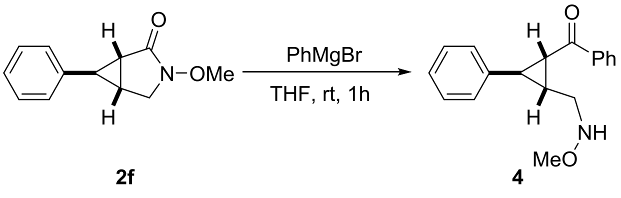

To a solution of **2f** (85.0 mg, 0.42 mmol) in dry THF (2.0 mL) was slowly added a solution of PhMgBr (0.28 mL, 1.5 equiv, 3 M in THF) at 0 °C. The resulting mixture

was stirred for 30 min at 0 ˚C followed by addition of H_2O (2.0 mL). The product was extracted with CH_2Cl_2 (10 mL). The organic phase was separated and dried with Na_2SO_4. The solvent was removed and the residue was purified by column chromatography on silica gel eluted with Hex/EA = 5/1 to give the desired cyclopropane **4** as a sole stereoisomer (37% yield, 98% ee, 43.8 mg, 0.16 mmol). $[\alpha]^{22}_{D}$ = –89.8 (c 1.2, $CHCl_3$). ^1H NMR (500 MHz, $CDCl_3$) δ 8.01 (d, *J* = 7.26 Hz, 2H), 7.54 (dd, *J* = 7.64, 7.26 Hz, 1H), 7.45 (dd, *J* = 8.03, 7.26 Hz, 2H), 7.29 (dd, *J* = 7.64, 7.26 Hz), 7.22–7.16 (m, 3H), 3.36 (s, 3H), 3.34 (dd, *J* = 13.76, 5.35 Hz, 1H), 3.18 (dd, *J* = 13.76, 8.41 Hz, 1H), 3.06 (dd, *J* = 9.17, 5.35 Hz, 1H), 2.98–2.89 (m, 1H), 2.35–2.30 (m, 1H) ppm. ^{13}C NMR (125MHz, $CDCl_3$) 197.26, 140.18, 138.42, 132.92, 128.64, 128.60, 128.23, 126.65, 126.52, 61.71, 48.31, 33.34, 32.25, 31.15 ppm. The ee value was determined by chiral HPLC analysis. Column (CHIRALPAK IC-3), UV detector 254 nm, eluent: Hex/IPA = 9/1, Flow late = 0.5 mL/min, tR = 28.8 min (minor product), tR = 31.4 min (major product). IR (neat) v 3030, 2934, 1666 cm^{-1}. HRMS (DART) calcd for $C_{18}H_{20}N_1O_2$ $[M+H]^+$: 282.1494 found: 282.1494.

6. Solubility of catalyst

- Ru(II)-Pheox

Ru(II)-Pheox (2.5 mg) was dissolved in H_2O (6 mL) and Et_2O (6 mL). Ru(II)-Pheox was partially dissolved in water phase. The Et_2O phase was separated, and no catalyst was observed after removal of the solvent. The water phase was filtered to remove insoluble solid and the water was removed under reduced pressure to give Ru(II)-Pheox (0.3 mg) in 12% recovered yield.

- Ru(II)-*Amm*-Pheox

Ru(II)-*Amm*-Pheox (2.5 mg) was dissolved in H_2O (6 mL) and Et_2O (6 mL). Ru(II)-*Amm*-Pheox almost completely dissolved in water phase. The Et_2O phase was separated, and no catalyst was observed after removal of the solvent. The water phase was filtered and the water was removed under reduced pressure to give Ru(II)-*Amm*-Pheox (1.9 mg) in 76% recovered yield.

Based on these results, we expected that the Ru(II)-*Amm*-Pheox has more high solubility in water than Ru(II)-Pheox.

7. Solvent screening

Table S1. Solvent screening[a]

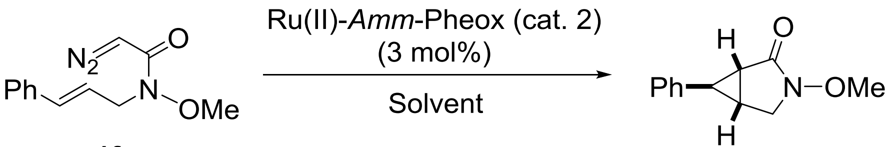

Entry	cat.	Solvent	Time [h]	Yield [%][b]	ee [%][c]
1	cat. 1	CH_2Cl_2	1 min	92	90
2	cat. 2	CH_2Cl_2	1 min	96	94
3	cat. 2	H_2O/Benzene	24	31	75
4	cat. 2	H_2O/Toluene	24	47	65
5	cat. 2	H_2O/Cyclohexane	24	37	67
6	cat. 2	H_2O/Heptane	24	42	75
7	cat. 2	$H_2O/CHCl_3$	10	76	88
8	cat. 2	H_2O/CH_2Cl_2	4	81	89
9	cat. 2	H_2O/ DCE	4	69	93
10	cat. 2	H_2O/DIPE	3	97	85
11	cat. 2	H_2O/Et_2O	1	99	99

[a]Reaction conditions: (3 mol %) of the cat. 2 was dissolved in water (1 mL) and a solution of diazo compound **1f** in the selected organic solvent (1 mL) was added. The reaction was stirred for the mentioned time at room temperature. [b]Isolated yield. [c]Determined by chiral HPLC analysis. DCE = 1,2-dichloroethane, DIPE = diisopropyl ether, Et_2O = diethyl ether.

8. Reaction mechanism

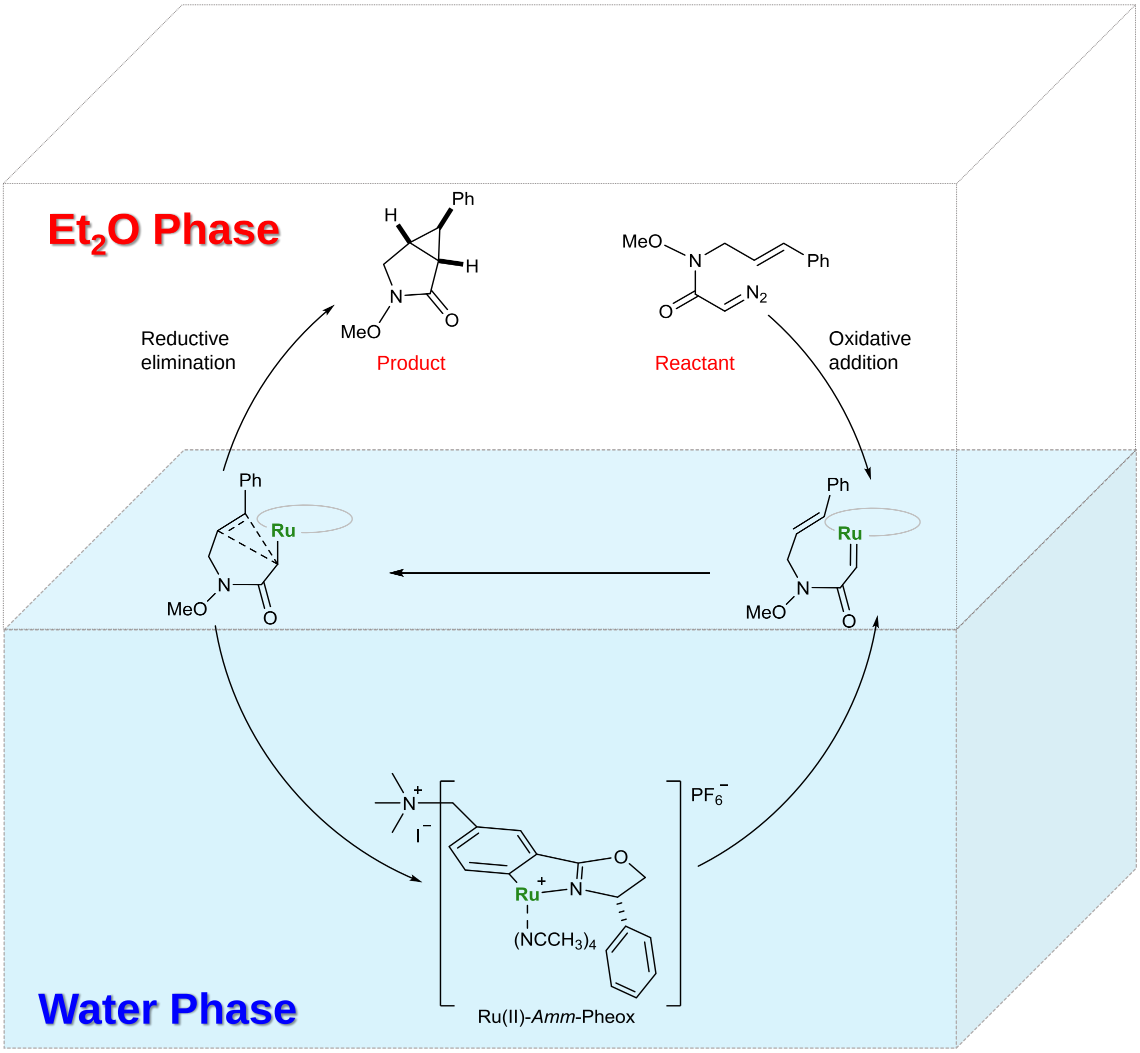

Figure S1. Plausible reaction mechanism in water/Et_2O.

9. X-ray analysis

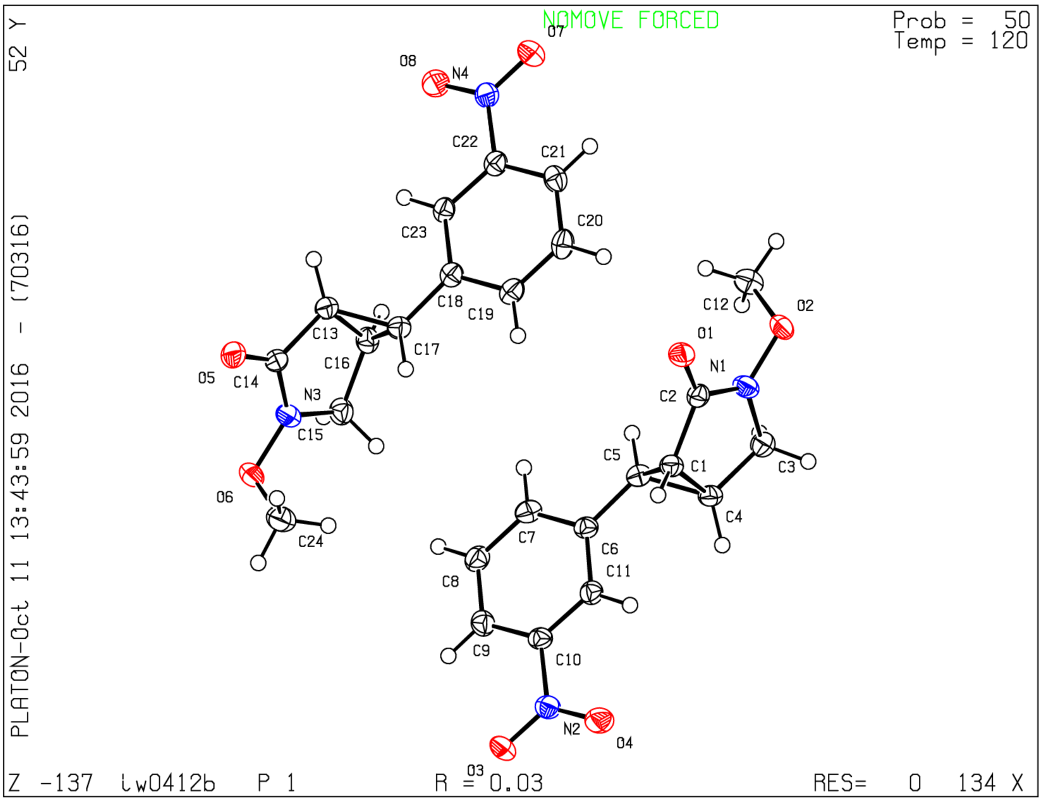

Figure S2. Thermal ellipsoid plot for 2g structure

10. NMR spectral data

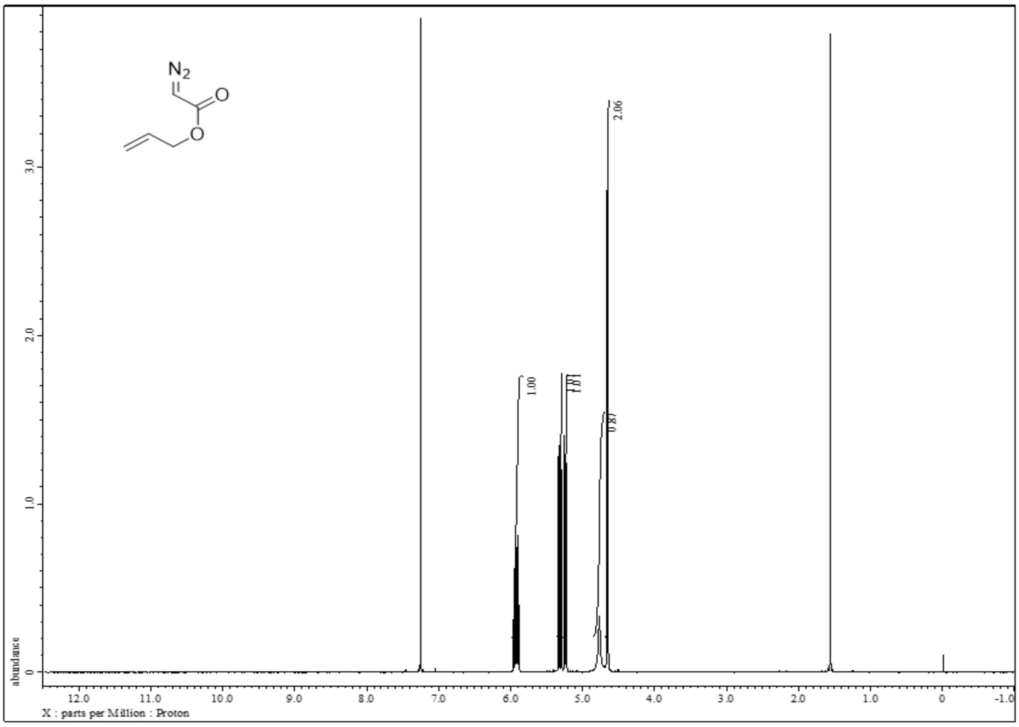

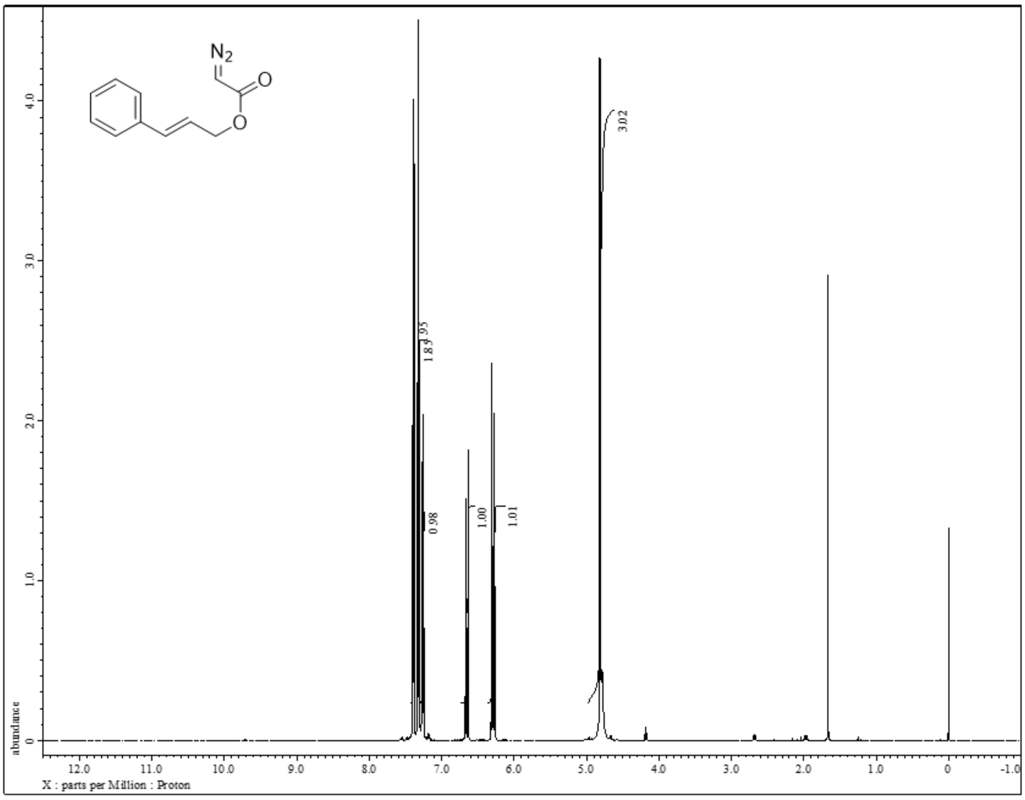

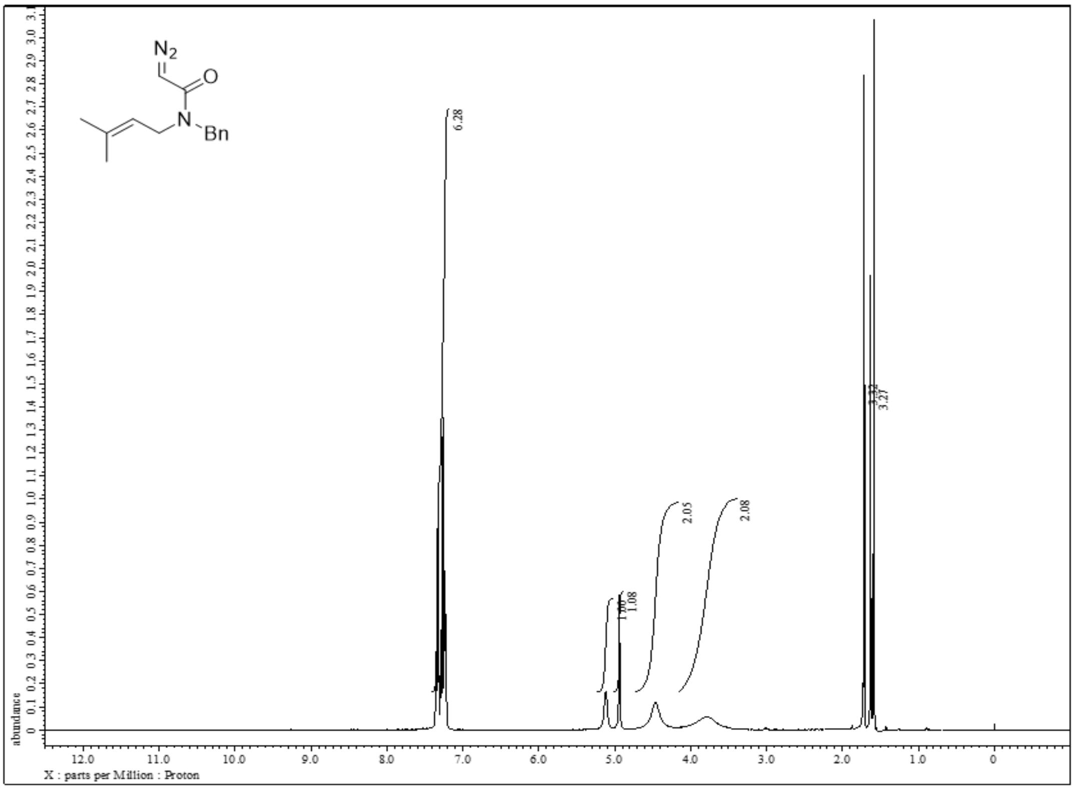
N2
O
N
Bn
6.28
1.00
1.08
2.05
2.08
3.32
3.27
abundance
12.0
11.0
10.0
9.0
8.0
7.0
6.0
5.0
4.0
3.0
2.0
1.0
0
X : parts per Million : Proton

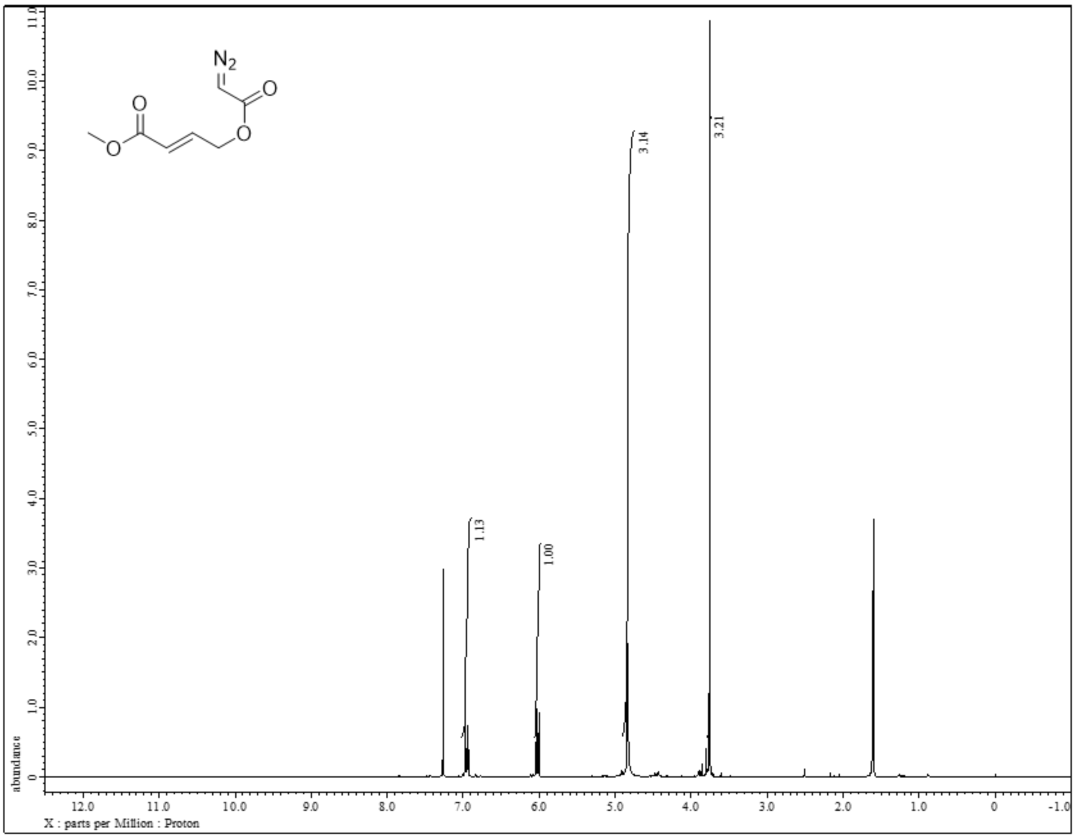
N2
O
O
O
O
1.13
1.00
3.14
3.21
abundance
12.0
11.0
10.0
9.0
8.0
7.0
6.0
5.0
4.0
3.0
2.0
1.0
0
-1.0
X : parts per Million : Proton

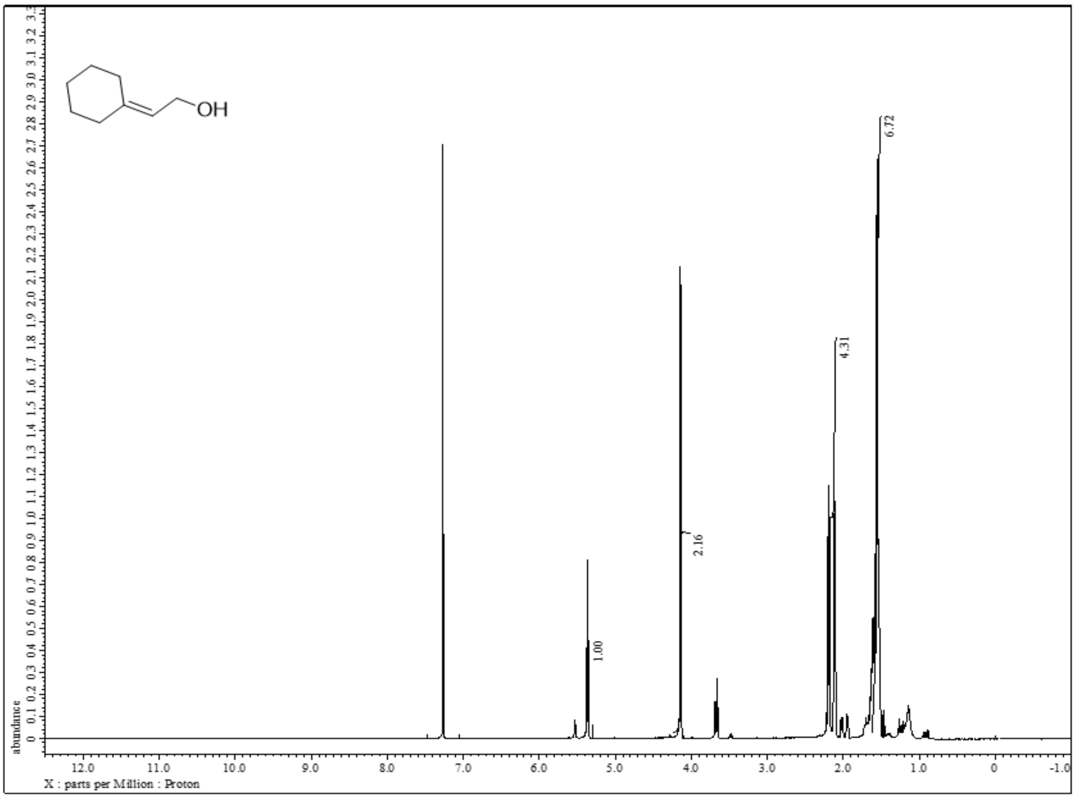

OH
abundance
X : parts per Million : Proton
1.00
2.16
4.31
6.72

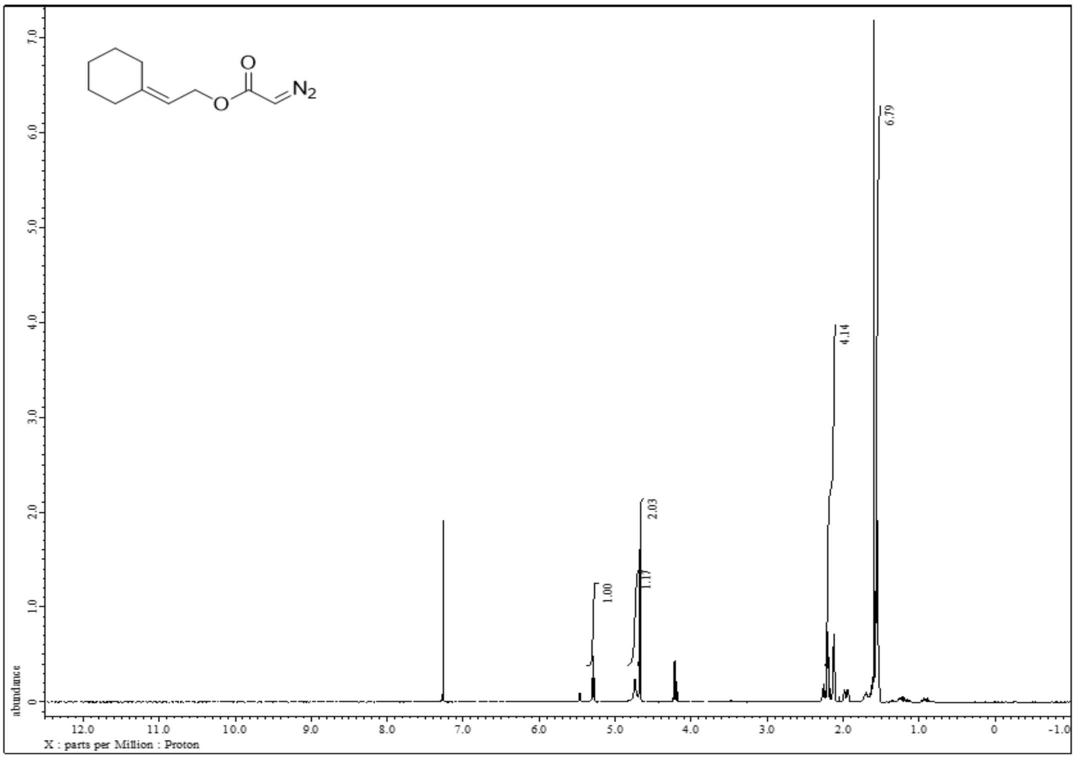

O
N2
abundance
X : parts per Million : Proton
1.00
1.17
2.03
4.14
6.79

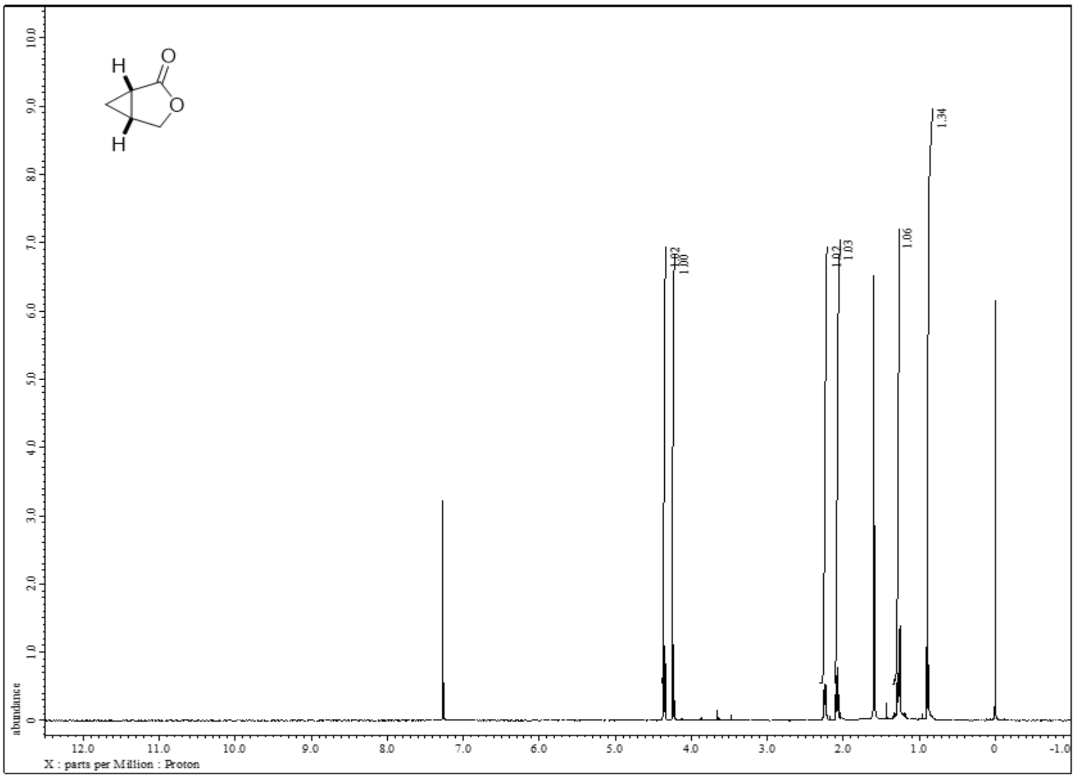
abundance
X : parts per Million : Proton

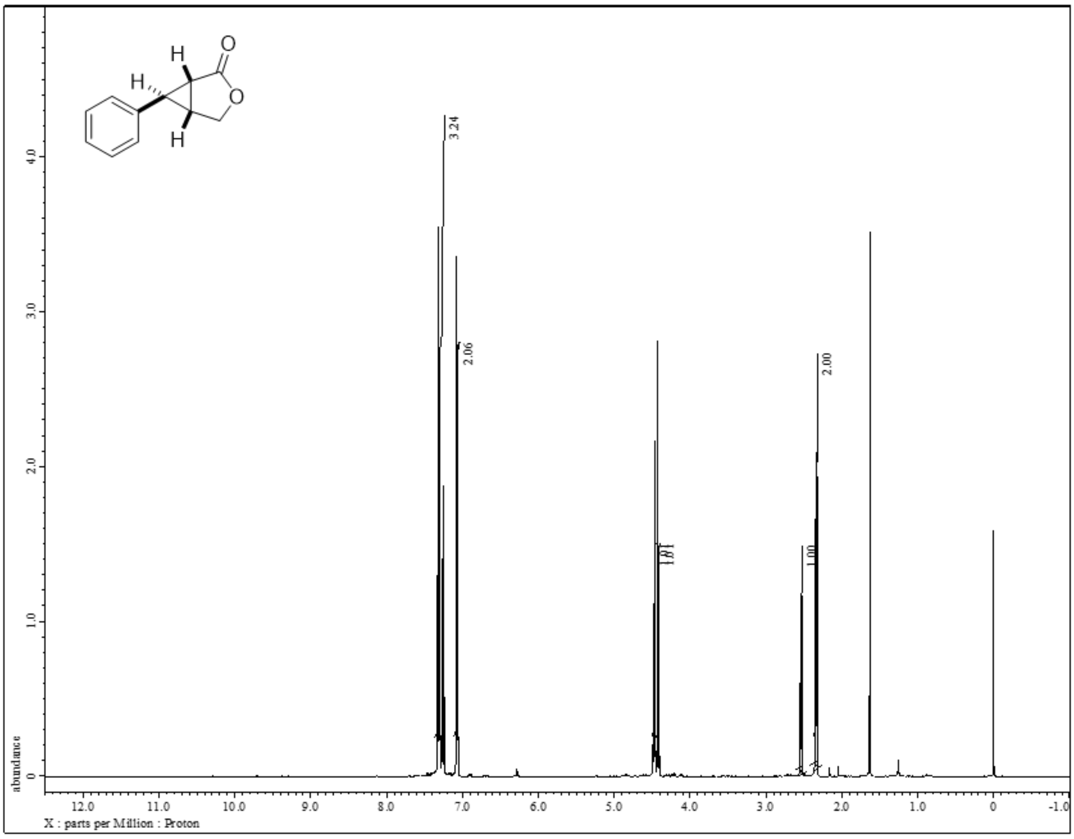
abundance
X : parts per Million : Proton

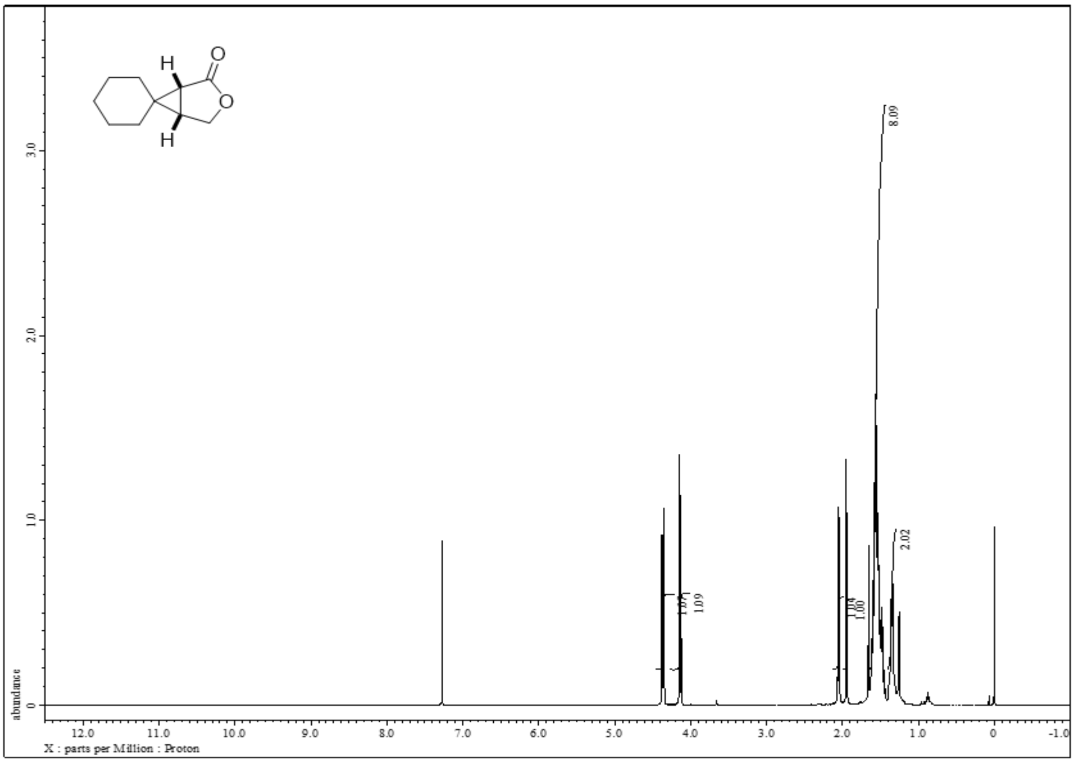

8.09
2.02
1.09
1.04
1.00
abundance
X : parts per Million : Proton

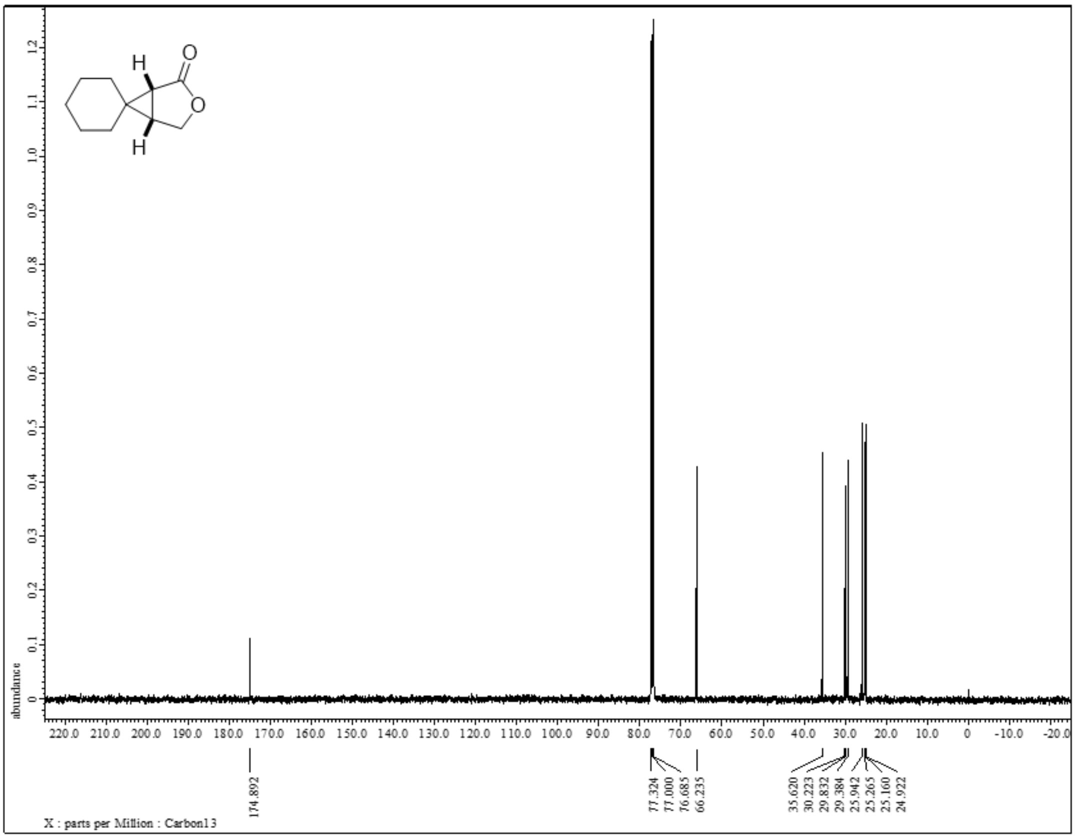

abundance
174.892
77.324
77.000
76.685
66.235
35.620
30.223
29.832
29.384
25.942
25.265
25.160
24.922
X : parts per Million : Carbon13

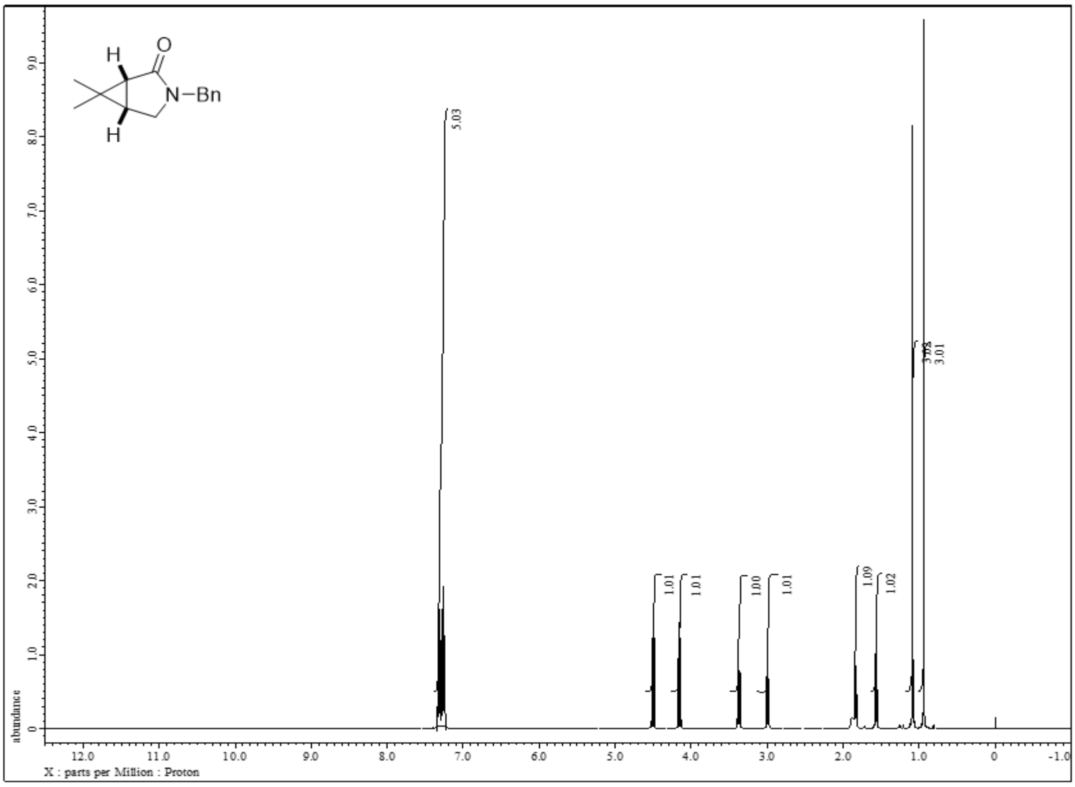

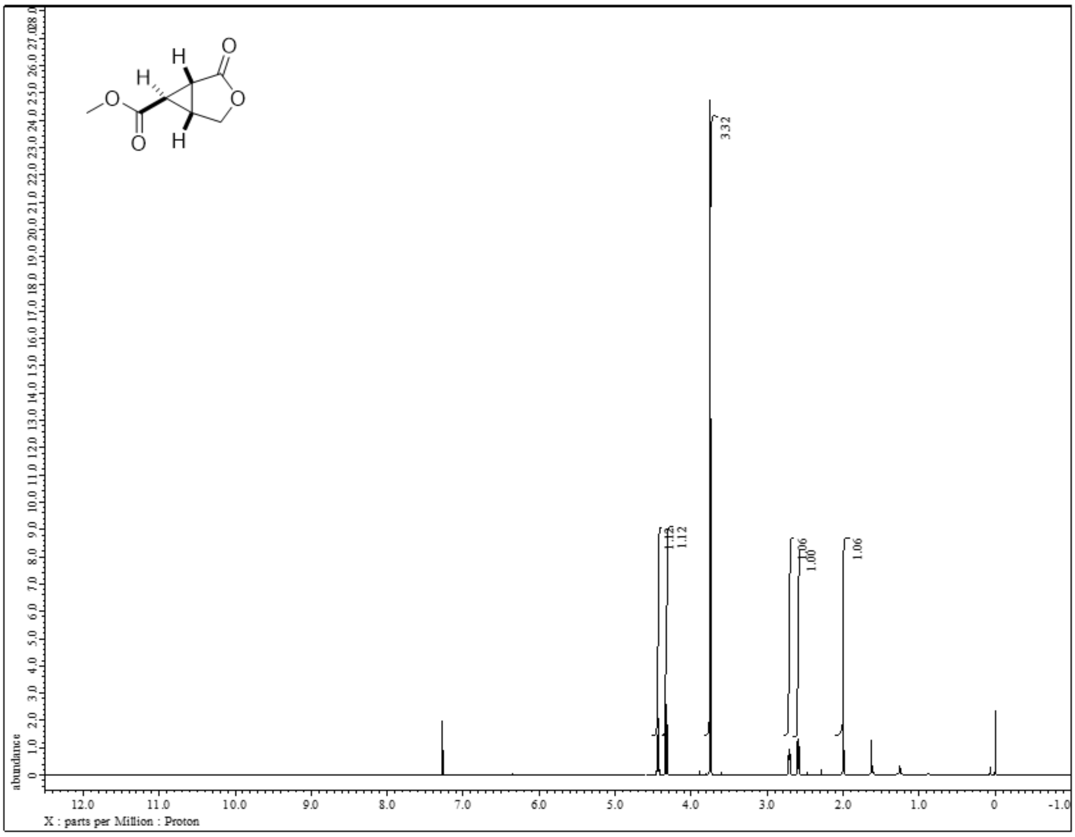

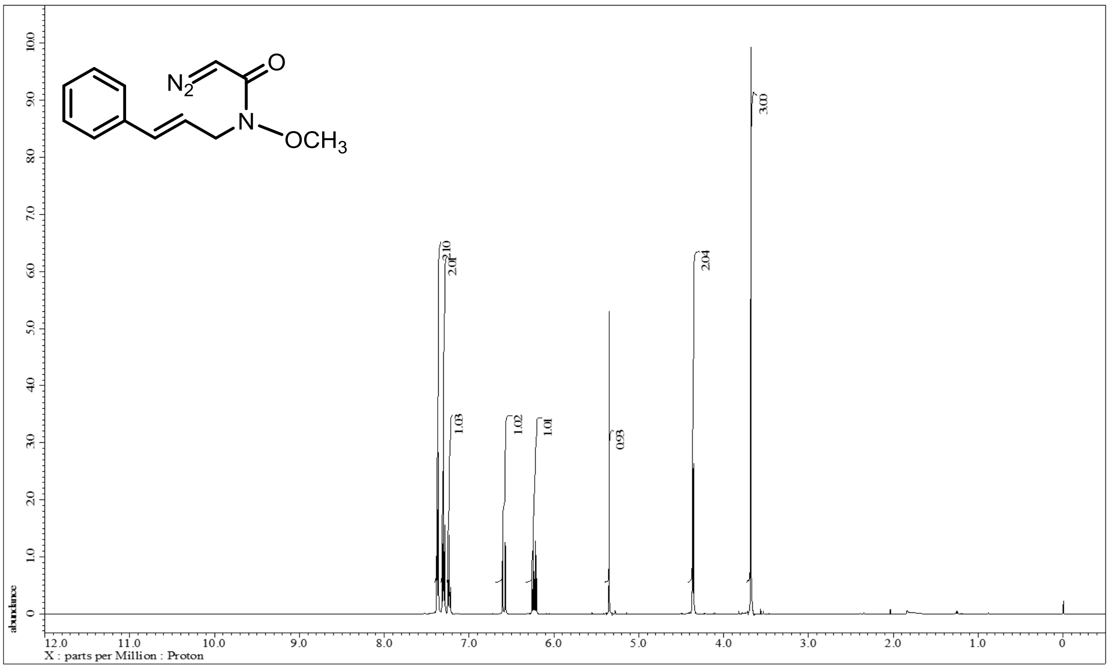
N2
O
N
OCH3
abundance
X : parts per Million : Proton
3.00
2.04
0.93
1.01
1.02
1.03
2.01
2.10

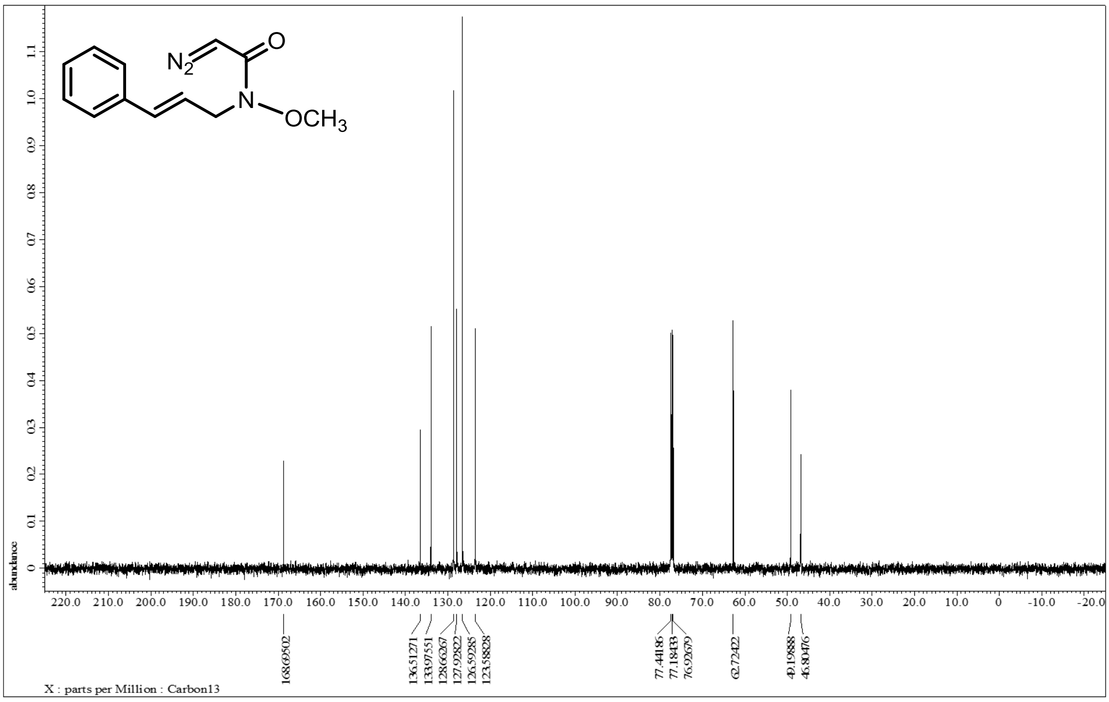
N2
O
N
OCH3
abundance
X : parts per Million : Carbon13
168.69502
136.51271
133.97551
128.66267
127.92822
126.59285
123.58828
77.44186
77.18433
76.92679
62.72422
49.19888
46.80476

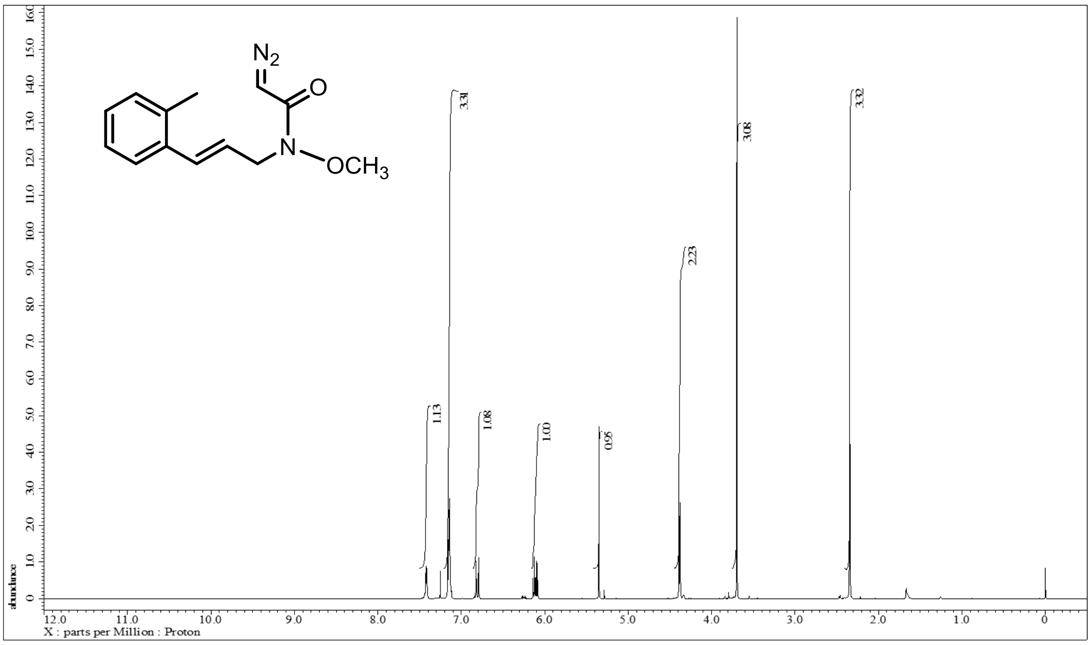

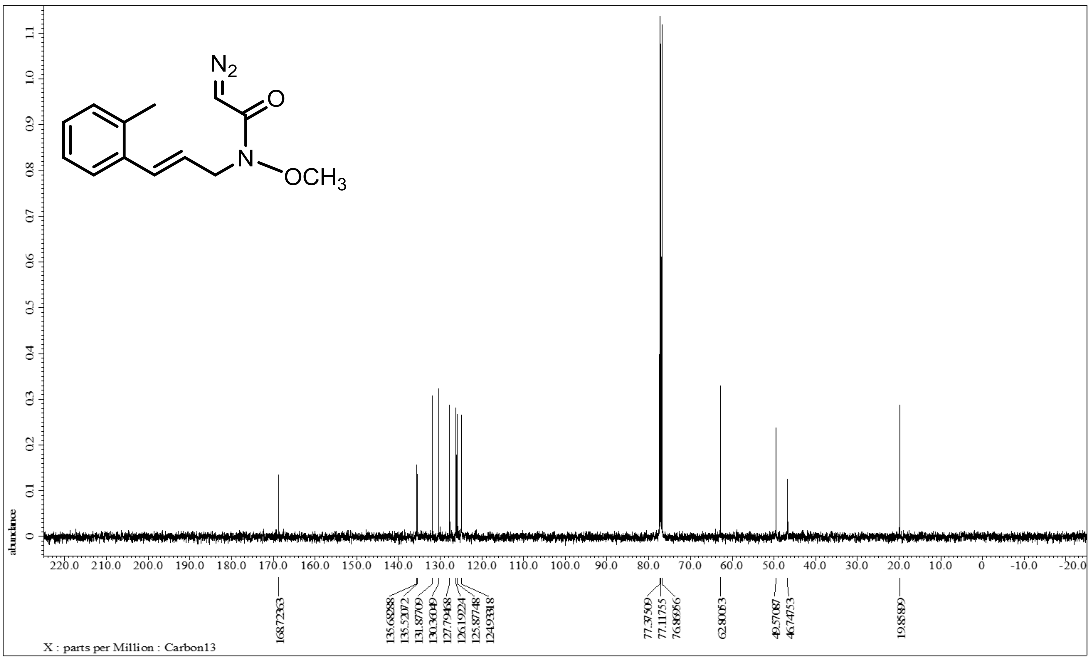

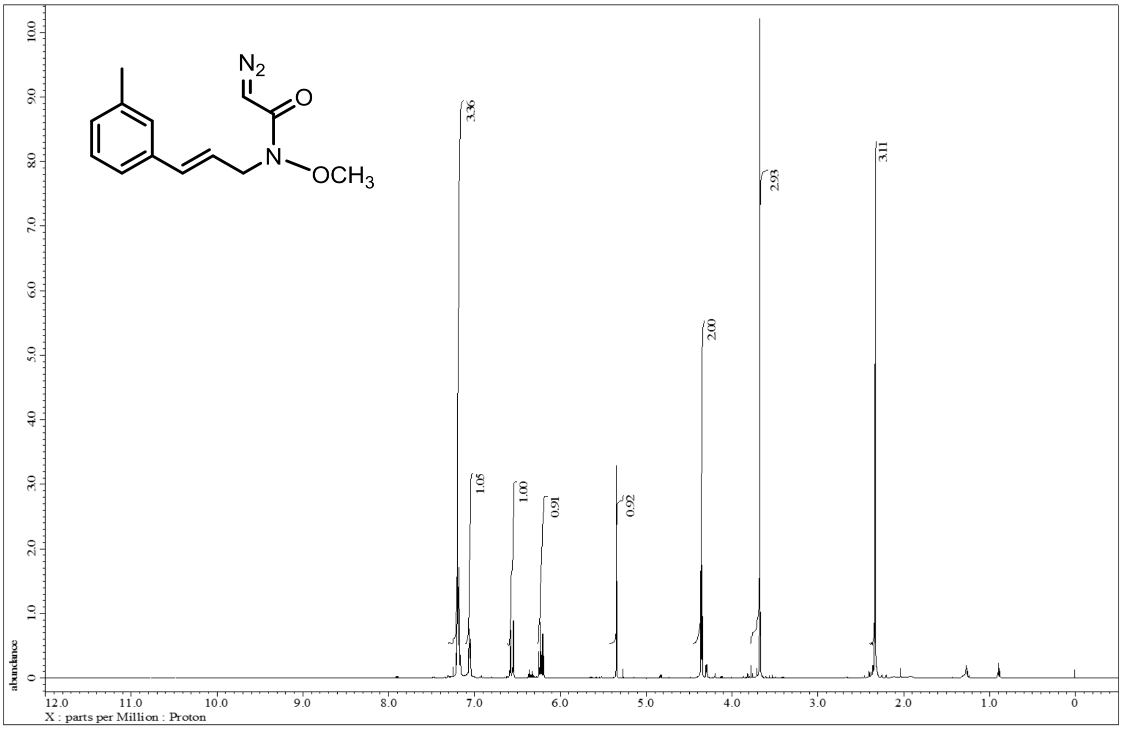
N2
O
N
OCH3
3.36
1.05
1.00
0.91
0.92
2.00
2.93
3.11
abundance
X : parts per Million : Proton

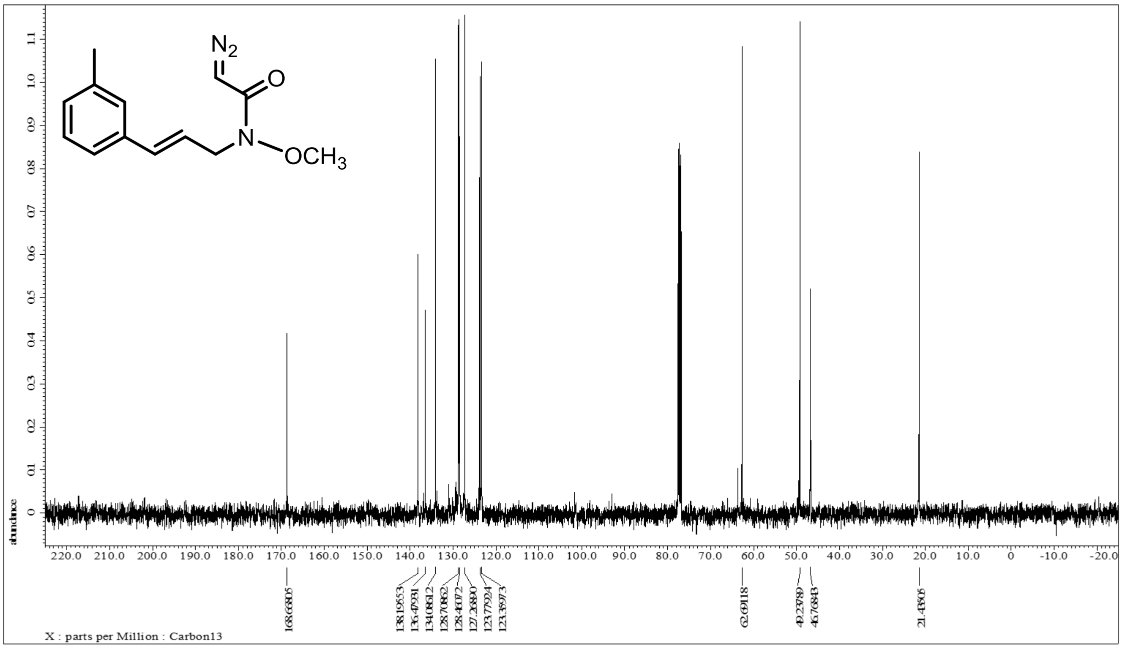
N2
O
N
OCH3
abundance
168.66805
138.19553
136.47931
134.08612
128.70862
128.46072
127.26890
123.77924
123.38973
62.69118
49.23789
46.76843
21.43805
X : parts per Million : Carbon13

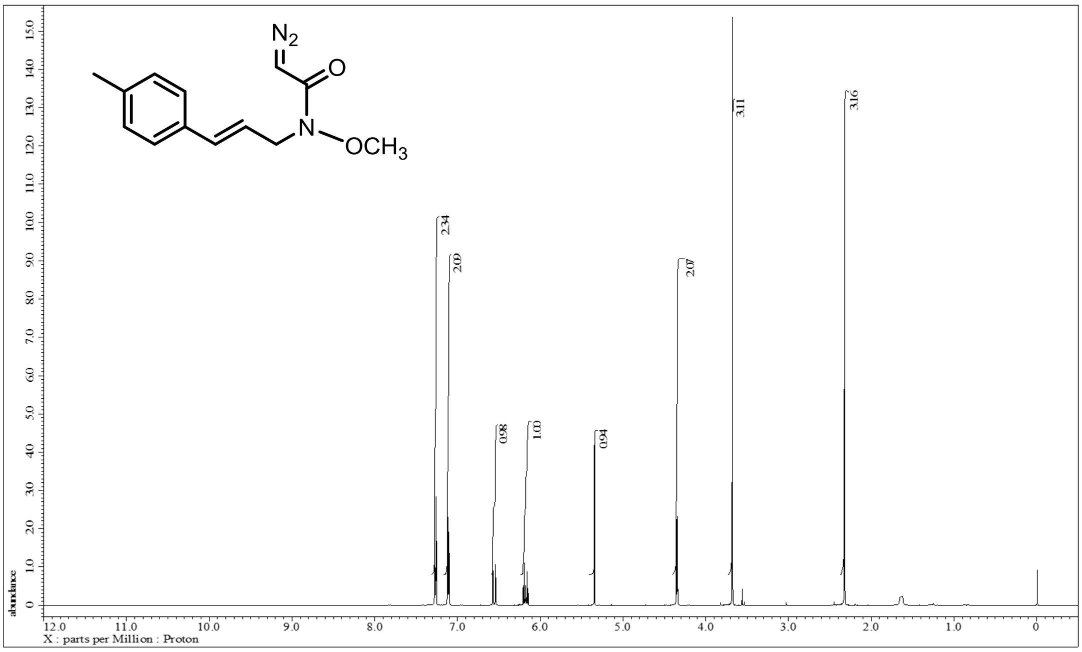
N2
O
N
OCH3
abundance
X : parts per Million : Proton
2.34
2.09
0.98
1.03
0.94
2.07
3.11
3.16

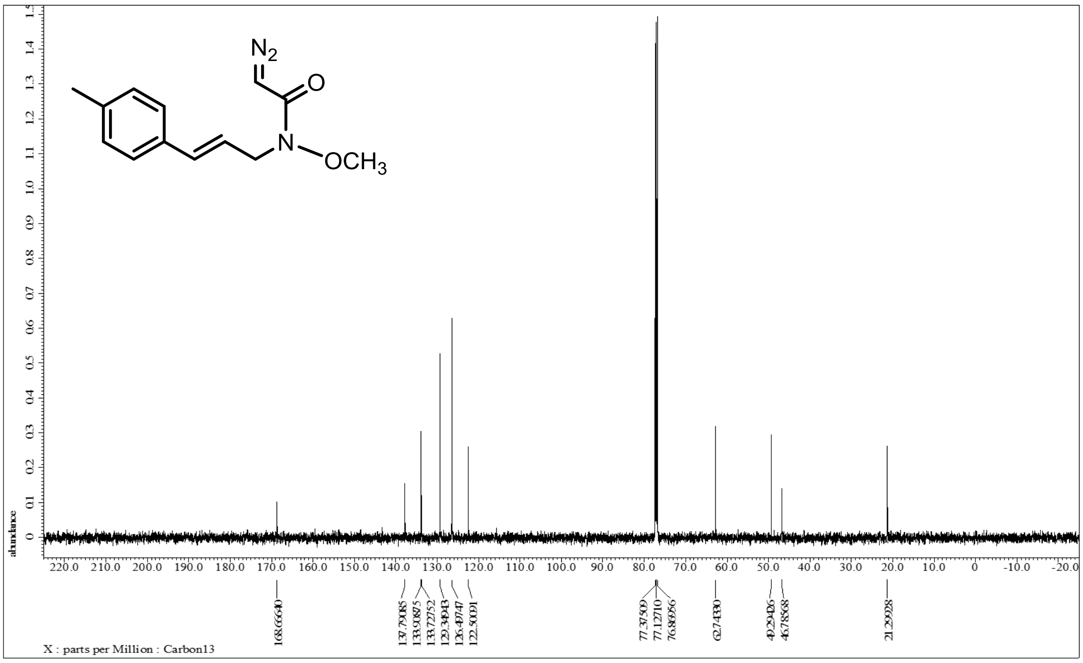
N2
O
N
OCH3
abundance
X : parts per Million : Carbon13
168.66640
137.79085
133.93875
133.72752
129.34943
126.49747
122.50091
77.37509
77.12710
76.86956
62.74330
49.29436
46.78568
21.29928

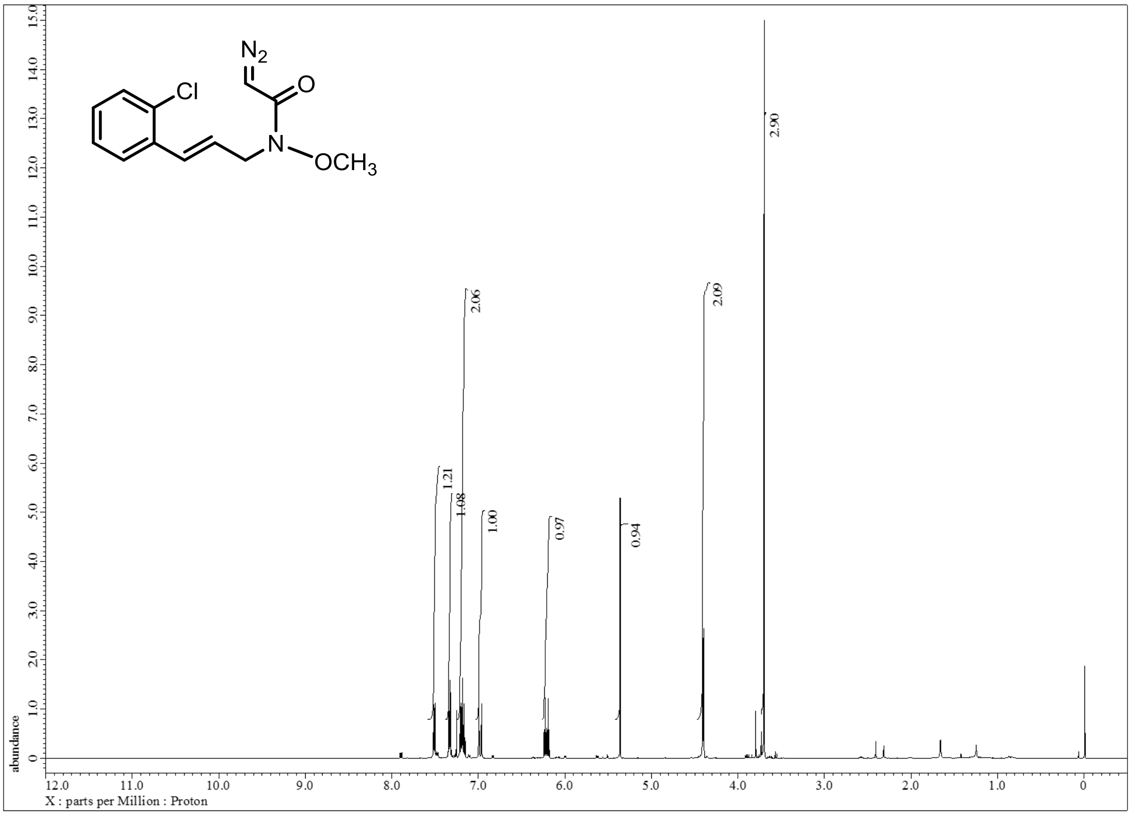
N2
Cl
O
N
OCH3
2.90
2.09
2.06
1.21
1.08
1.00
0.97
0.94
abundance
X : parts per Million : Proton

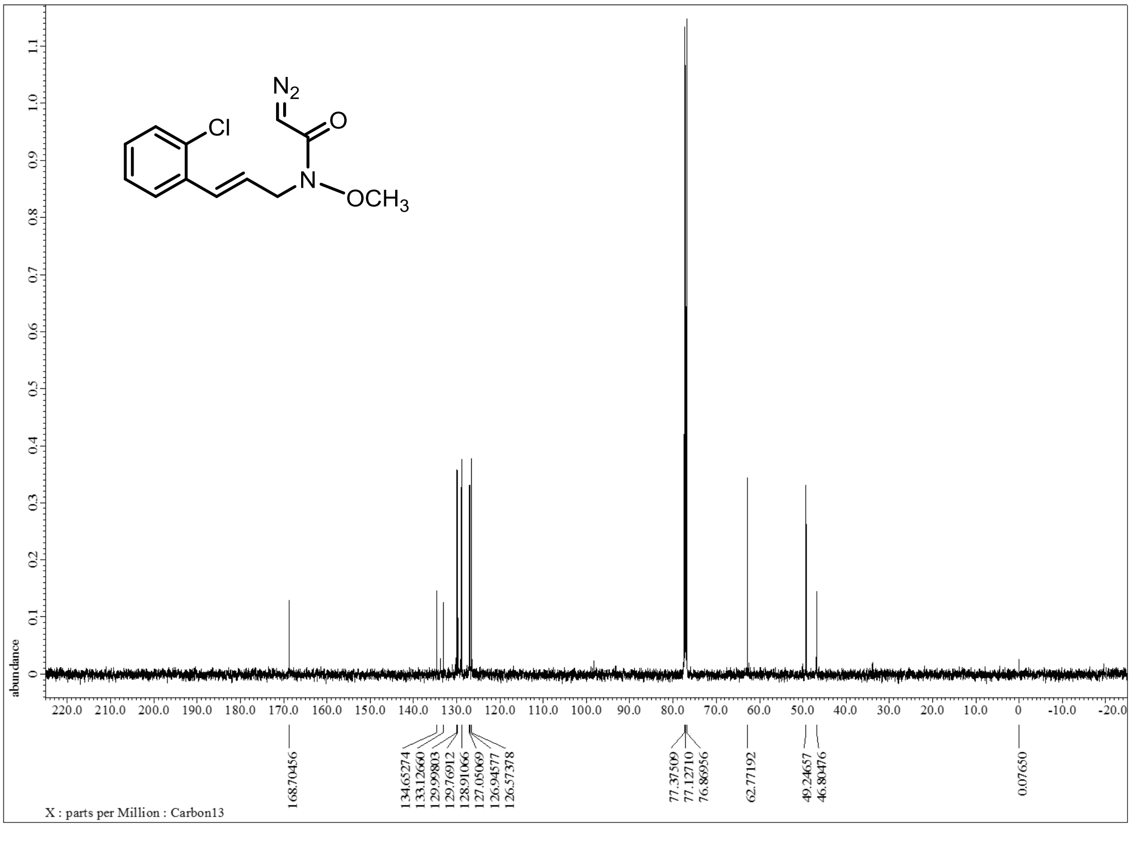
N2
Cl
O
N
OCH3
abundance
168.70456
134.65274
133.12660
129.99803
129.76912
128.91066
127.05069
126.94577
126.57378
77.37509
77.12710
76.86956
62.77192
49.24657
46.80476
0.07650
X : parts per Million : Carbon13

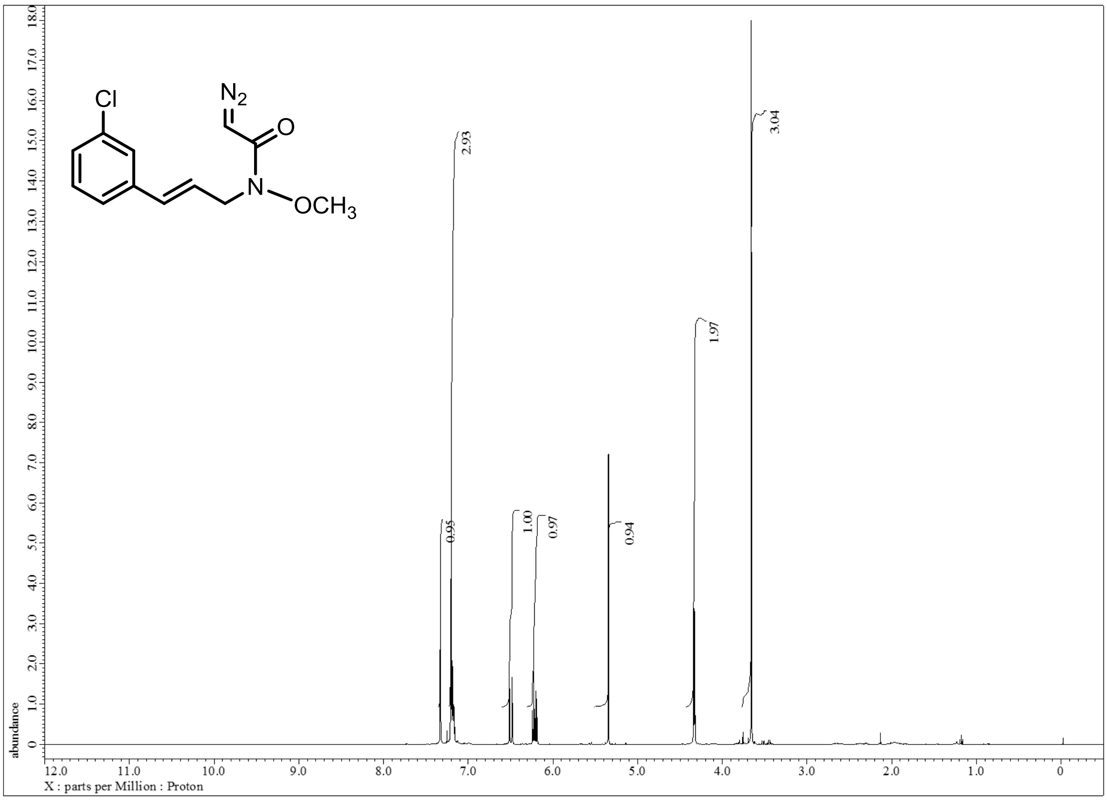
Cl
N2
O
N
OCH3
abundance
X : parts per Million : Proton
2.93
3.04
1.97
0.95
1.00
0.97
0.94

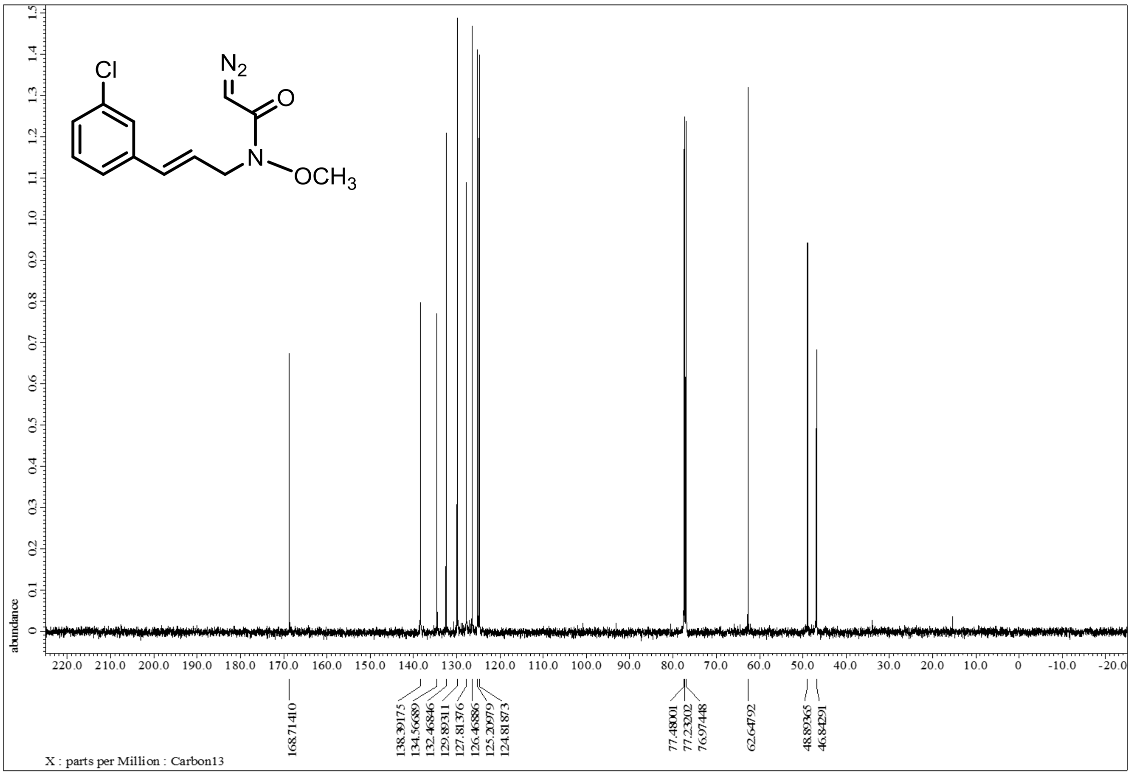
Cl
N2
O
N
OCH3
abundance
X : parts per Million : Carbon13
168.71410
138.39175
134.56689
132.46846
129.89311
127.81376
126.46886
125.20979
124.81873
77.48001
77.23202
76.97448
62.64792
48.89365
46.84291

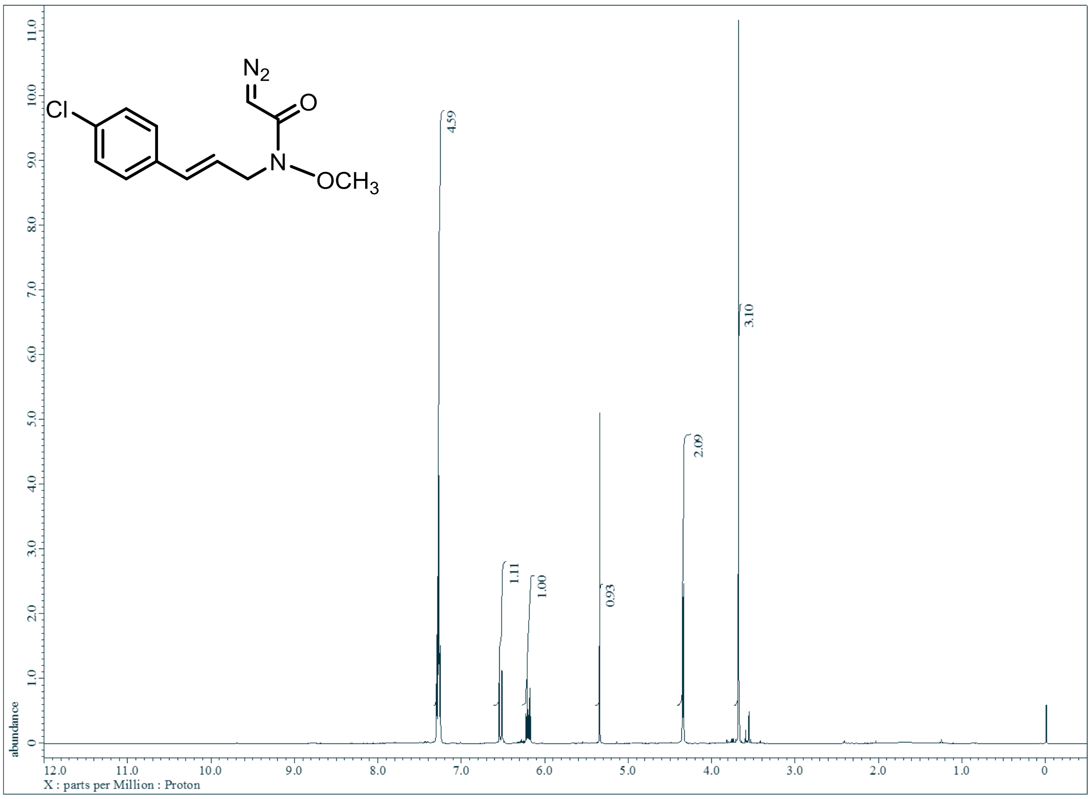
N2
O
Cl
N
OCH3
4.59
3.10
2.09
1.11
1.00
0.93
abundance
12.0
11.0
10.0
9.0
8.0
7.0
6.0
5.0
4.0
3.0
2.0
1.0
0
X : parts per Million : Proton

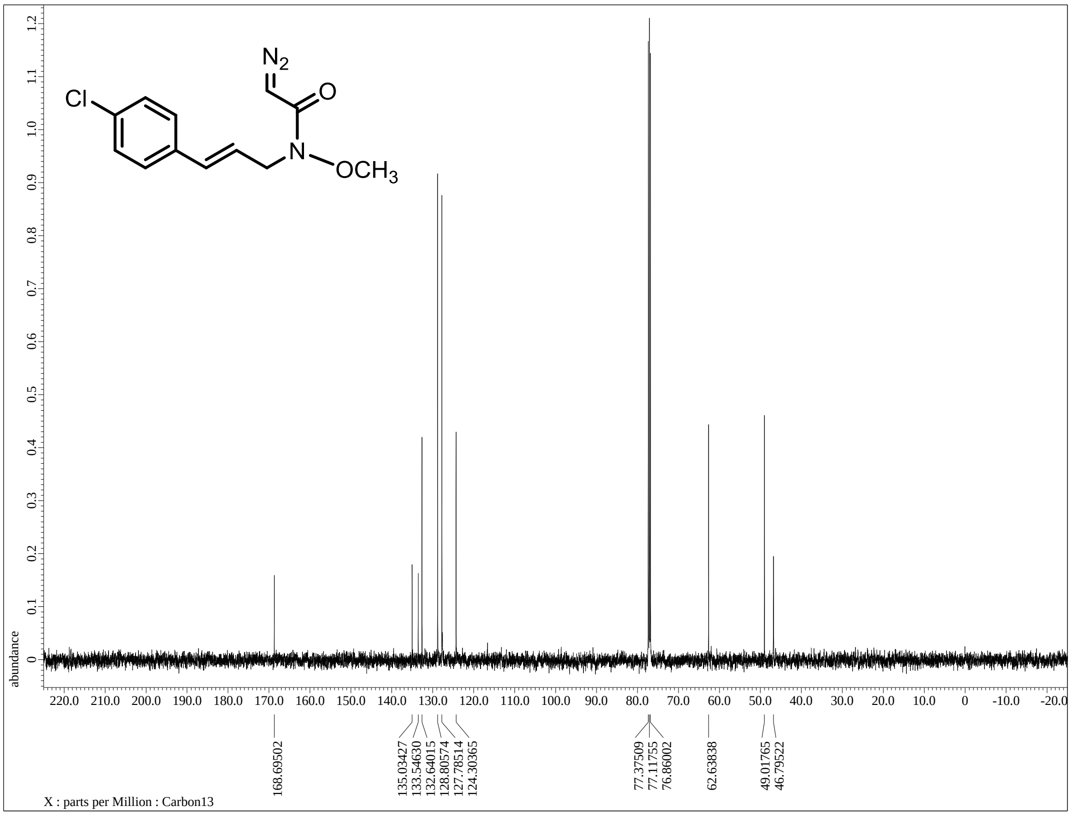
N2
O
Cl
N
OCH3
abundance
168.69502
135.03427
133.54630
132.64015
128.80574
127.78514
124.30365
77.37509
77.11755
76.86002
62.63838
49.01765
46.79522
X : parts per Million : Carbon13

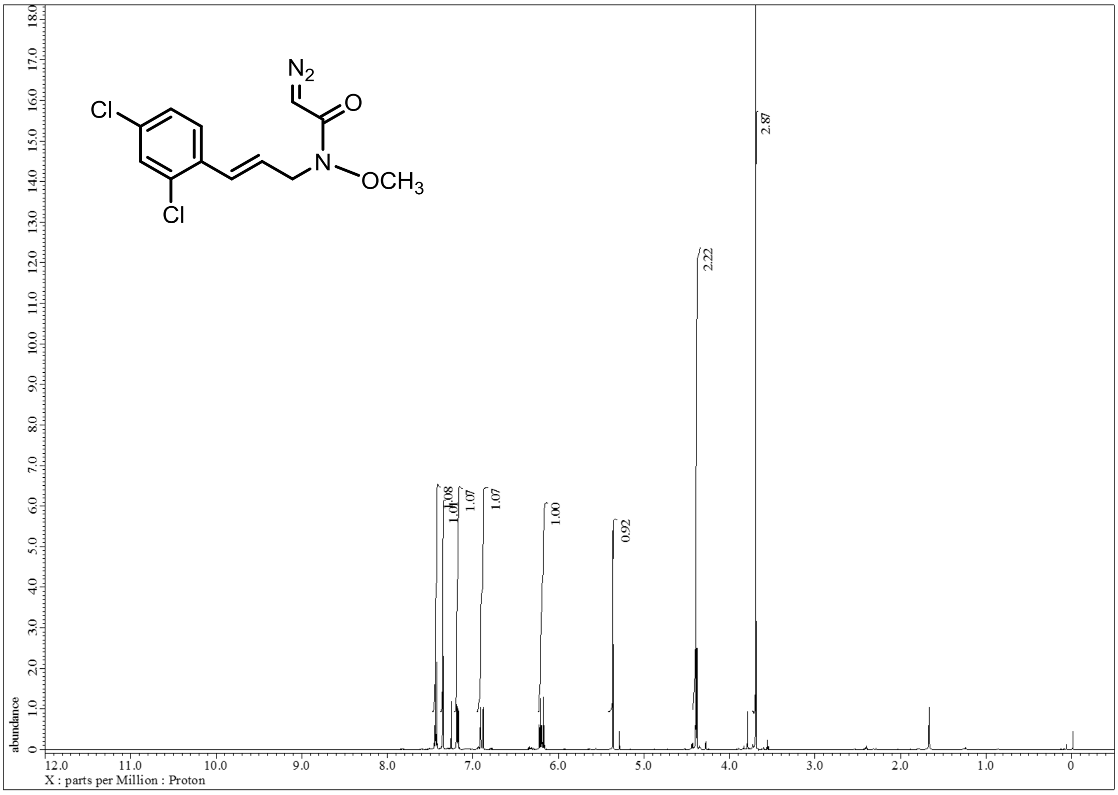
N2
O
Cl
Cl
N
OCH3
abundance
X : parts per Million : Proton
2.87
2.22
0.92
1.00
1.07
1.07
1.08
1.01

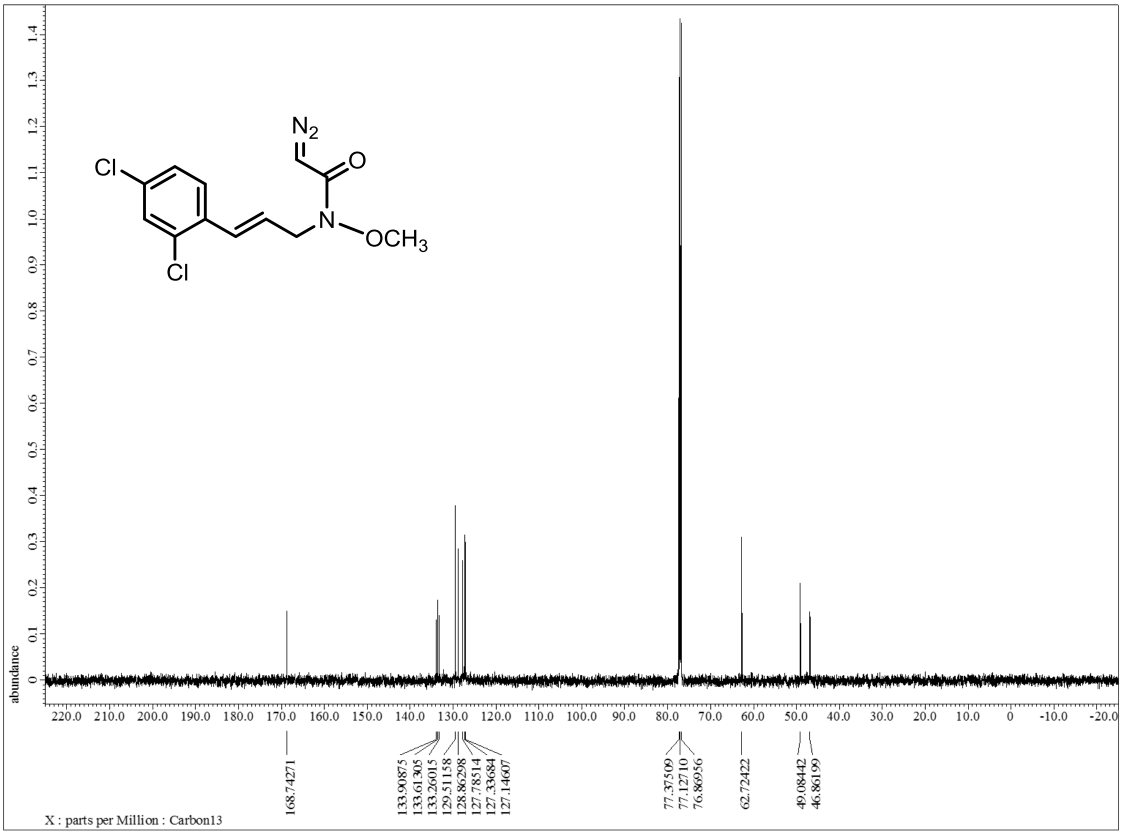
N2
O
Cl
Cl
N
OCH3
abundance
X : parts per Million : Carbon13
168.74271
133.90875
133.61305
133.26015
129.51158
128.86298
127.78514
127.33684
127.14607
77.37509
77.12710
76.86956
62.72422
49.08442
46.86199

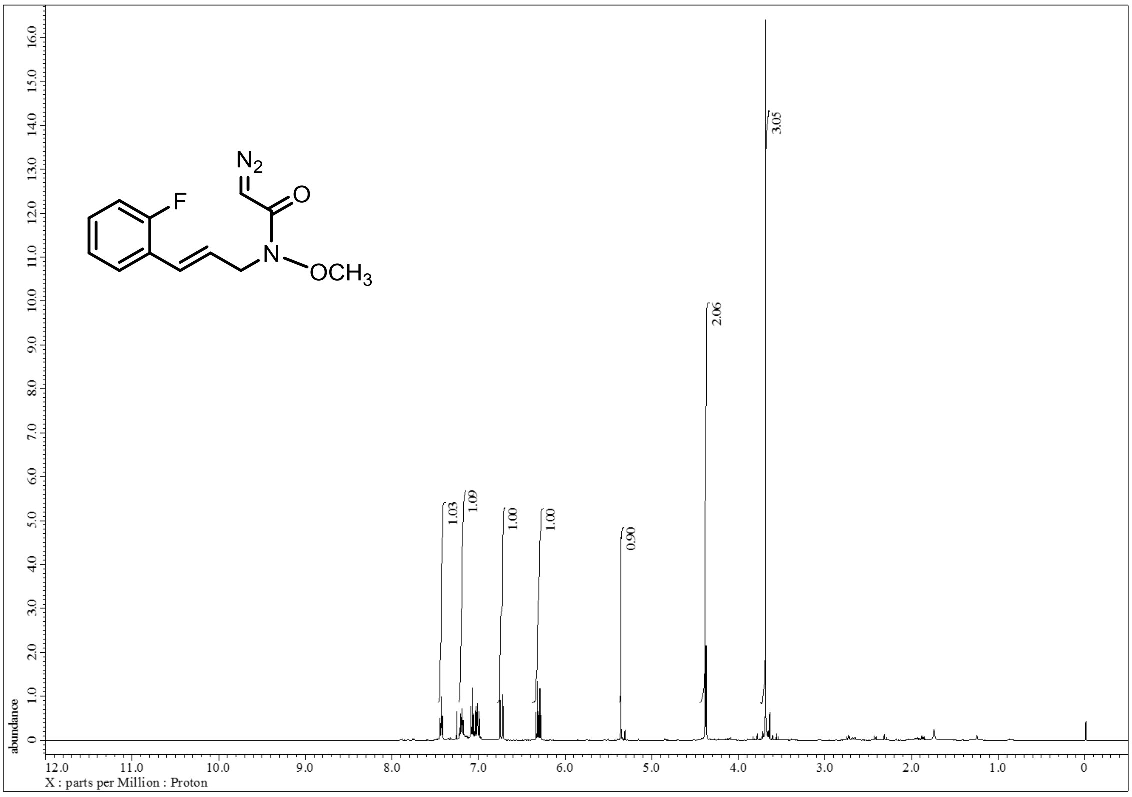
N2
F
O
N
OCH3
abundance
X : parts per Million : Proton
1.03
1.09
1.00
1.00
0.90
2.06
3.05

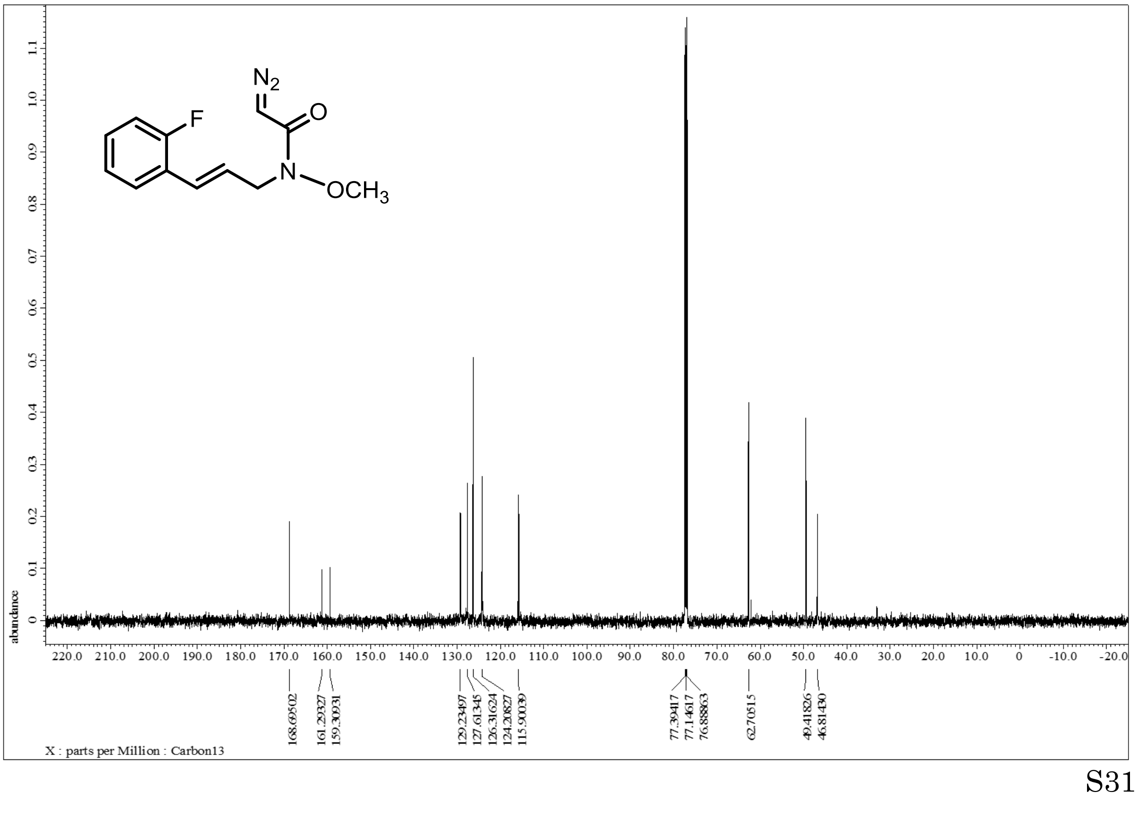
N2
F
O
N
OCH3
abundance
X : parts per Million : Carbon13
168.69502
161.29327
159.30931
129.23497
127.61345
126.31624
124.20827
115.90039
77.39417
77.14617
76.88863
62.70515
49.41826
46.81430

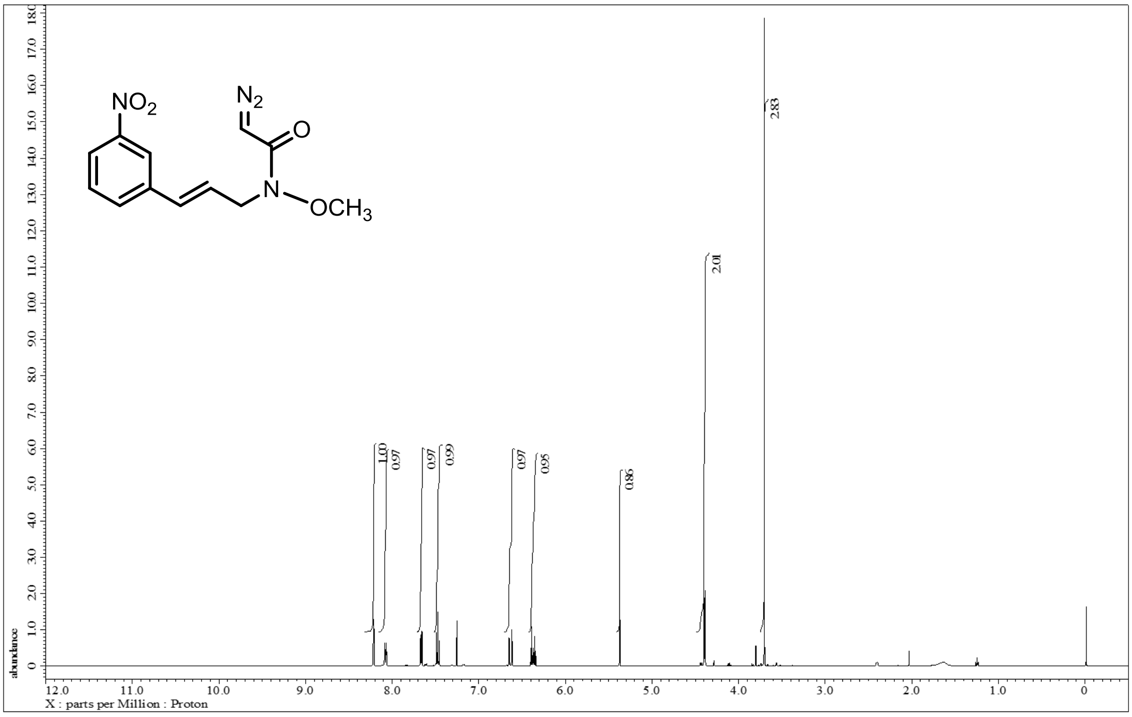
NO2
N2
O
N
OCH3
X : parts per Million : Proton
abundance
1.00
0.97
0.97
0.99
0.97
0.95
0.86
2.01
2.83

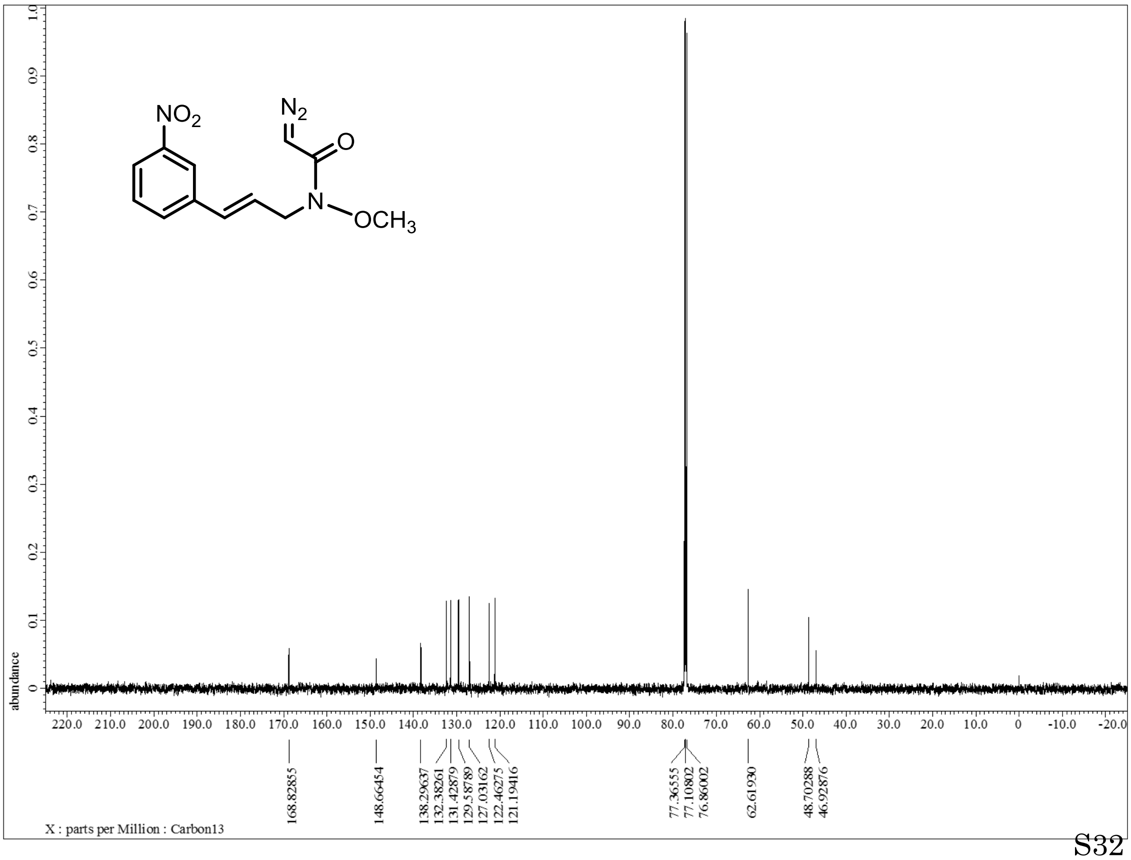
NO2
N2
O
N
OCH3
abundance
X : parts per Million : Carbon13
168.82855
148.66454
138.29637
132.38261
131.42879
129.58789
127.03162
122.46275
121.19416
77.36555
77.10802
76.86002
62.61930
48.70288
46.92876

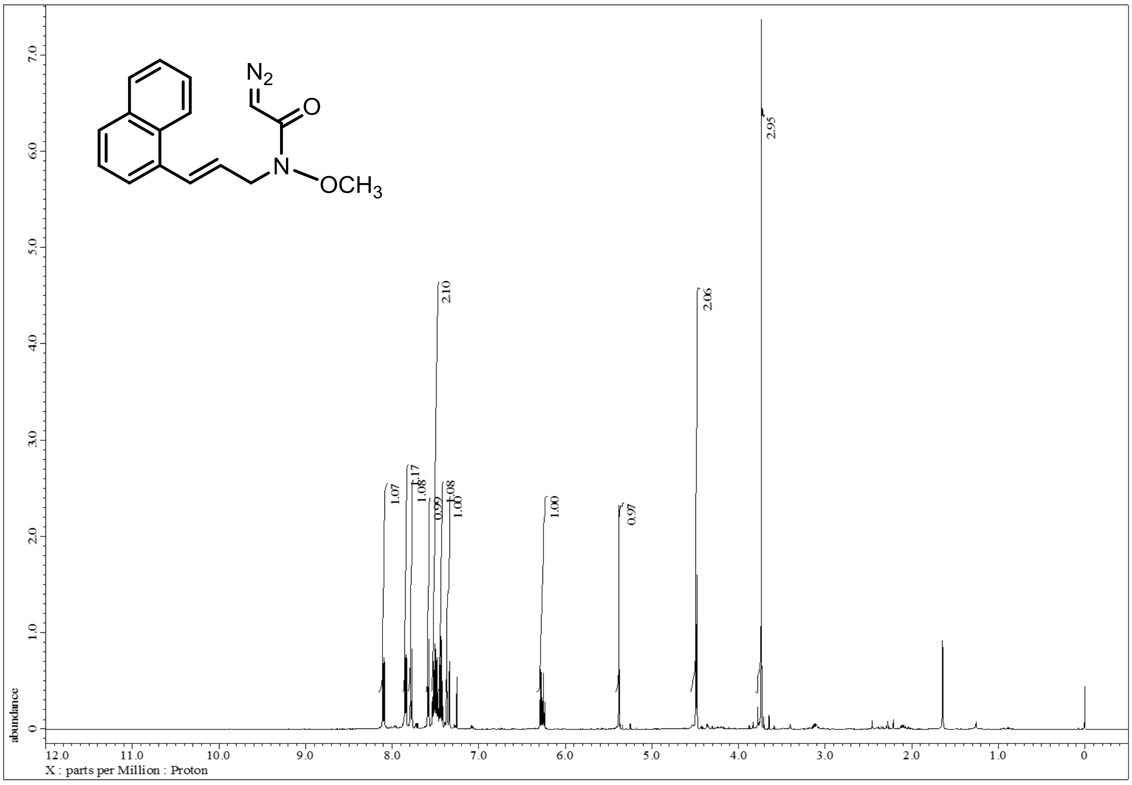
N2
O
N
OCH3
abundance
1.07
1.17
1.08
0.92
1.08
1.00
1.00
0.97
2.10
2.06
2.95
X : parts per Million : Proton

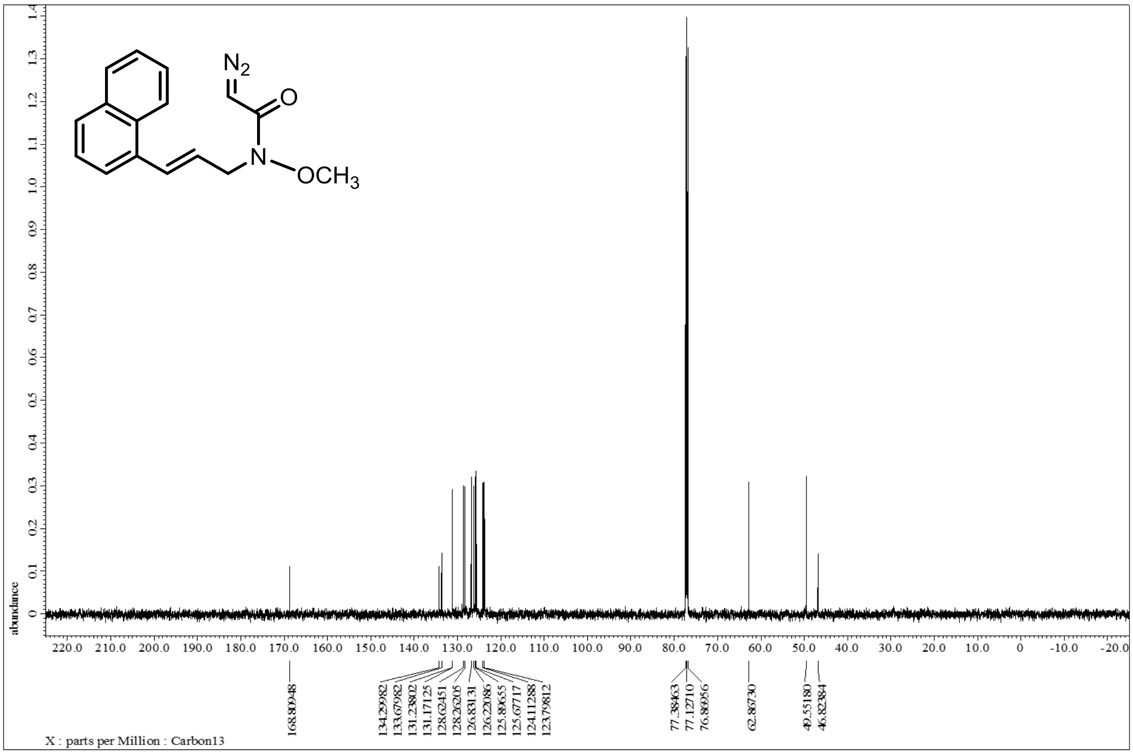
N2
O
N
OCH3
abundance
168.80948
134.29982
133.67982
131.23802
131.17125
128.62451
128.26205
126.83131
126.22086
125.89655
125.67717
124.11288
123.79812
77.38463
77.12710
76.86956
62.86730
49.55180
46.82384
X : parts per Million : Carbon13

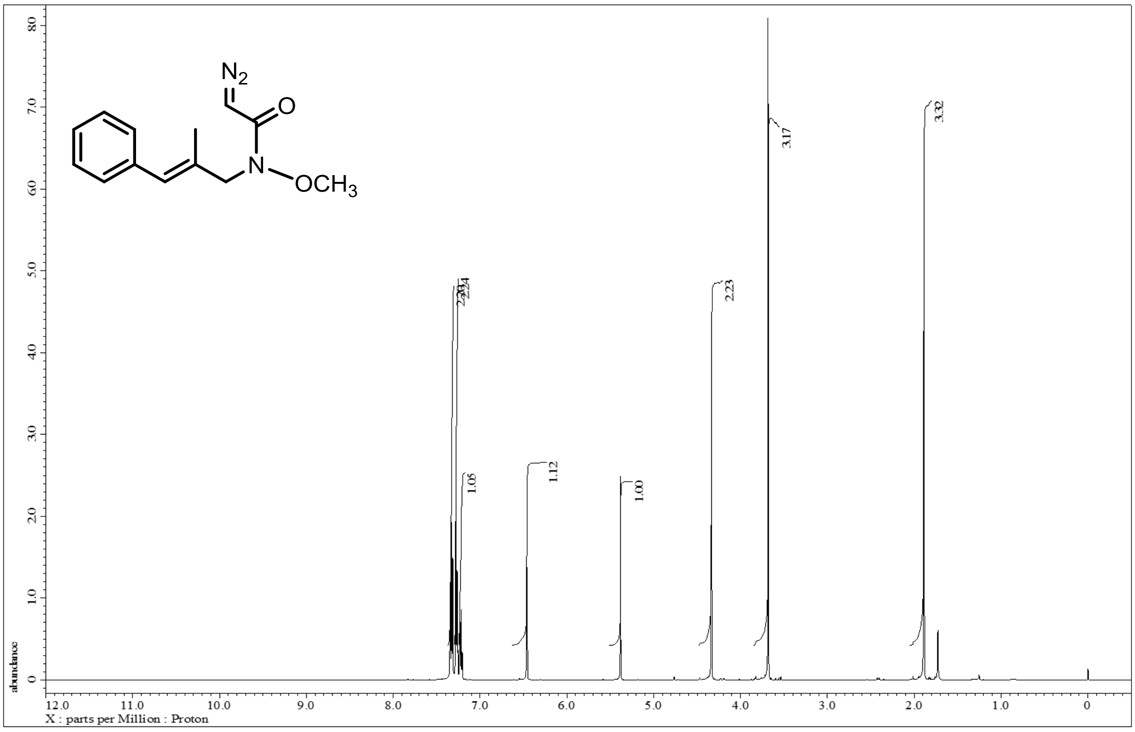
N2
O
N
OCH3
abundance
X : parts per Million : Proton
3.17
3.32
2.23
1.12
1.00
1.05

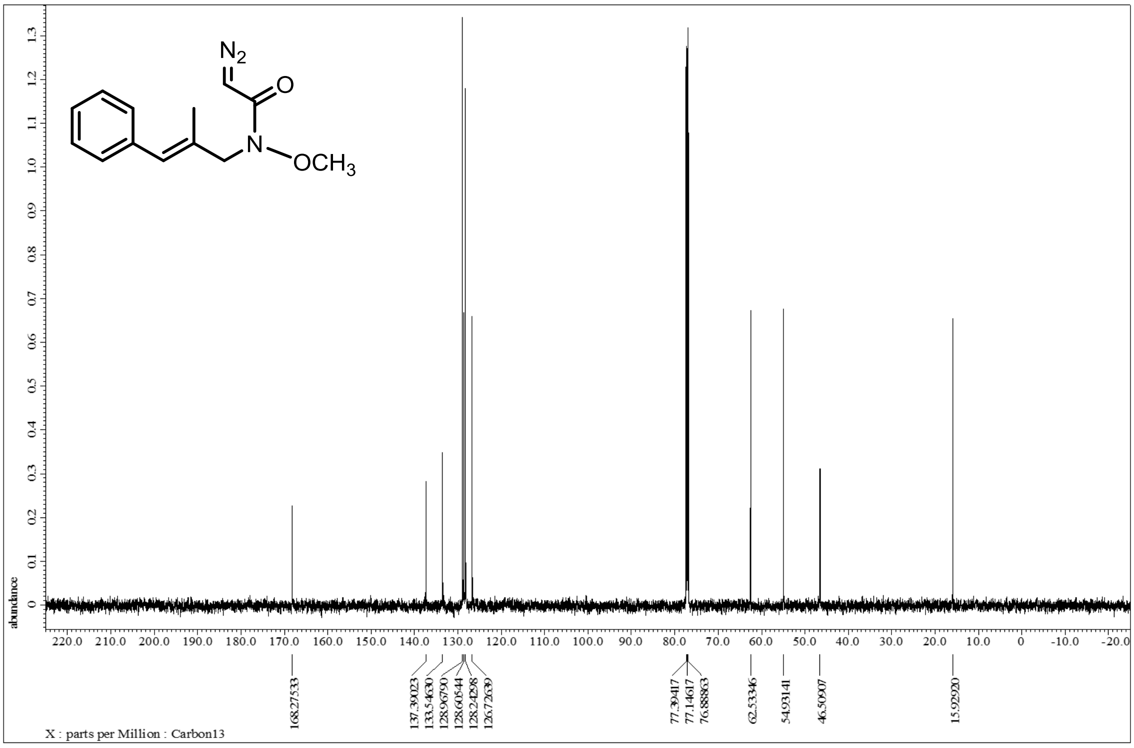
N2
O
N
OCH3
abundance
X : parts per Million : Carbon13
168.27533
137.39023
133.54630
128.96790
128.60544
128.24298
126.72639
77.39417
77.14617
76.88863
62.53346
54.93141
46.50907
15.92920

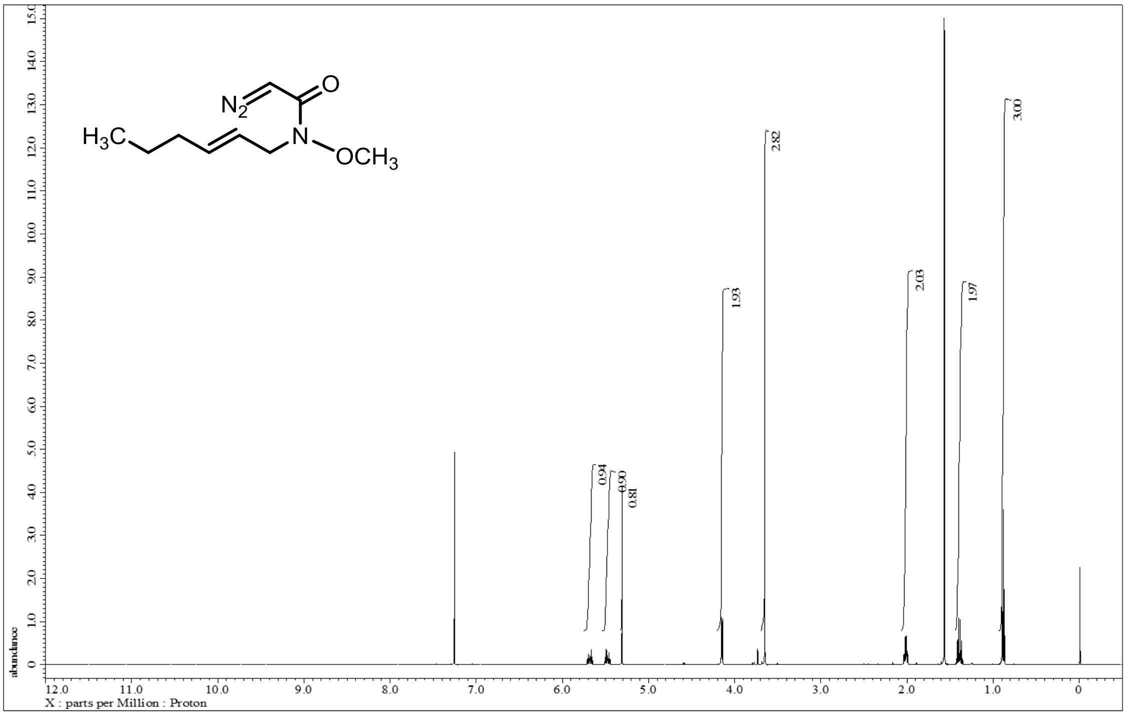
X : parts per Million : Proton
abundance

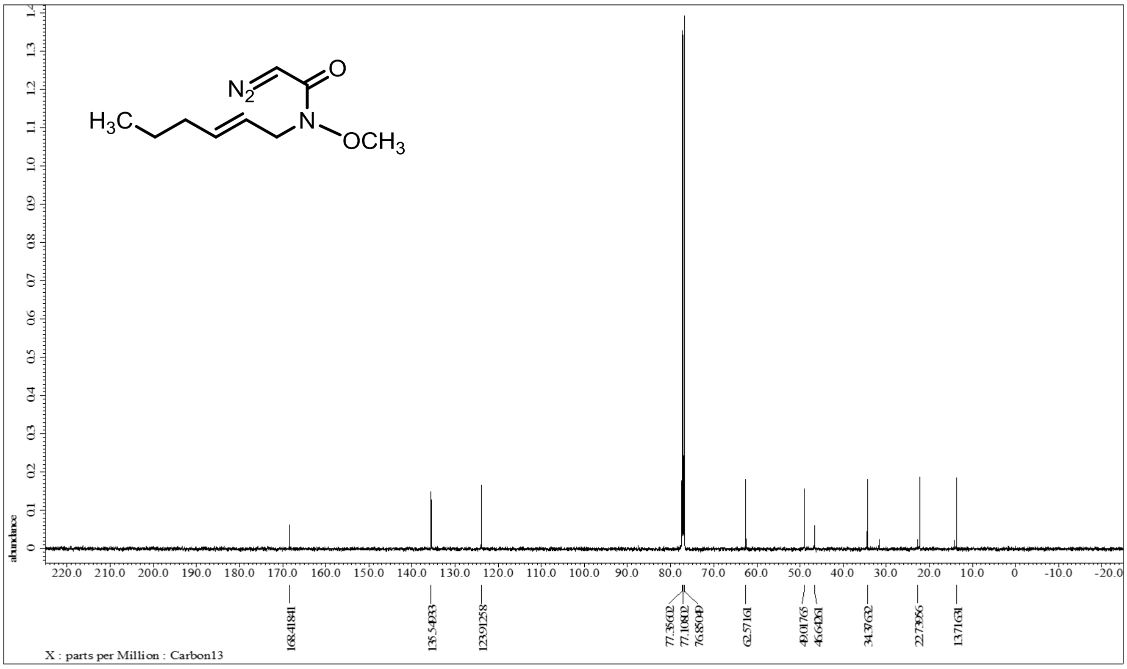
X : parts per Million : Carbon13
abundance

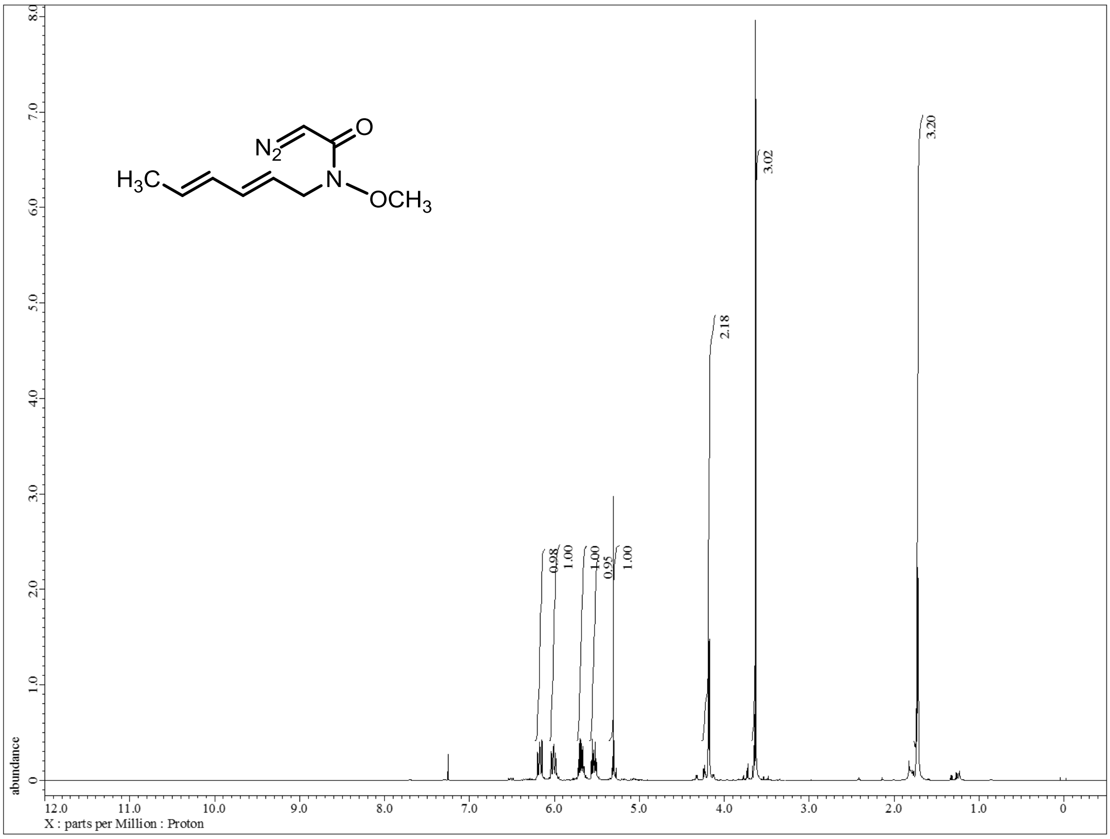

H3C
N2
O
N
OCH3
abundance
X : parts per Million : Proton
0.98
1.00
1.00
0.95
1.00
2.18
3.02
3.20

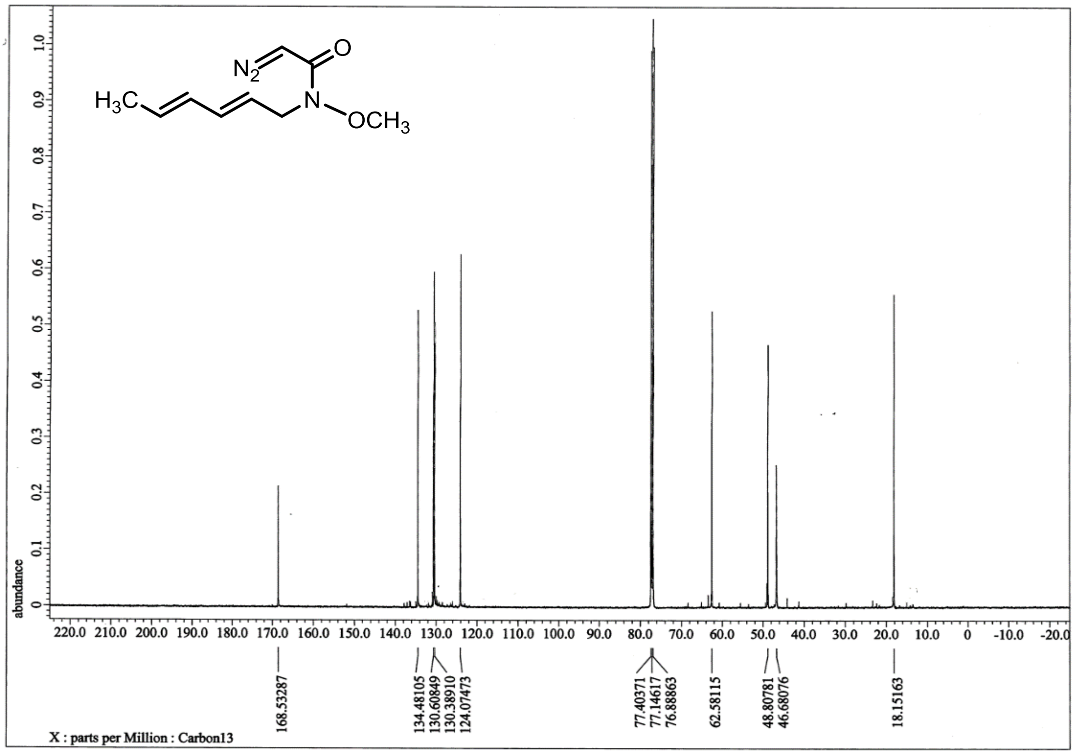

H3C
N2
O
N
OCH3
abundance
X : parts per Million : Carbon13
168.53287
134.48105
130.60849
130.38910
124.07473
77.40371
77.14617
76.88863
62.58115
48.80781
46.68076
18.15163

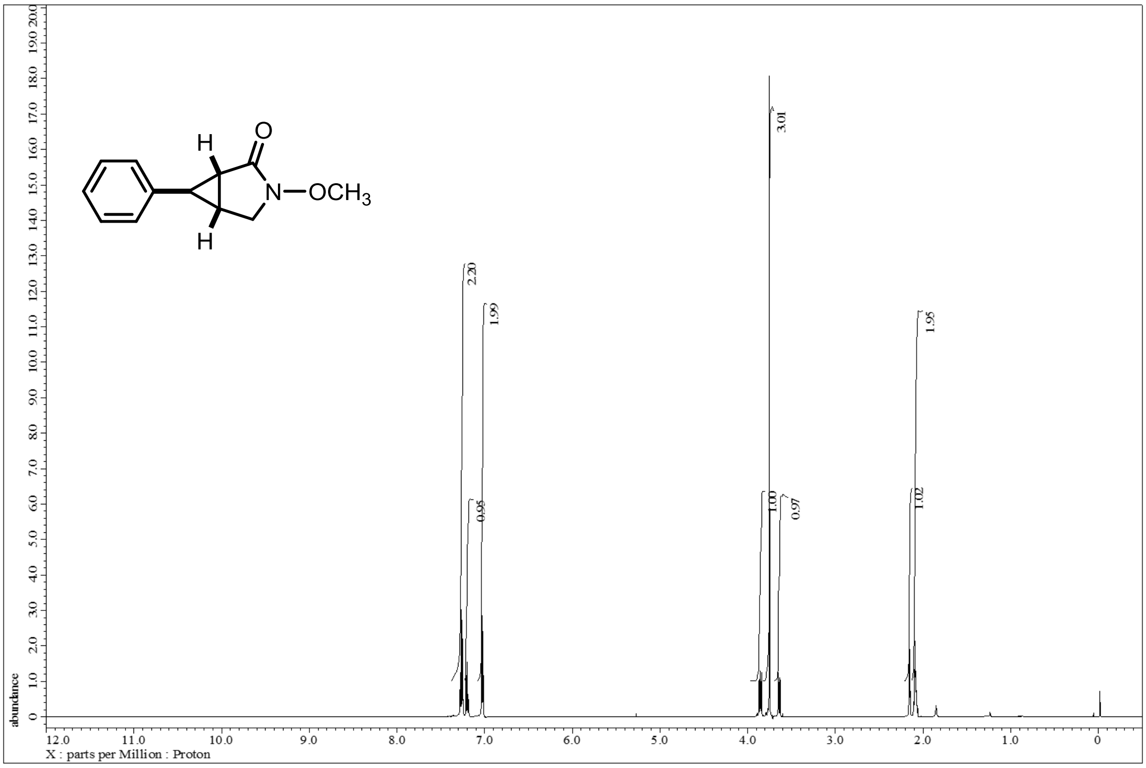
H
O
N—OCH3
H
2.20
1.99
0.95
3.01
1.00
0.97
1.95
1.02
abundance
X : parts per Million : Proton

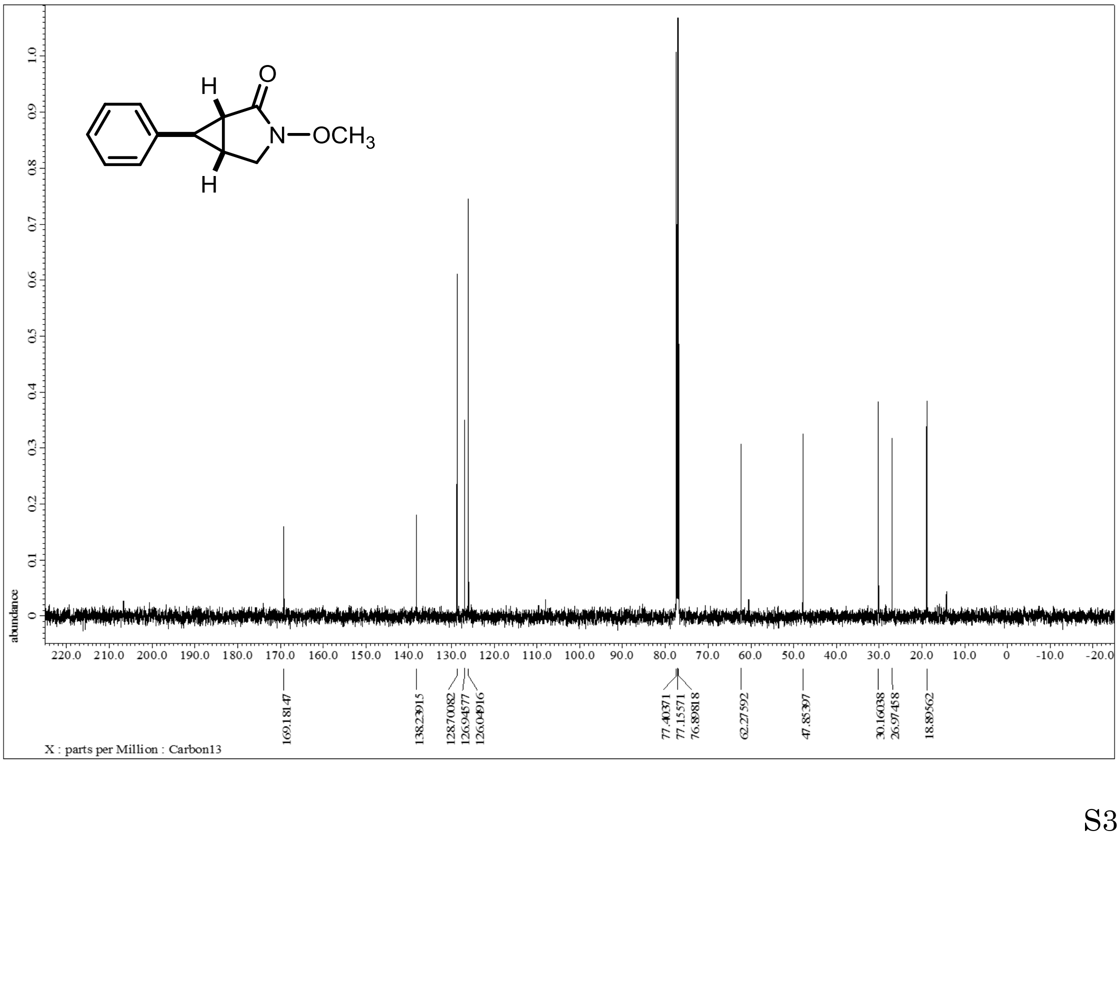
H
O
N—OCH3
H
169.18147
138.23915
128.70082
126.94577
126.04916
77.40371
77.15571
76.89818
62.27592
47.85397
30.16038
26.97458
18.89562
abundance
X : parts per Million : Carbon13

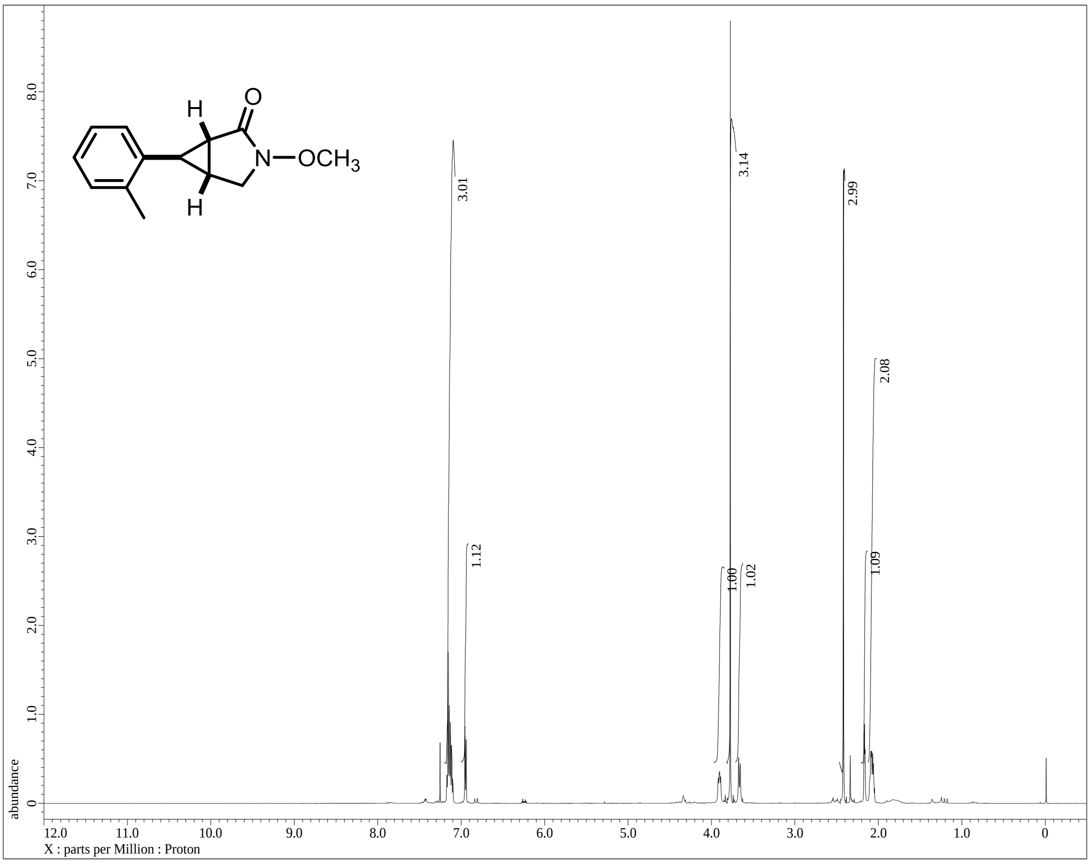
H
O
N—OCH3
H
3.01
1.12
3.14
1.00
1.02
2.99
2.08
1.09
abundance
X : parts per Million : Proton

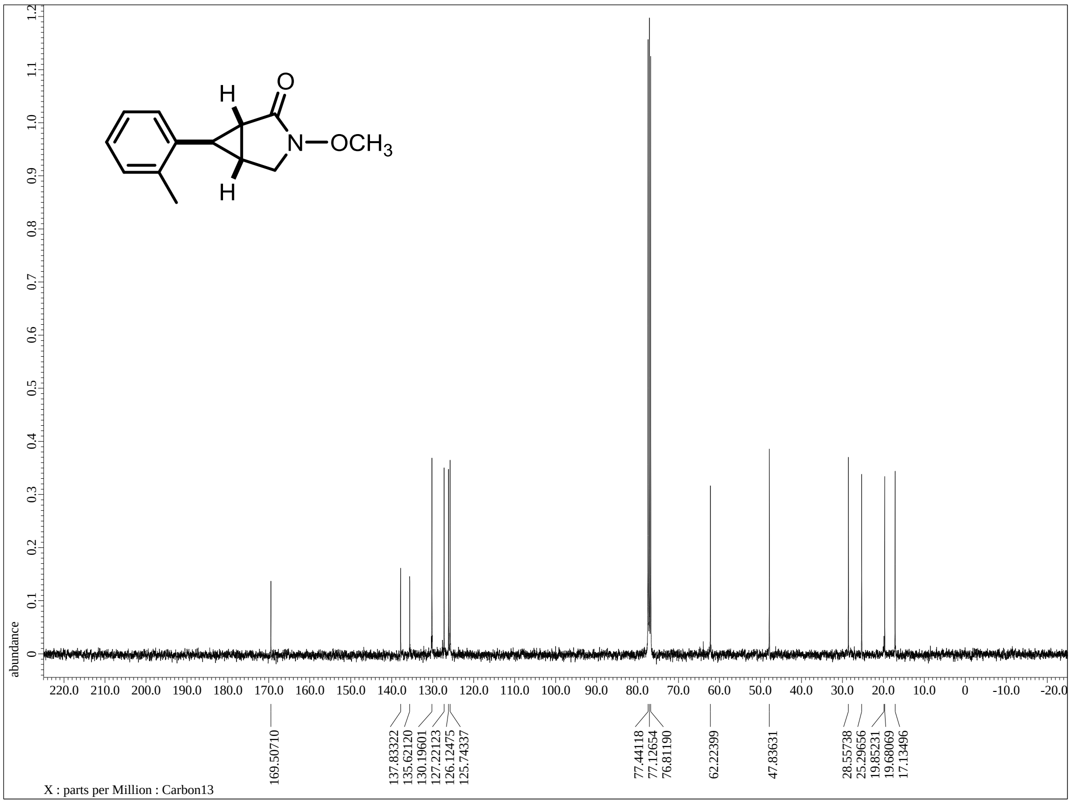
H
O
N—OCH3
H
abundance
169.50710
137.83322
135.62120
130.19601
127.22123
126.12475
125.74337
77.44118
77.12654
76.81190
62.22399
47.83631
28.55738
25.29656
19.85231
19.68069
17.13496
X : parts per Million : Carbon13

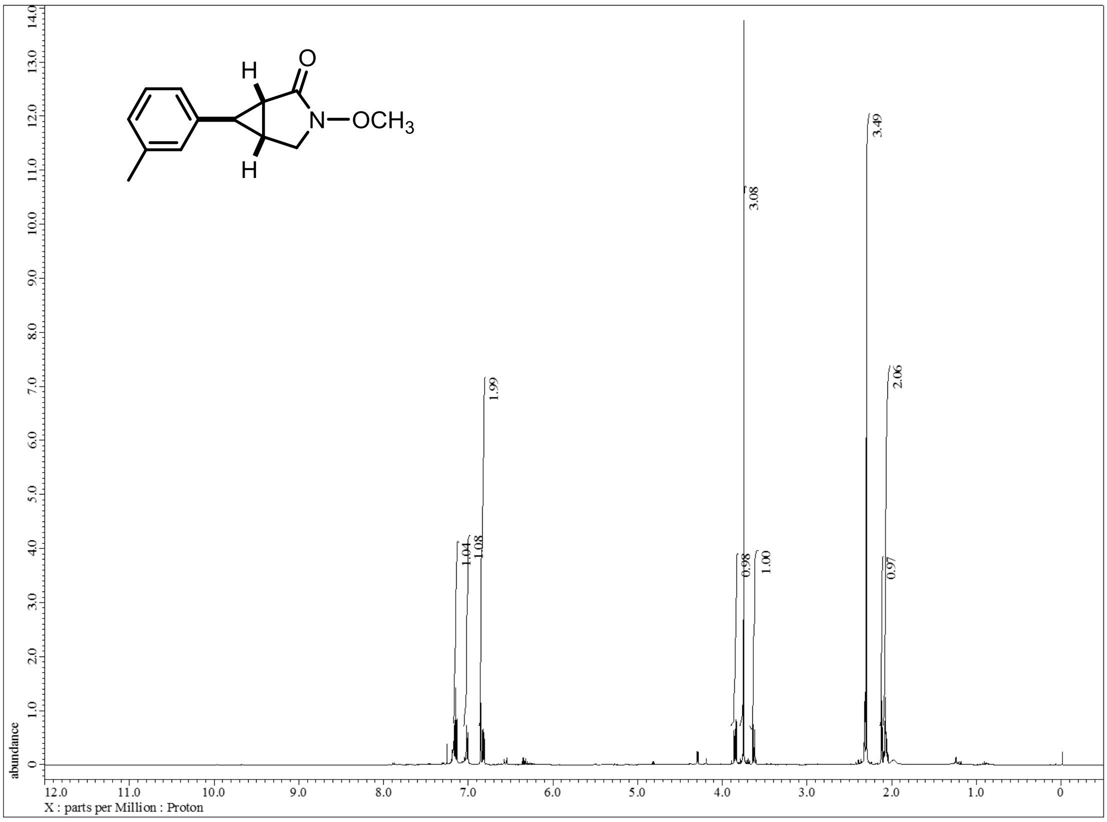

OCH3
X : parts per Million : Proton
abundance

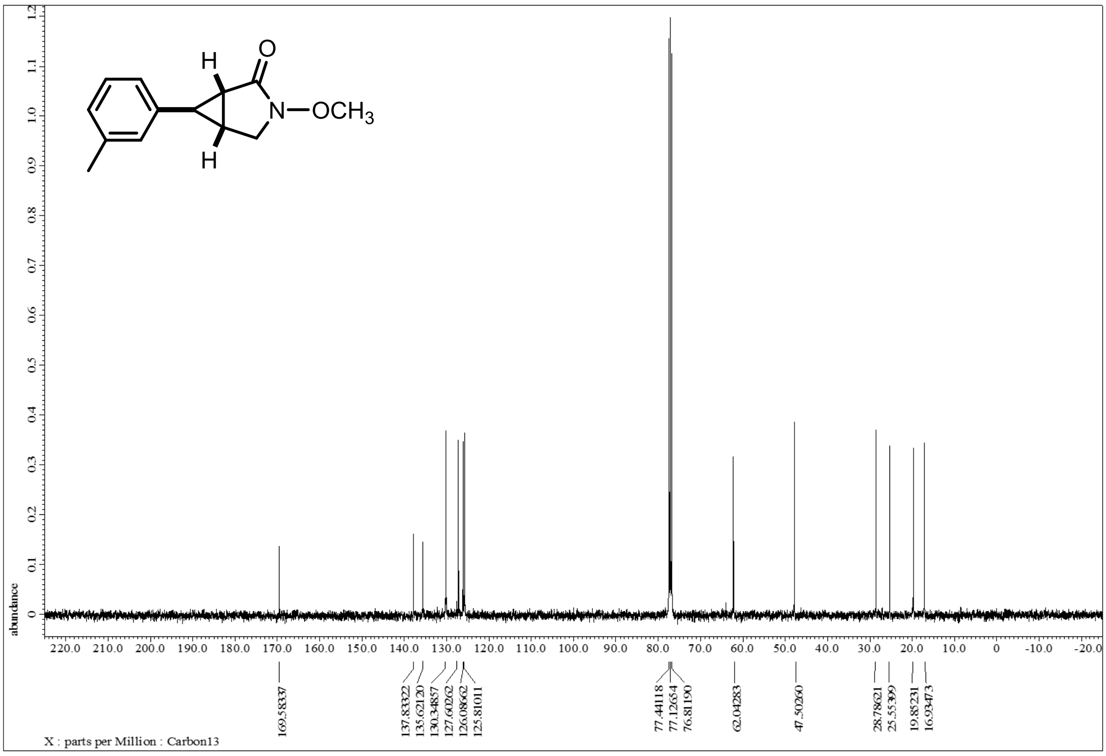

OCH3
X : parts per Million : Carbon13
abundance
169.58337
137.83322
135.62120
130.34857
127.60262
126.08662
125.81011
77.44118
77.12654
76.81190
62.04283
47.50260
28.78621
25.55309
19.85231
16.93473

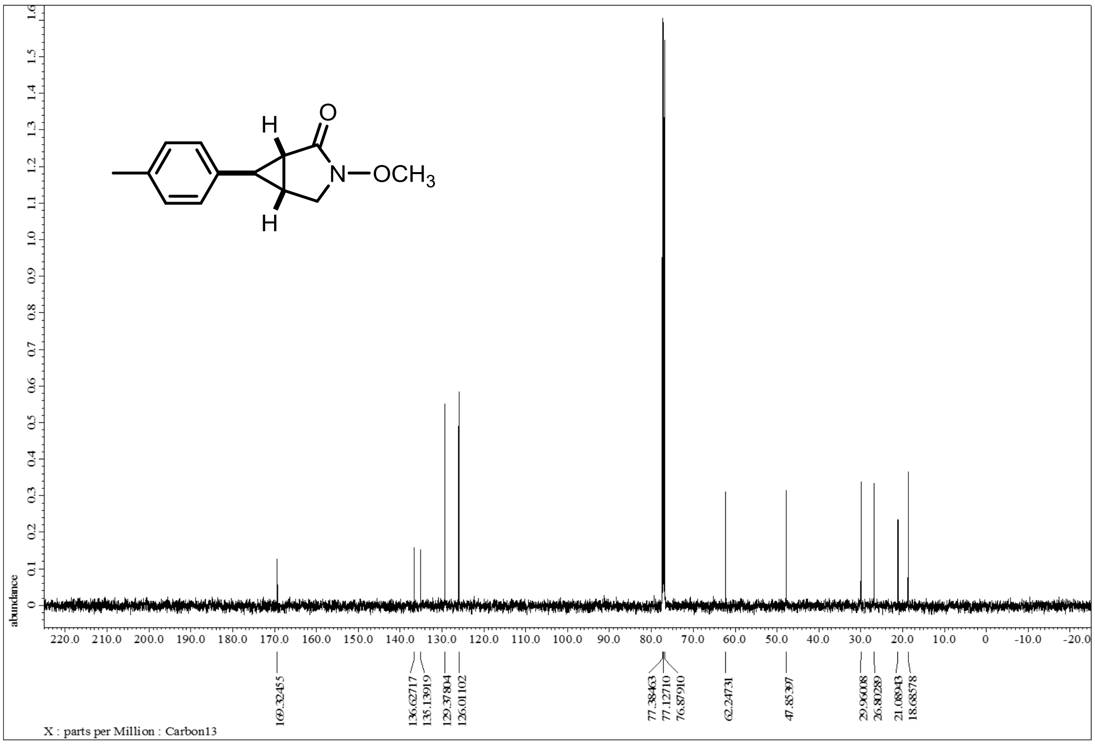
H
O
N—OCH3
H
abundance
X : parts per Million : Carbon13
169.32455
136.62717
135.13919
129.37804
126.01102
77.38463
77.12710
76.87910
62.24731
47.85397
29.96008
26.80289
21.08943
18.68578

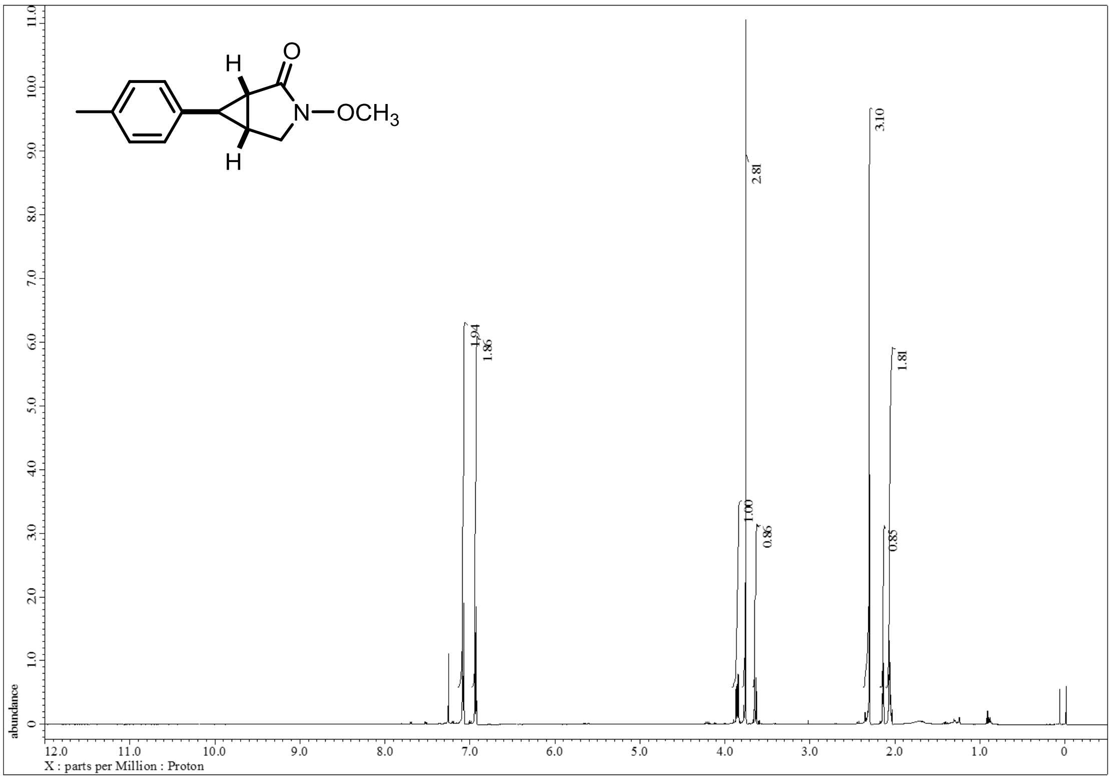
H
O
N—OCH3
H
abundance
X : parts per Million : Proton
1.94
1.86
2.81
1.00
0.86
3.10
1.81
0.85

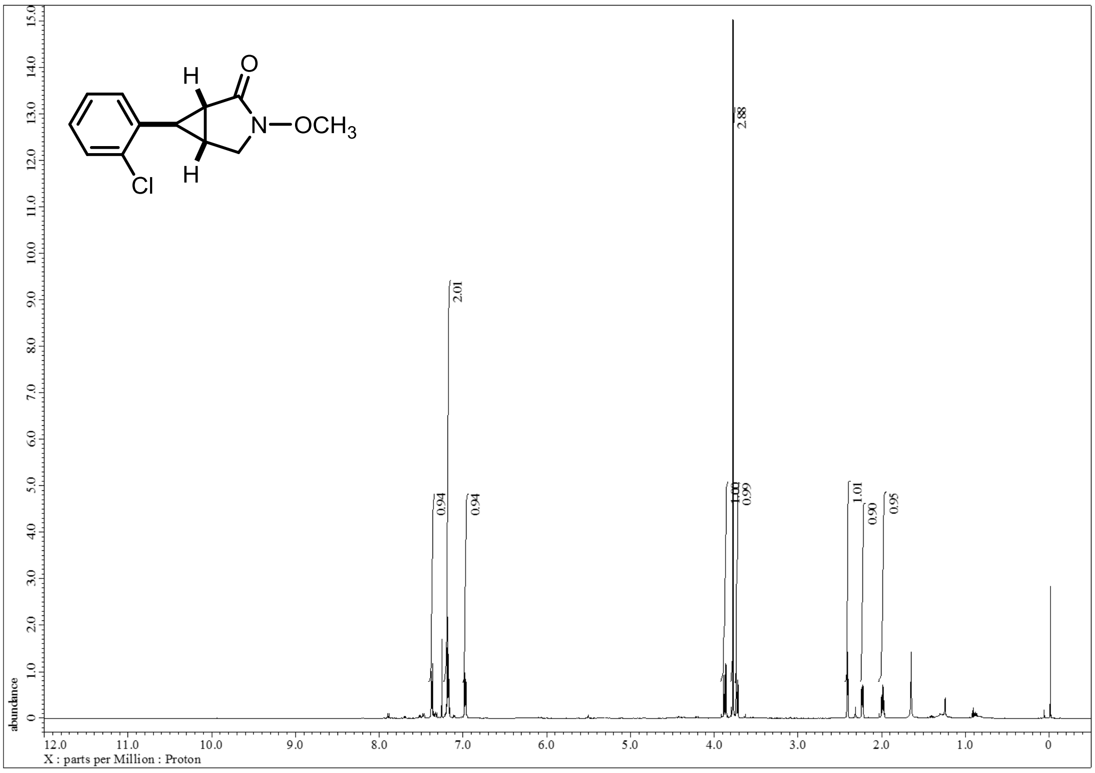

O
H
N—OCH3
Cl
H
abundance
X : parts per Million : Proton
2.88
2.01
0.94
0.94
1.01
0.90
0.95
0.99

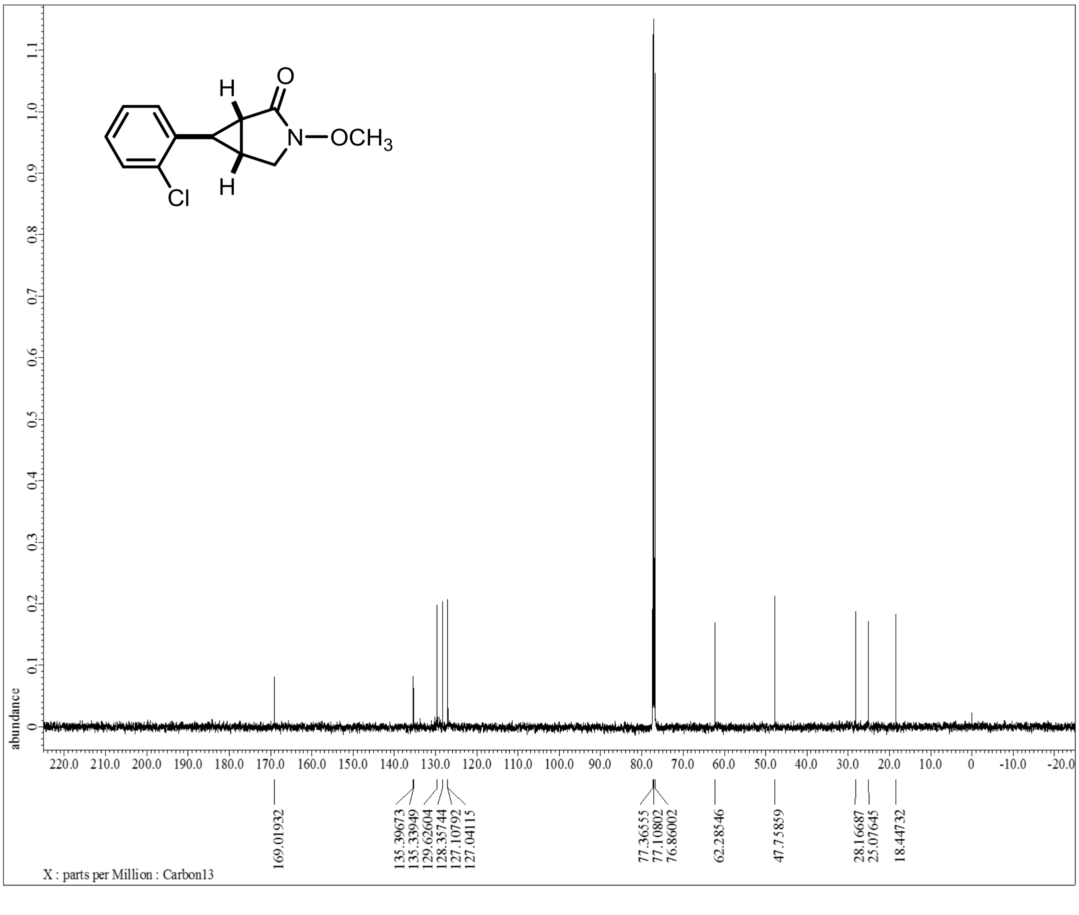

O
H
N—OCH3
Cl
H
abundance
X : parts per Million : Carbon13
169.01932
135.39673
135.33949
129.62604
128.35744
127.10792
127.04115
77.36555
77.10802
76.86002
62.28546
47.75859
28.16687
25.07645
18.44732

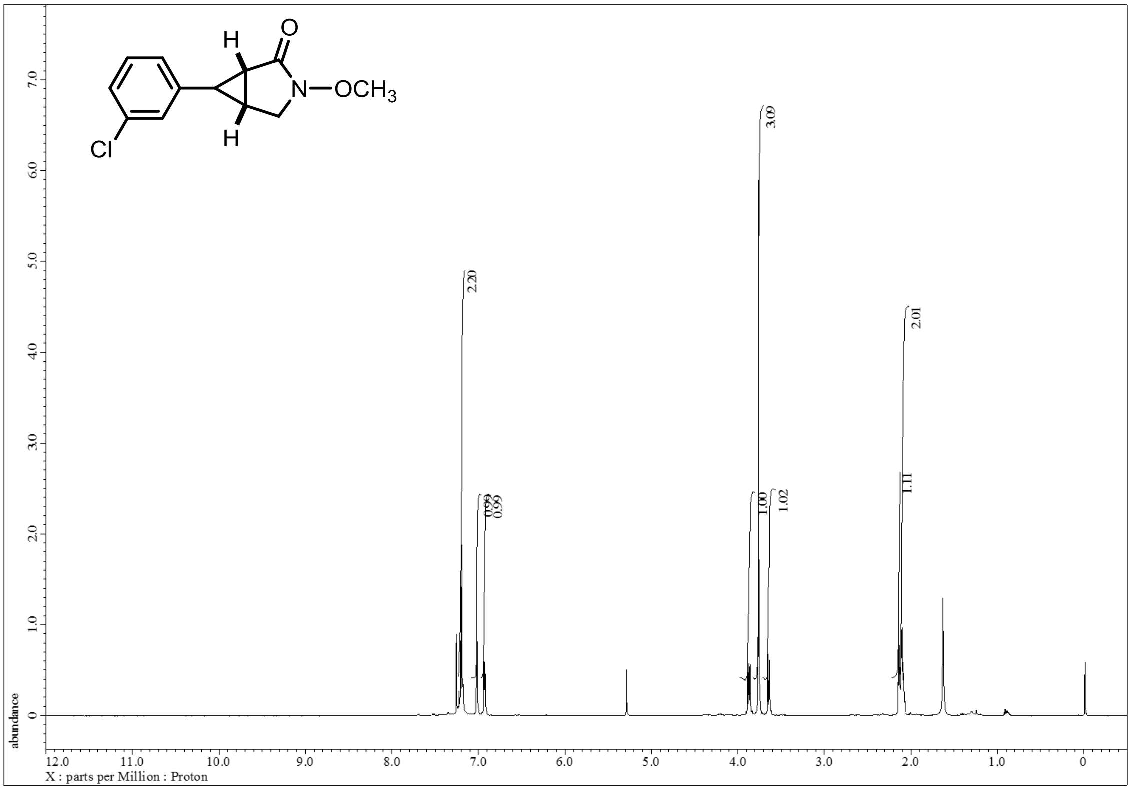
H
O
N—OCH3
H
Cl
abundance
X : parts per Million : Proton
2.20
0.99
0.99
3.09
1.00
1.02
2.01
1.11

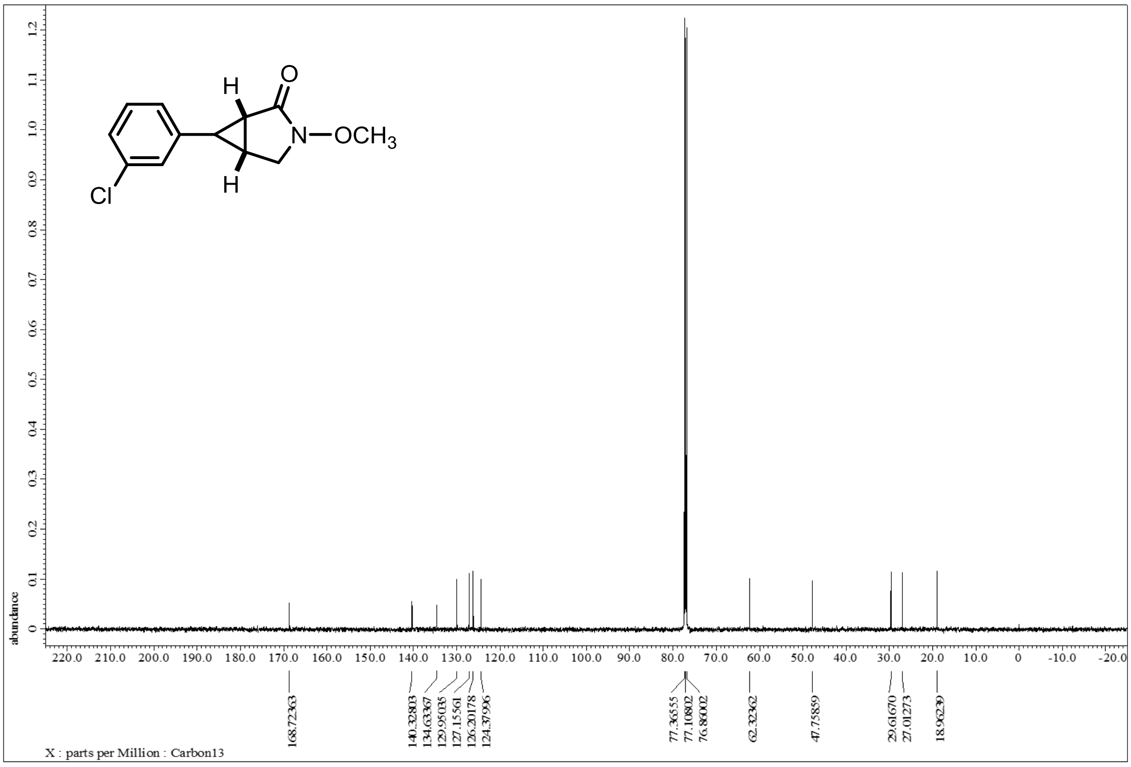
H
O
N—OCH3
H
Cl
abundance
X : parts per Million : Carbon13
168.72363
140.32803
134.63367
129.95035
127.15561
126.20178
124.37996
77.36555
77.10802
76.86002
62.32362
47.75859
29.61670
27.01273
18.96239

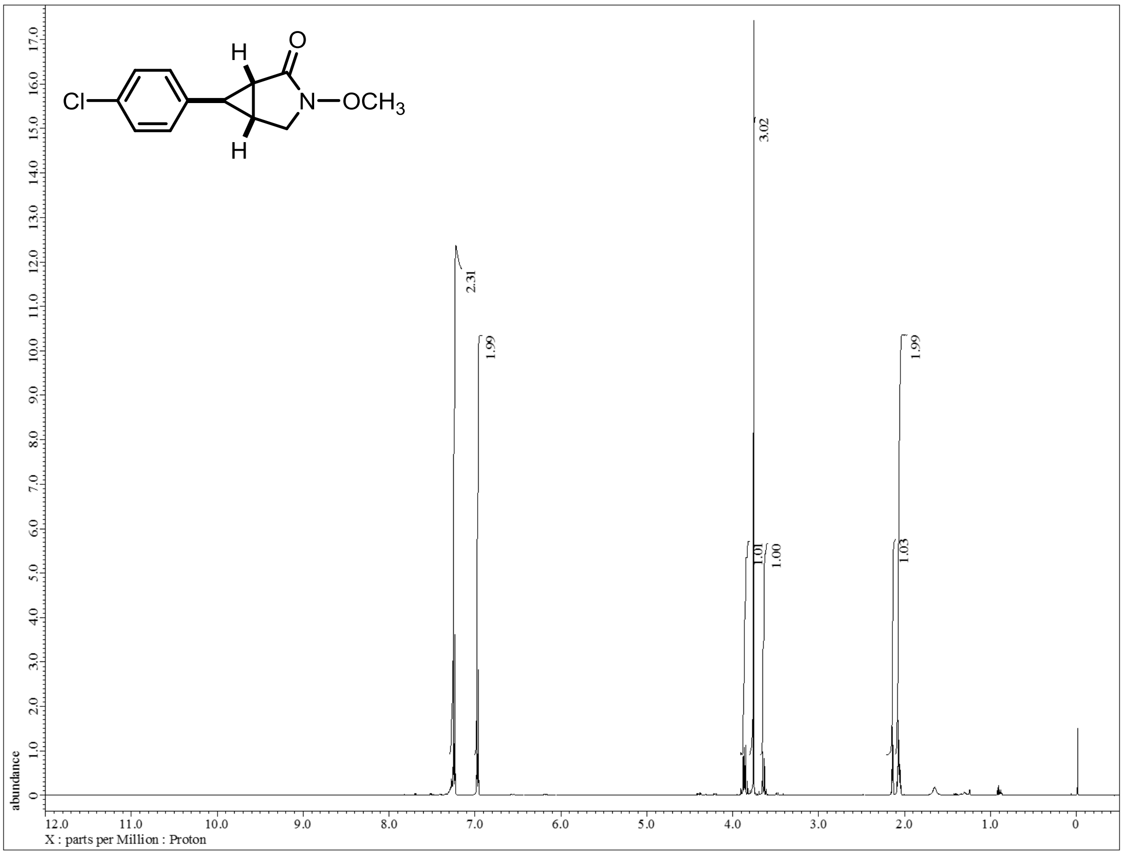
Cl
H
O
N—OCH3
H
abundance
3.02
2.31
1.99
1.01
1.00
1.99
1.03
12.0 11.0 10.0 9.0 8.0 7.0 6.0 5.0 4.0 3.0 2.0 1.0 0
X : parts per Million : Proton

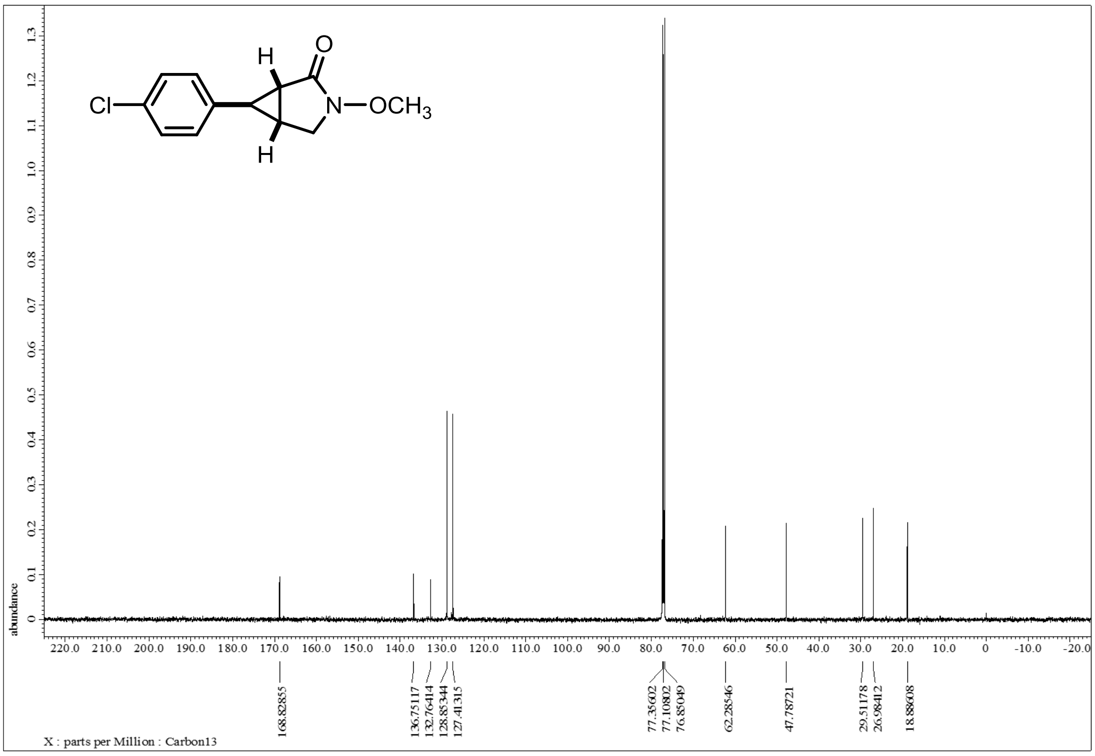
Cl
H
O
N—OCH3
H
abundance
168.82855
136.75117
132.76414
128.85344
127.41315
77.35602
77.10802
76.85049
62.28546
47.78721
29.51178
26.98412
18.88608
220.0 210.0 200.0 190.0 180.0 170.0 160.0 150.0 140.0 130.0 120.0 110.0 100.0 90.0 80.0 70.0 60.0 50.0 40.0 30.0 20.0 10.0 0 -10.0 -20.0
X : parts per Million : Carbon13

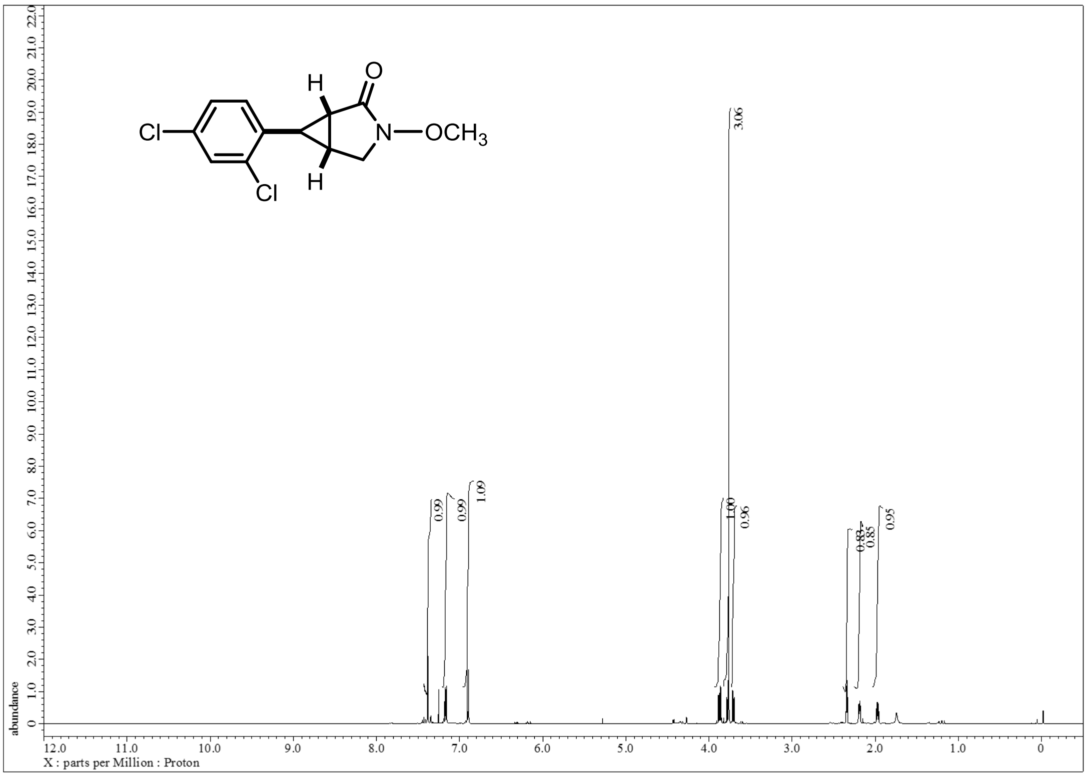

Cl
Cl
H
H
O
N—OCH3
abundance
X : parts per Million : Proton
3.06
0.99
0.99
1.09
1.00
0.96
0.83
0.85
0.95

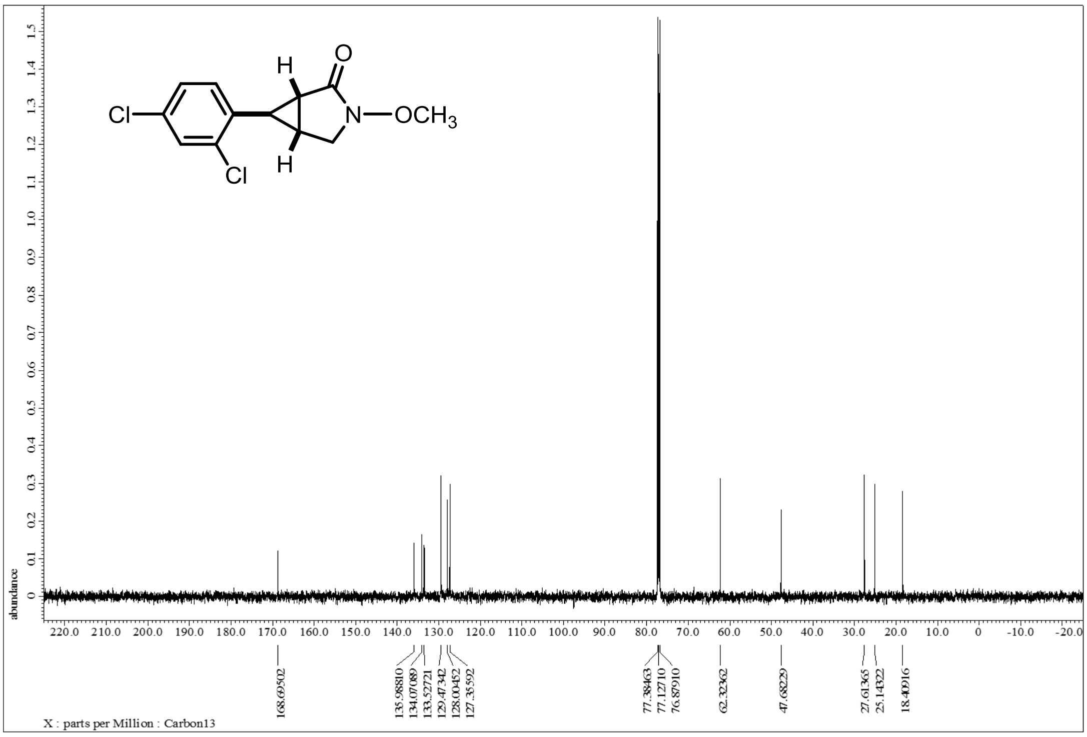

Cl
Cl
H
H
O
N—OCH3
abundance
X : parts per Million : Carbon13
168.69502
135.98810
134.07089
133.52721
129.47342
128.00452
127.35592
77.38463
77.12710
76.87910
62.32362
47.68229
27.61365
25.14322
18.40916

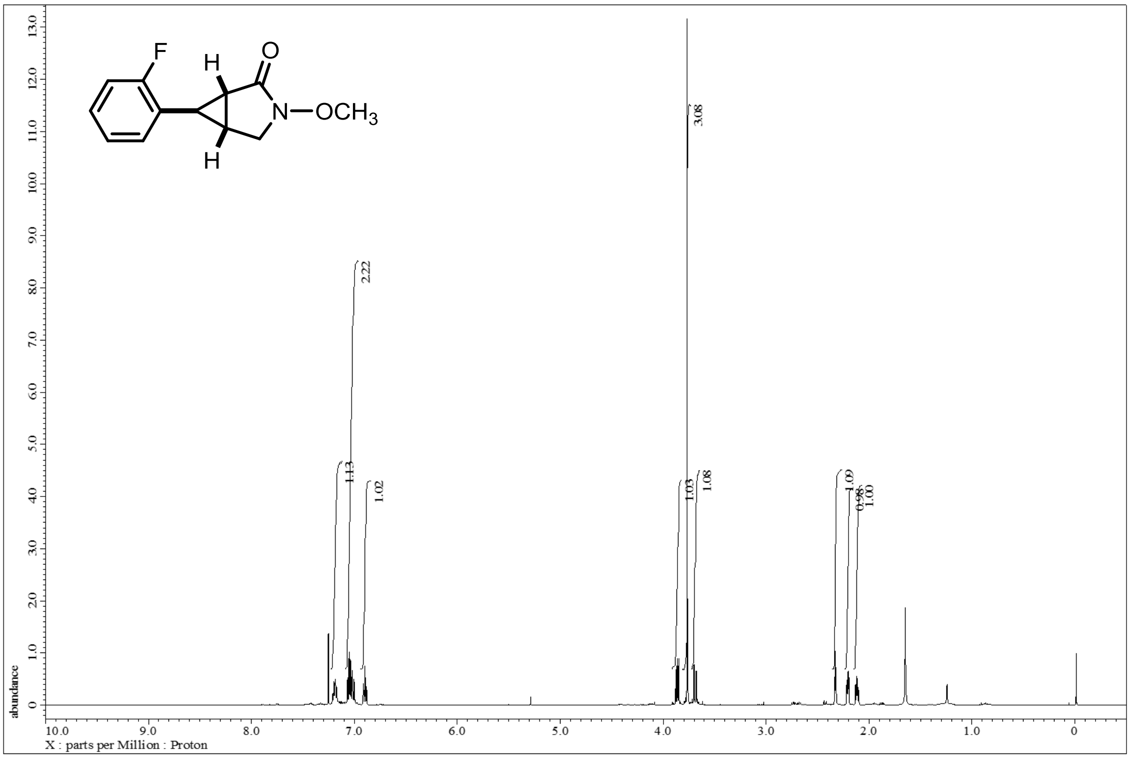
F
H
O
N—OCH3
H
X : parts per Million : Proton
abundance

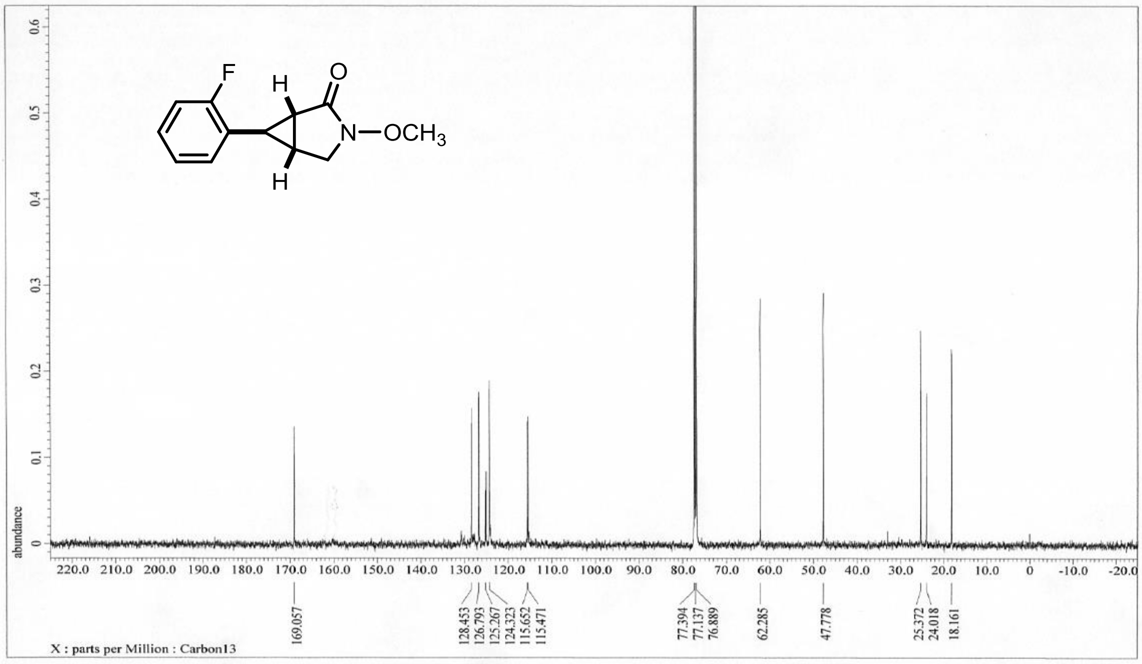
F
H
O
N—OCH3
H
169.057
128.453
126.793
125.267
124.323
115.652
115.471
77.394
77.137
76.889
62.285
47.778
25.372
24.018
18.161
X : parts per Million : Carbon13
abundance

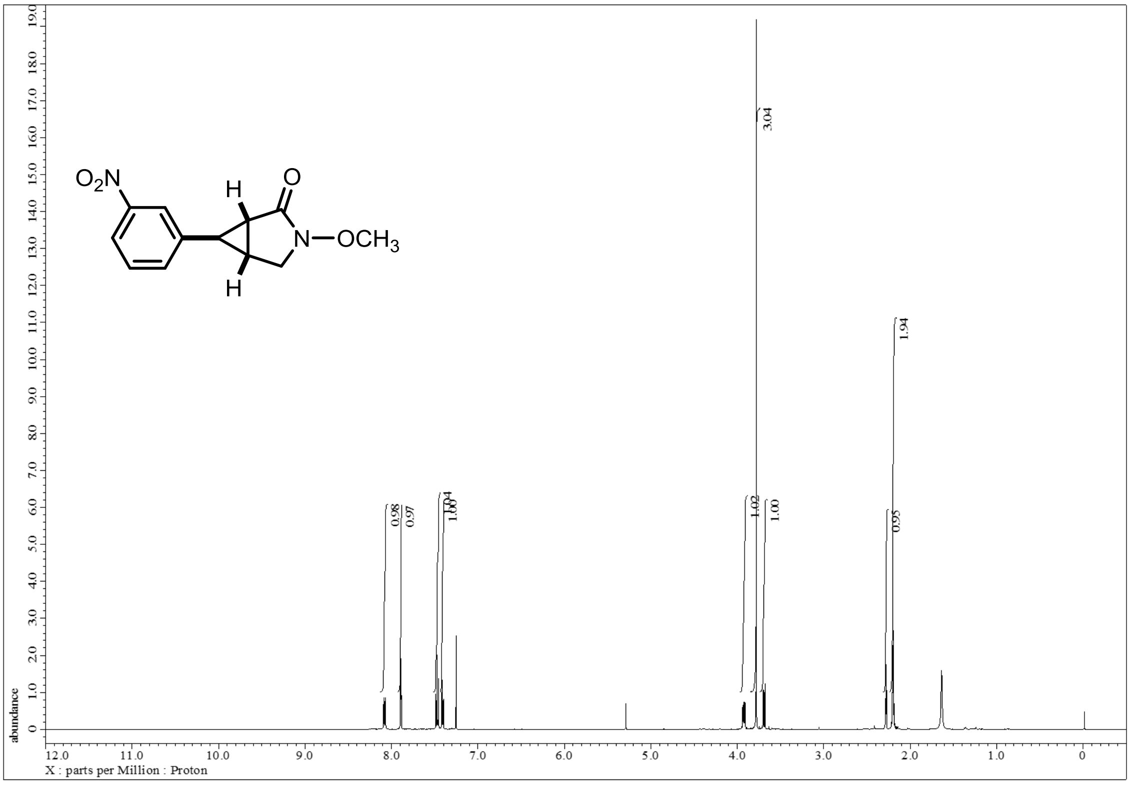
O2N
H
O
N—OCH3
H
3.04
1.94
0.98
0.97
1.04
1.00
1.02
1.00
0.95
abundance
X : parts per Million : Proton

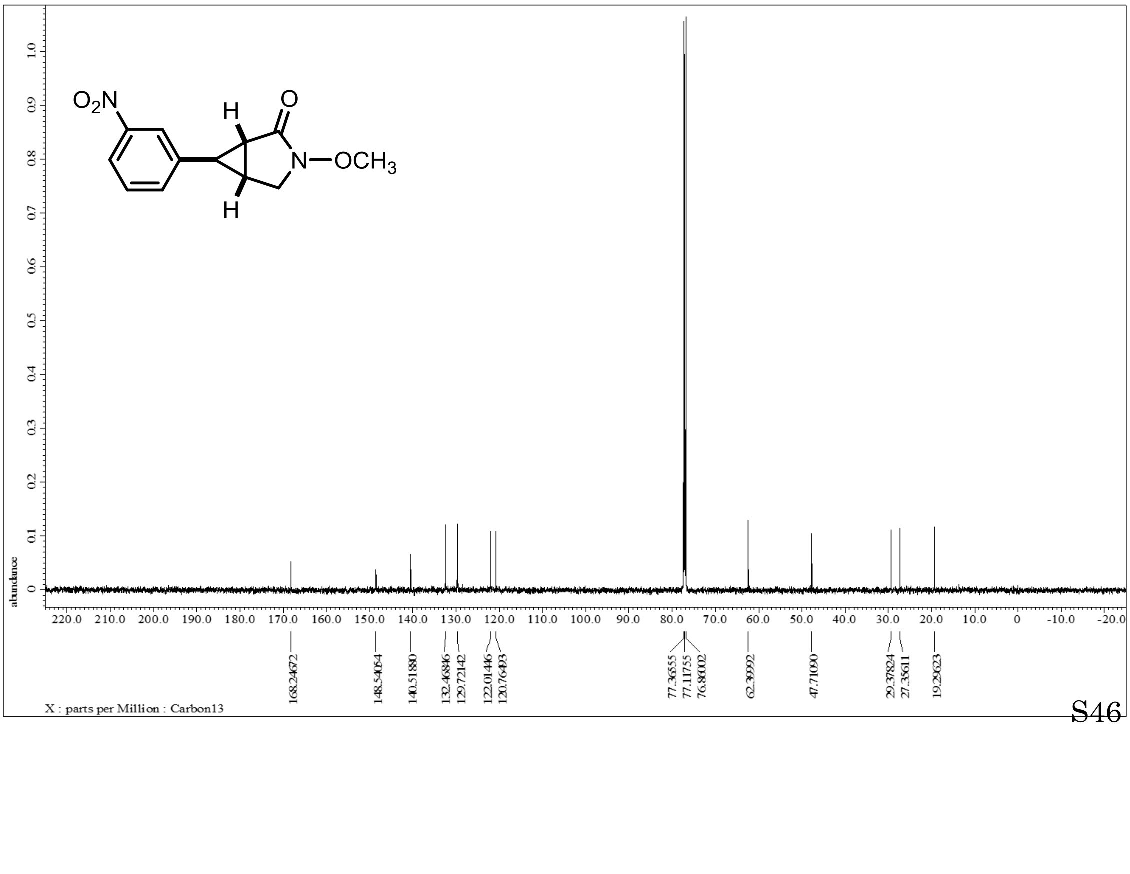
O2N
H
O
N—OCH3
H
abundance
168.24672
148.54054
140.51880
132.46846
129.72142
122.01446
120.76493
77.36555
77.11755
76.86002
62.39992
47.71090
29.37824
27.35611
19.29623
X : parts per Million : Carbon13

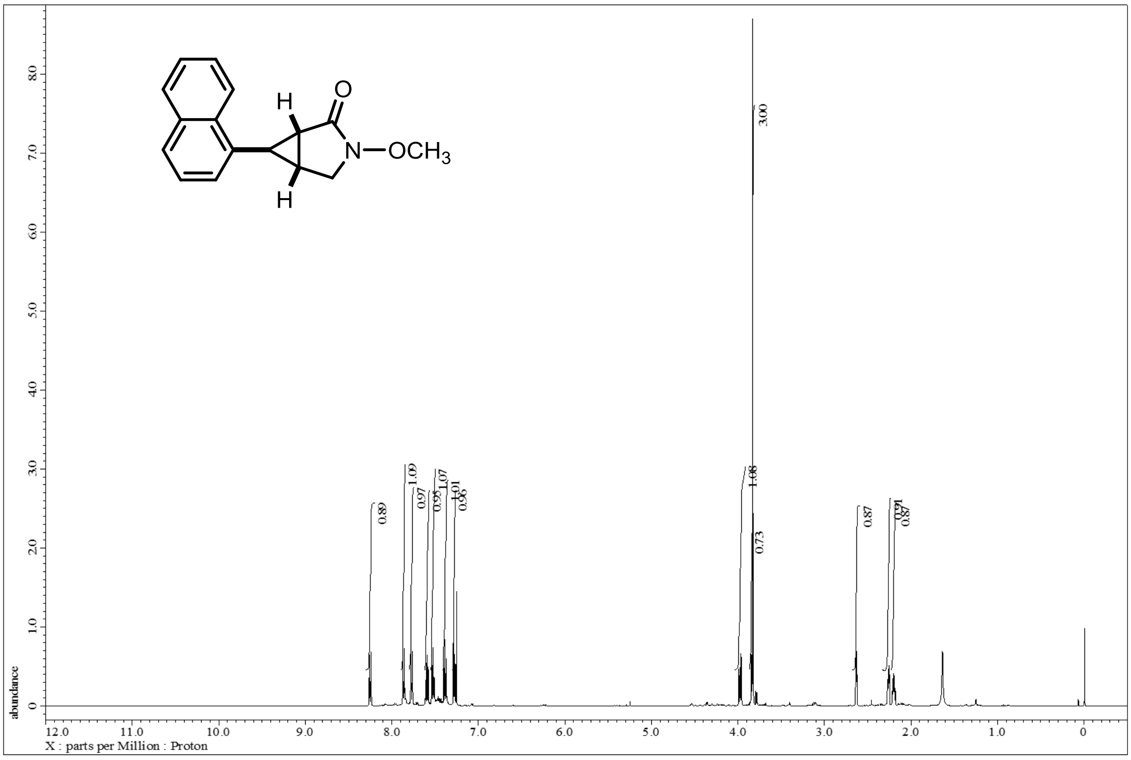

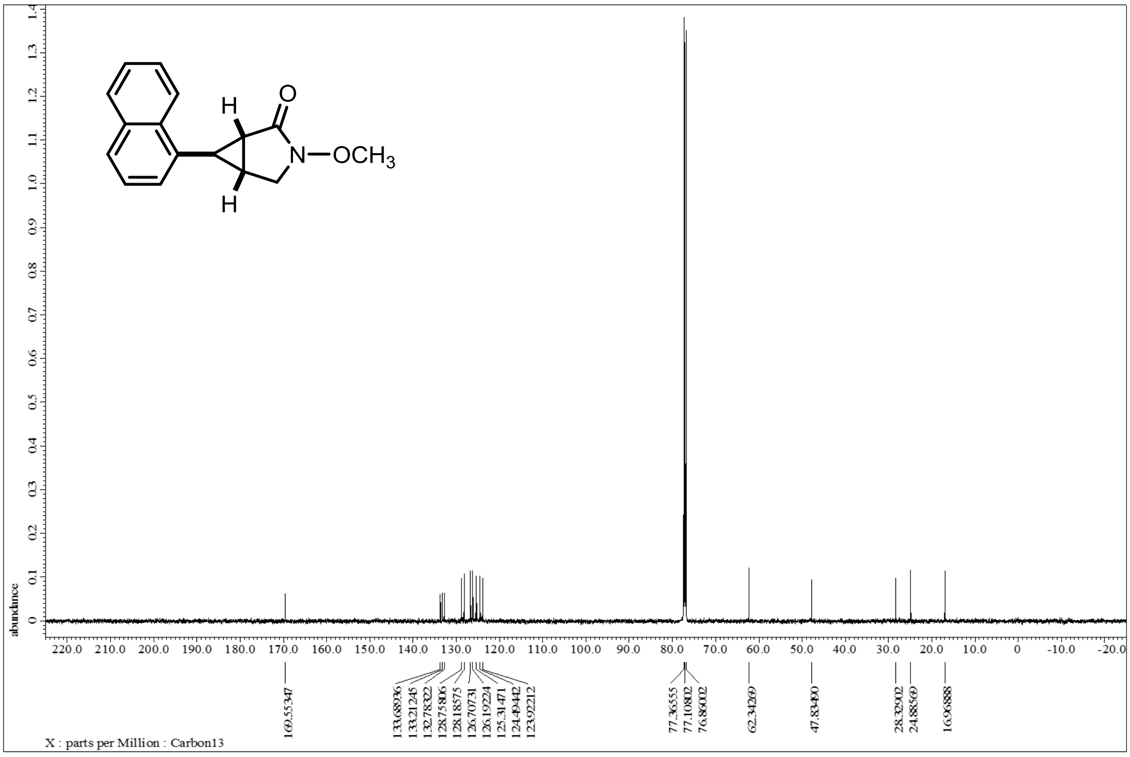

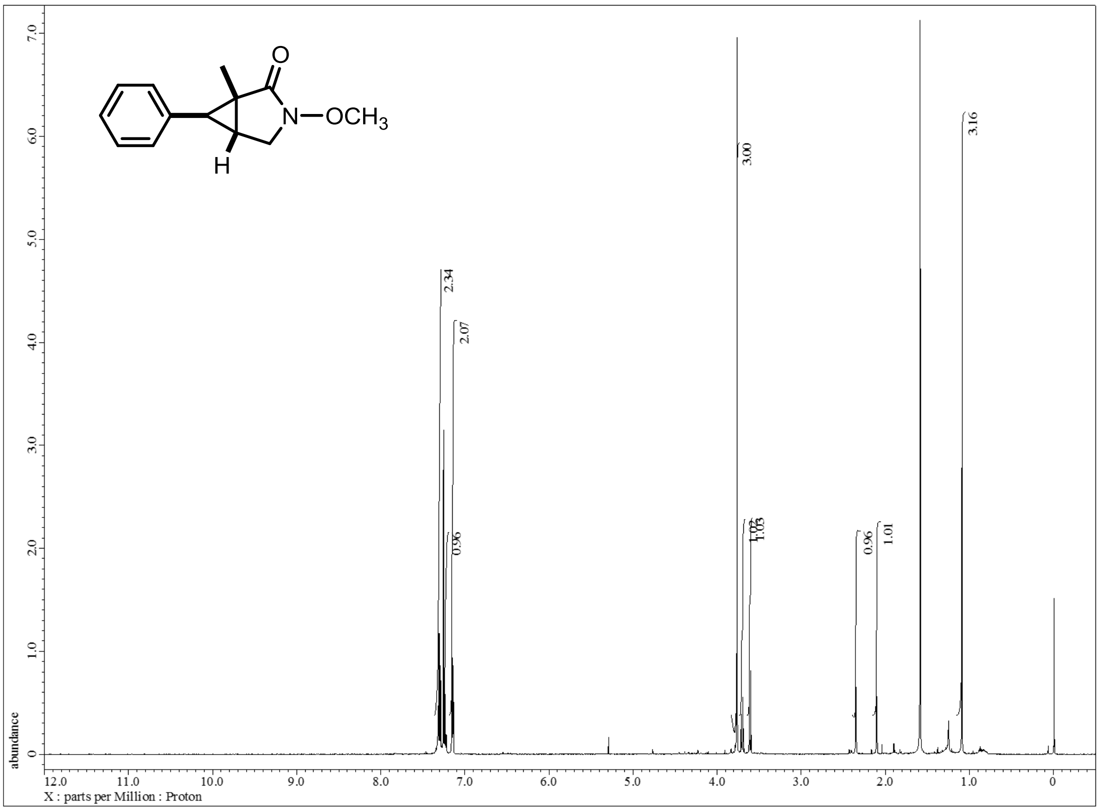

N—OCH3
O
H
3.00
2.34
2.07
0.96
1.02
1.03
0.96
1.01
3.16
abundance
X : parts per Million : Proton

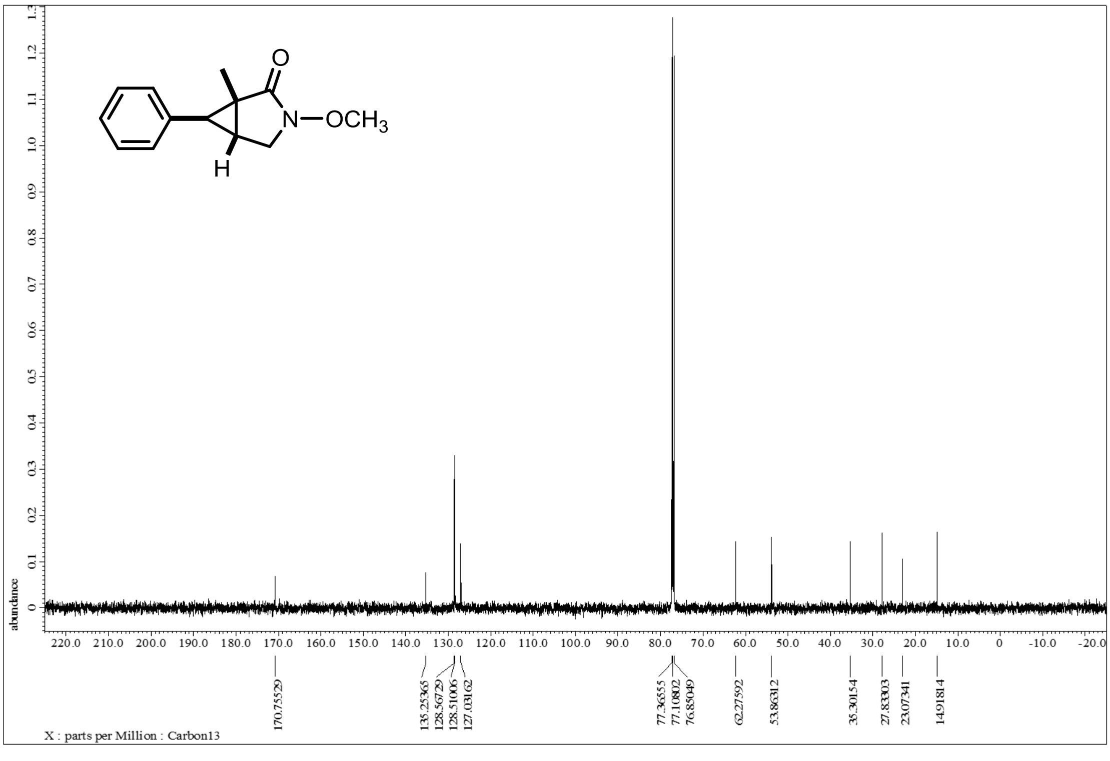

N—OCH3
O
H
abundance
170.75529
135.25365
128.56729
128.51006
127.03162
77.36555
77.10802
76.85049
62.27592
53.86312
35.30154
27.83303
23.07341
14.91814
X : parts per Million : Carbon13

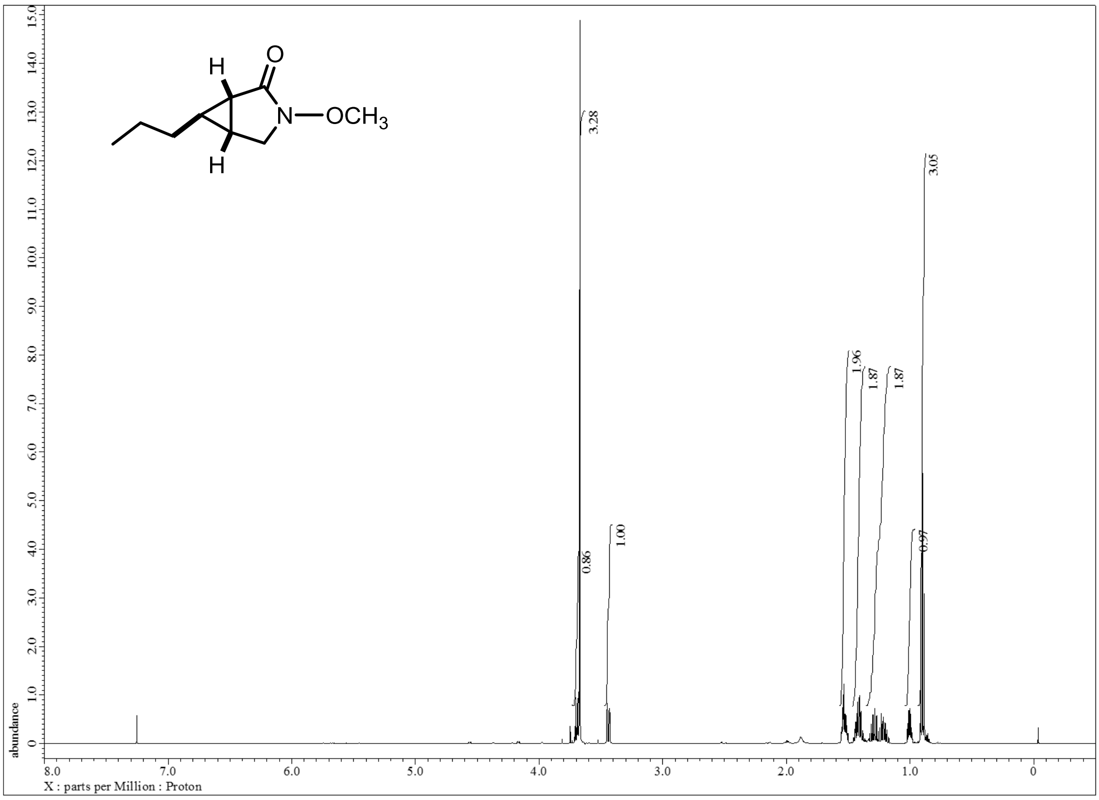

3.28
3.05
1.96
1.87
1.87
0.86
1.00
0.97
abundance
X : parts per Million : Proton

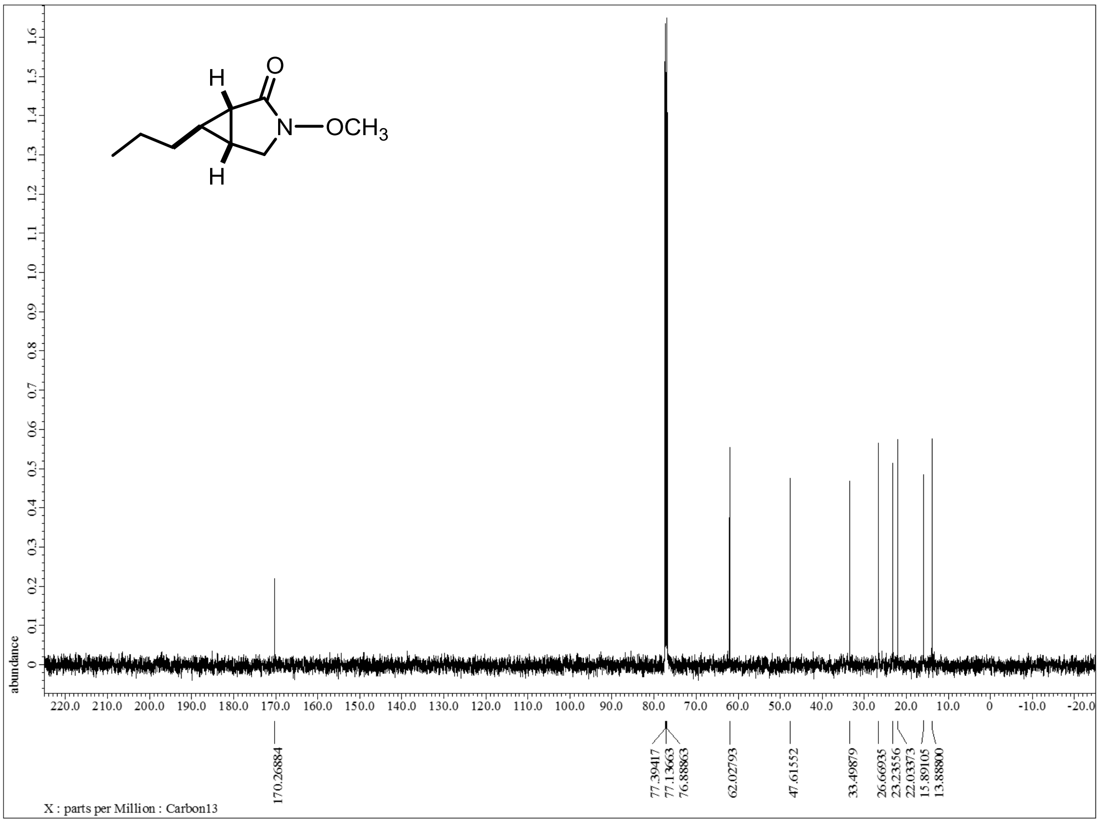

170.26884
77.39417
77.13663
76.88863
62.02793
47.61552
33.49879
26.66935
23.23556
22.03373
15.89105
13.88800
abundance
X : parts per Million : Carbon13

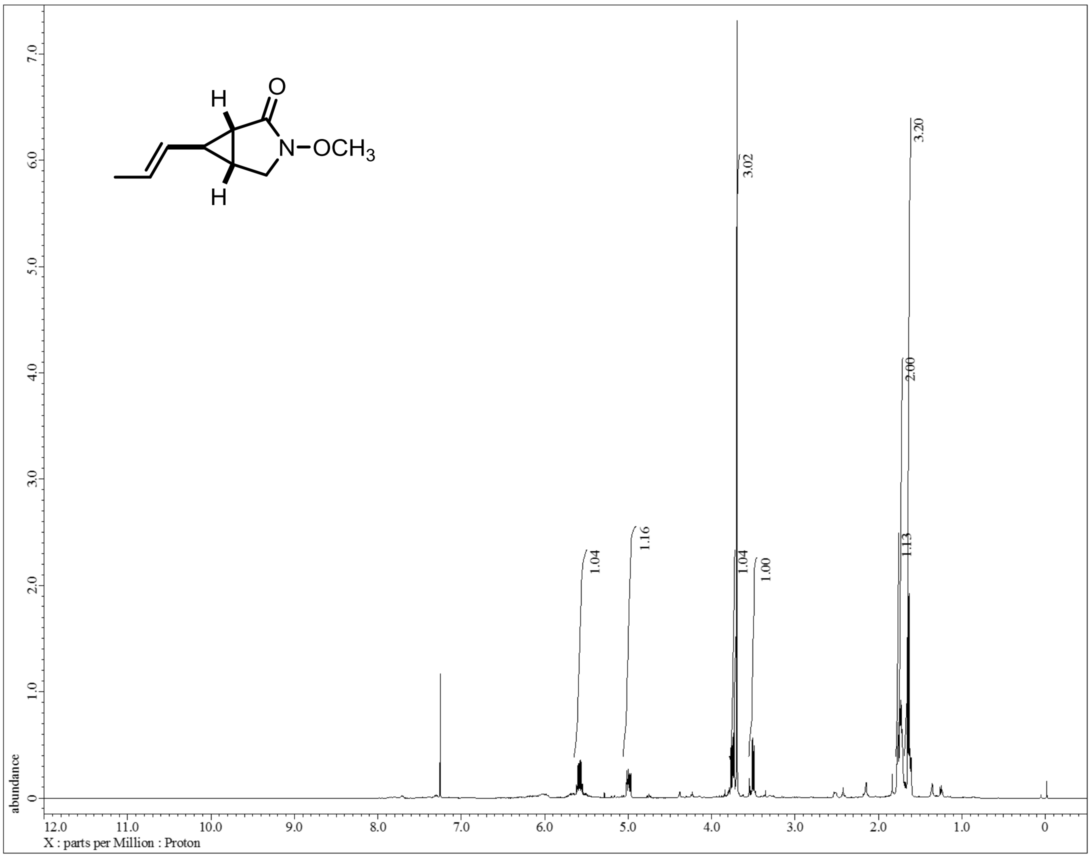
H
O
N−OCH3
H
7.0
6.0
5.0
4.0
3.0
2.0
1.0
0
abundance
3.02
3.20
2.00
1.04
1.16
1.04
1.00
1.13
12.0
11.0
10.0
9.0
8.0
7.0
6.0
5.0
4.0
3.0
2.0
1.0
0
X : parts per Million : Proton

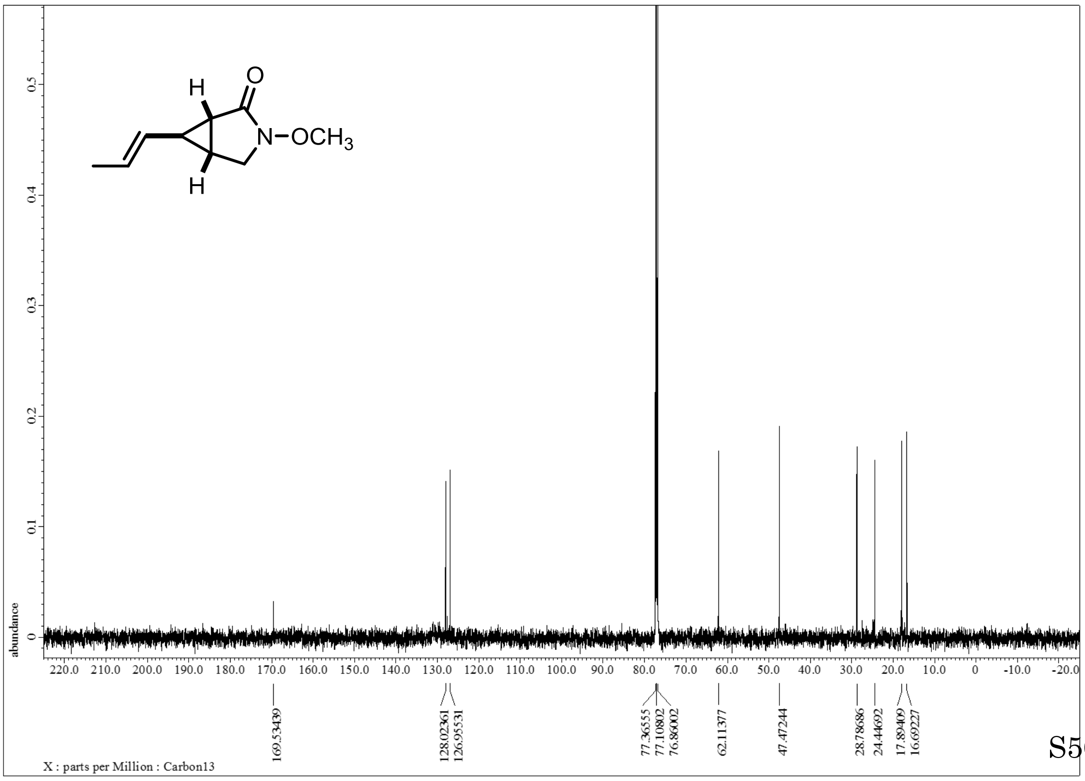
H
O
N−OCH3
H
0.5
0.4
0.3
0.2
0.1
0
abundance
220.0
210.0
200.0
190.0
180.0
170.0
160.0
150.0
140.0
130.0
120.0
110.0
100.0
90.0
80.0
70.0
60.0
50.0
40.0
30.0
20.0
10.0
0
-10.0
-20.0
169.53439
128.02361
126.95531
77.36555
77.10802
76.86002
62.11377
47.47244
28.78686
24.44692
17.89409
16.69227
X : parts per Million : Carbon13

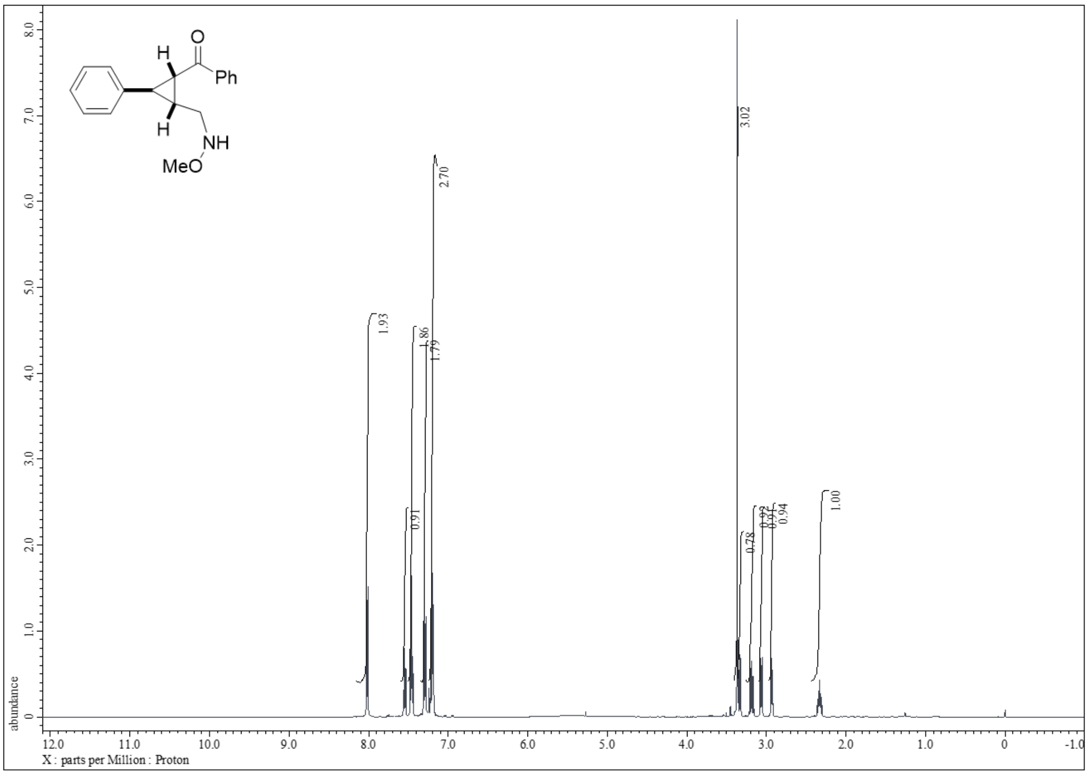
Ph
NH
MeO
abundance
X : parts per Million : Proton
3.02
2.70
1.93
1.86
1.79
0.91
0.78
0.94
1.00

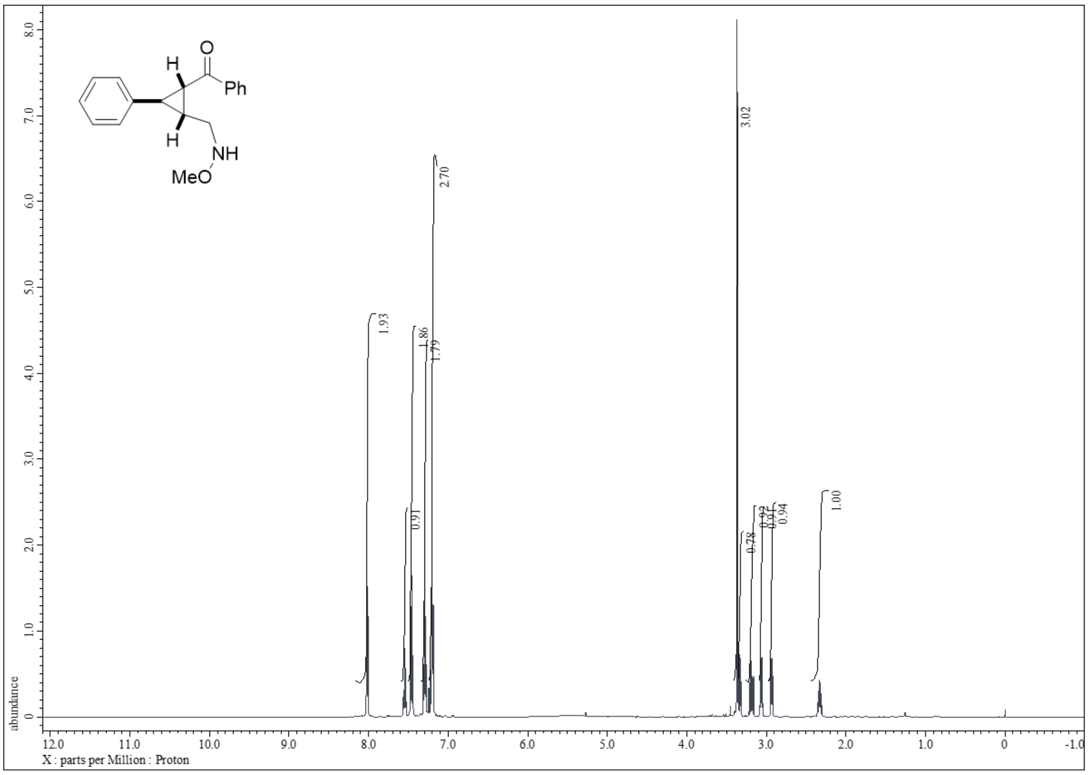
Ph
NH
MeO
abundance
X : parts per Million : Proton
3.02
2.70
1.93
1.86
1.79
0.91
0.78
0.94
1.00

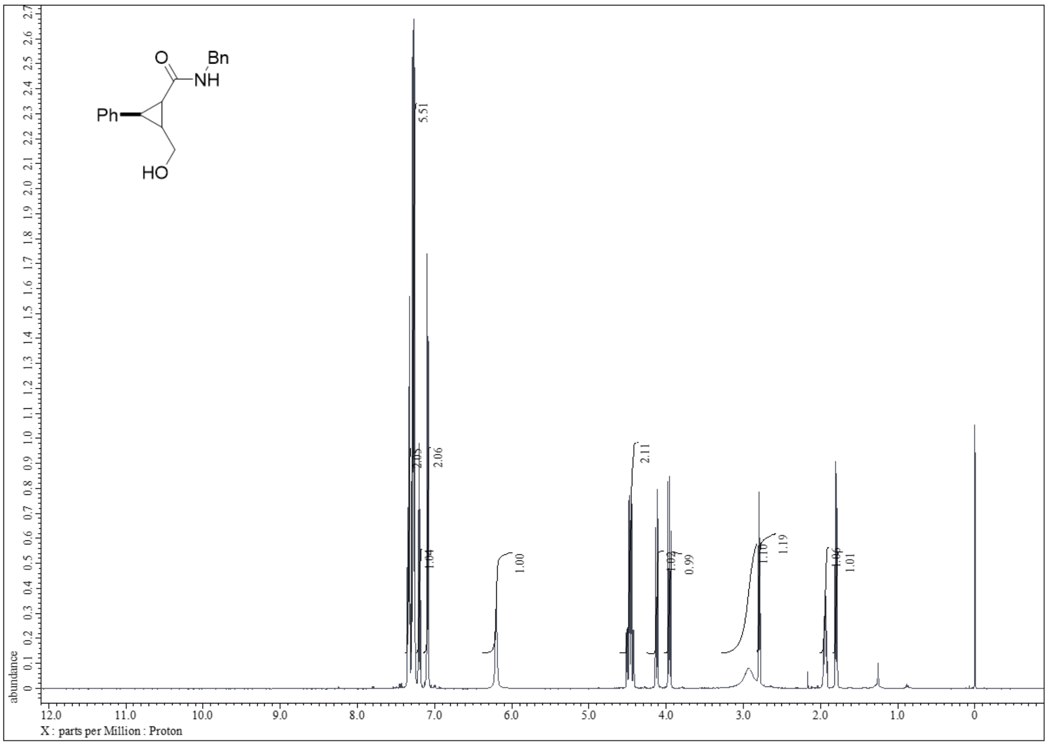
O
Bn
NH
Ph
HO
abundance
X : parts per Million : Proton
5.51
2.03
2.06
1.04
1.00
2.11
1.02
0.99
1.10
1.19
1.04
1.01

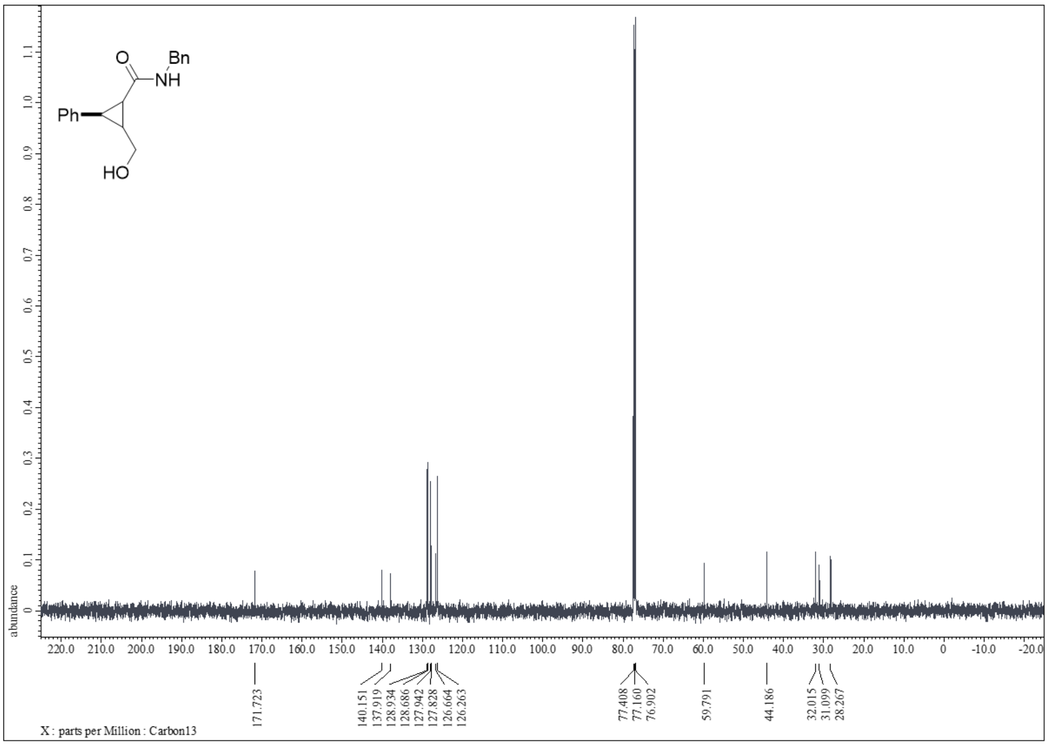
O
Bn
NH
Ph
HO
abundance
X : parts per Million : Carbon13
171.723
140.151
137.919
128.934
128.686
127.942
127.828
126.664
126.263
77.408
77.160
76.902
59.791
44.186
32.015
31.099
28.267

11. HPLC spectral data

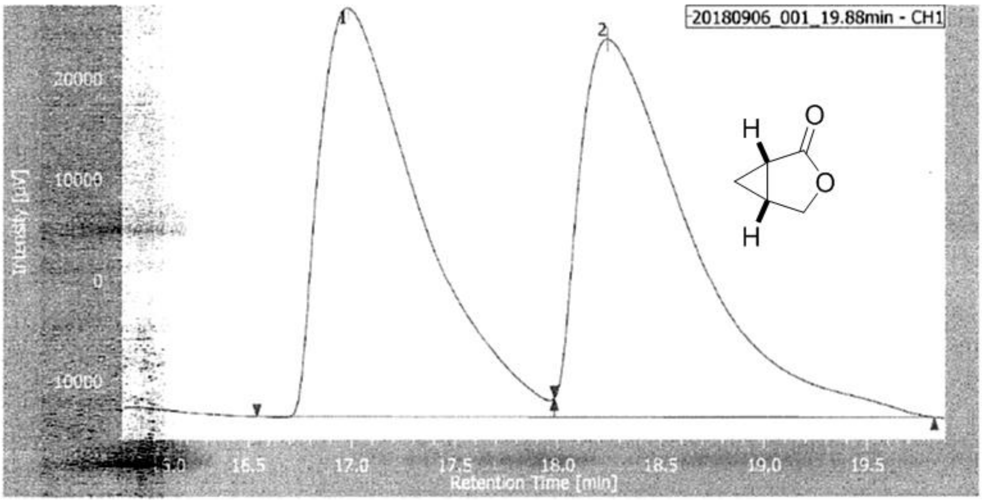

entry	RT [min]	area	area %
1	16.9	1370311	49.23
2	18.2	1413078	50.768

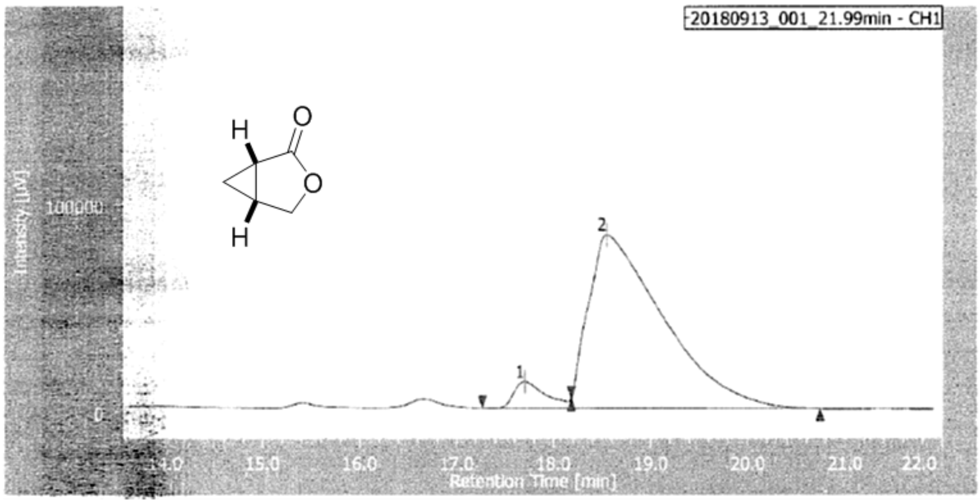

entry	RT [min]	area	area %
1	17.7	296341	6.18
2	18.5	4494087	93.81

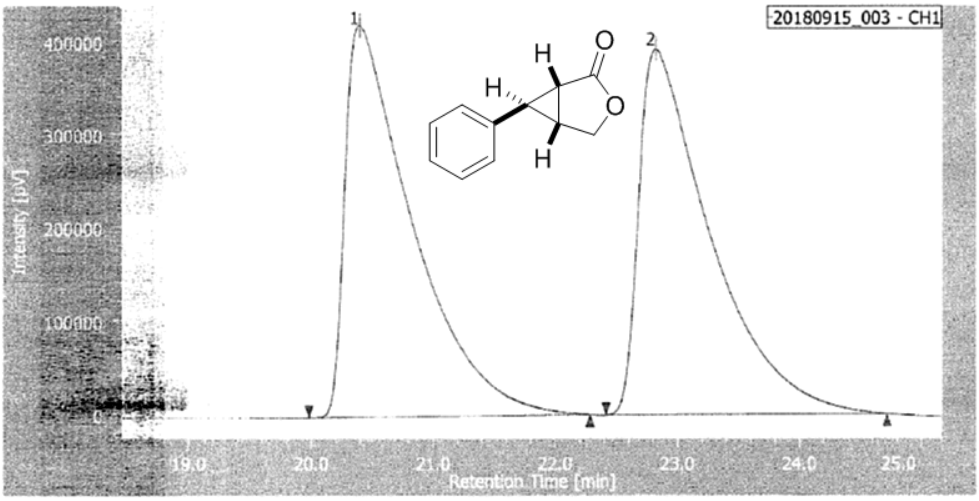

PEAK	RT [min]	area	area %
1	20.4	16209356	50.38
2	22.8	15964206	49.619

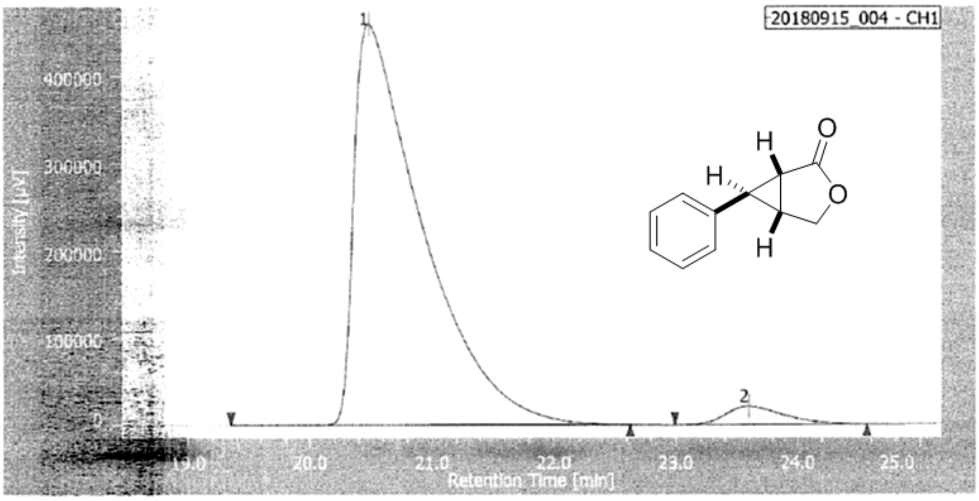

entry	RT [min]	area	area %
1	20.4	17907788	96.34
2	23.6	679258	3.654

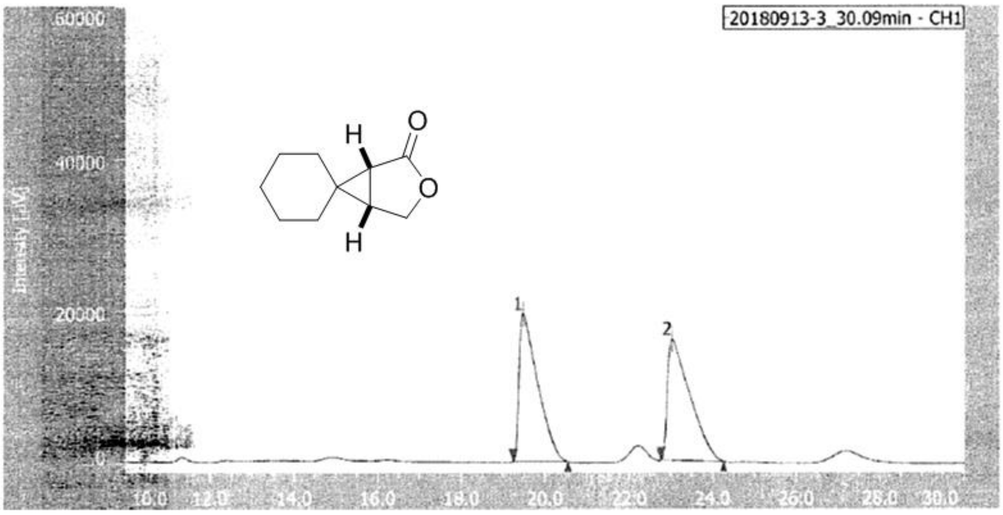

entry	RT [min]	area	area %
1	19.4	597989	50.42
2	23.0	587829	49.57

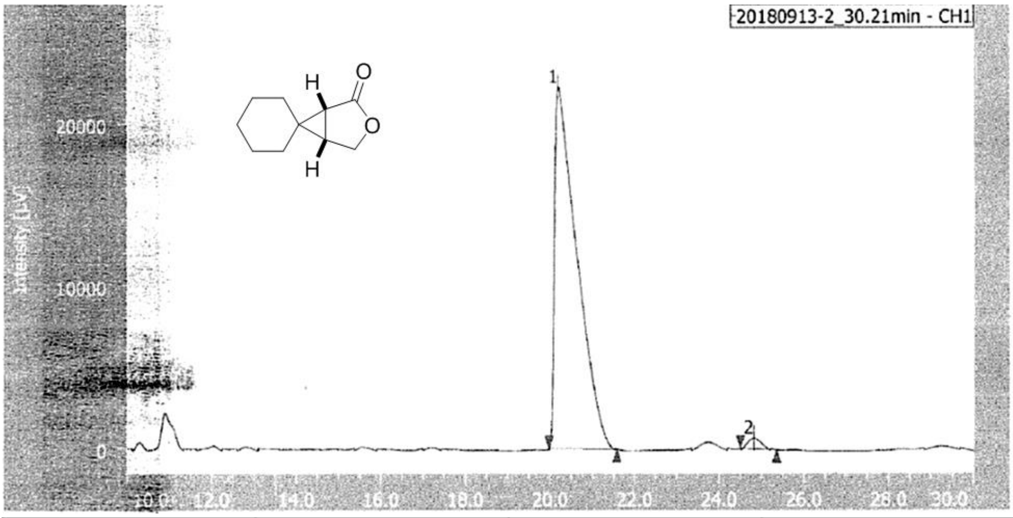

entry	RT [min]	area	area %
1	20.2	815328	98.33
2	24.8	13819	1.66

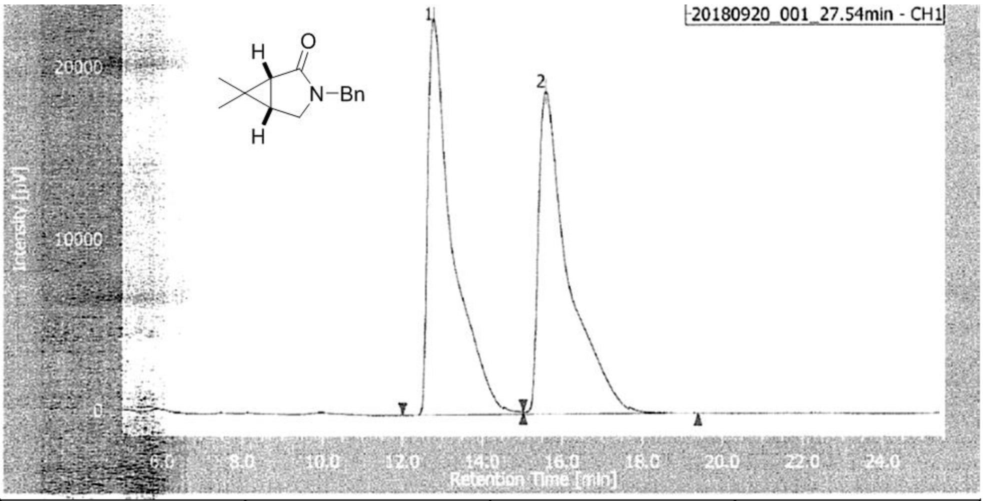

entry	RT [min]	area	area %
1	12.7	996097	50.14
2	15.6	990498	49.85

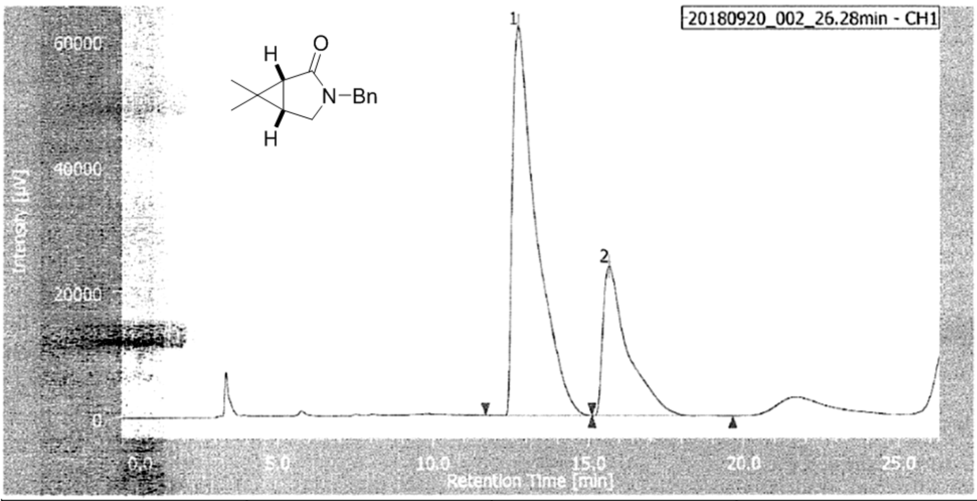

entry	RT [min]	area	area %
1	12.7	3332812	71.68
2	15.6	1316145	28.31

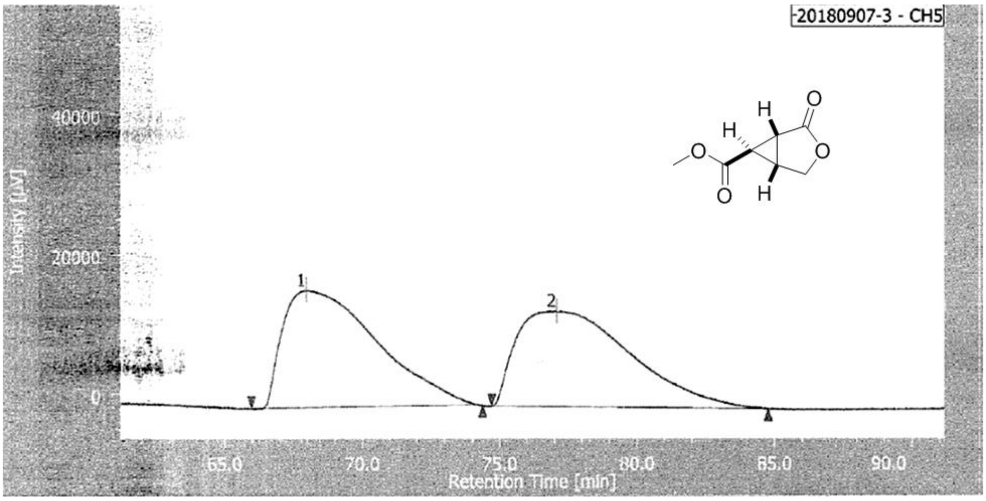

entry	RT [min]	area	area %
1	67.9	4019087	50.34
2	77.0	3963716	49.65

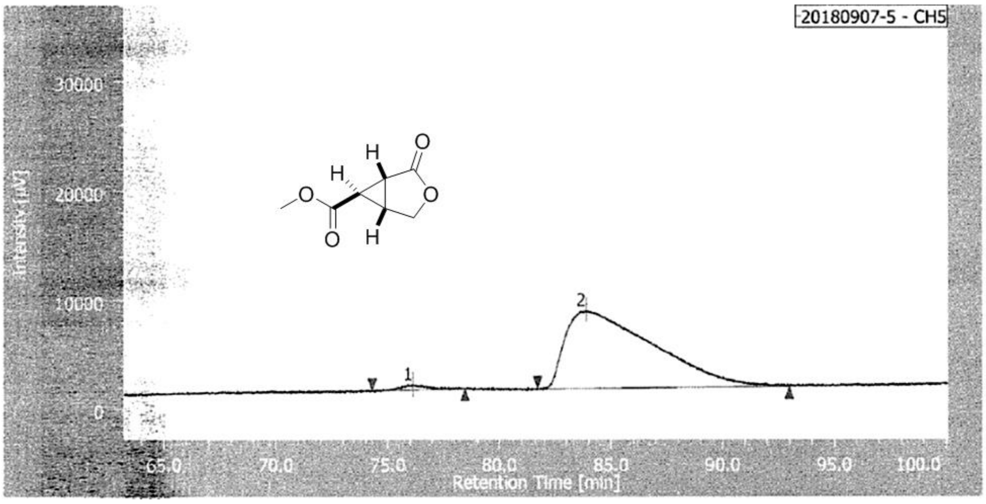

entry	RT [min]	area	area %
1	76.1	39197	1.98
2	83.8	1940744	98.02

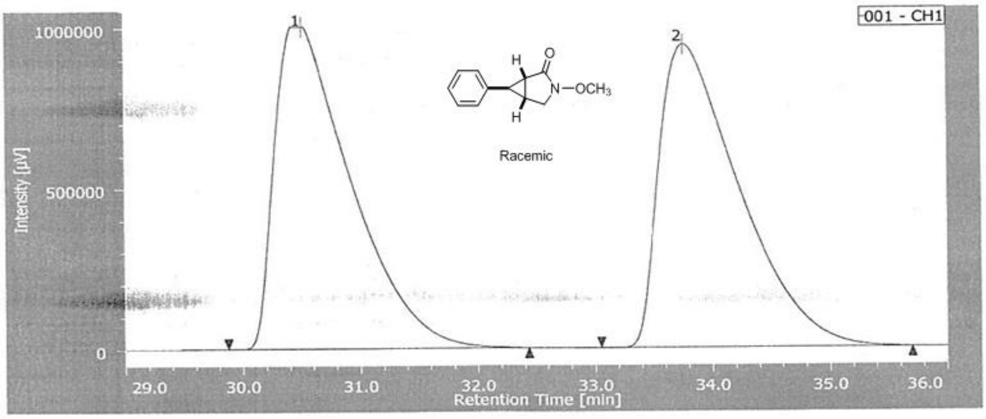

entry	tR[min]	area	area %
1	30.50	45256405	50.47
2	33.75	44404766	49.53

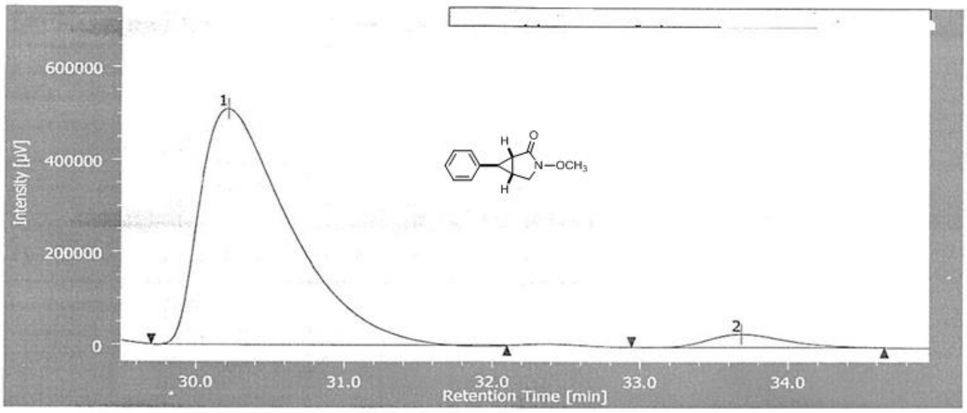

entry	tR[min]	area	area %
1	30.21	21412045	95.34
2	33.68	1044654	4.65

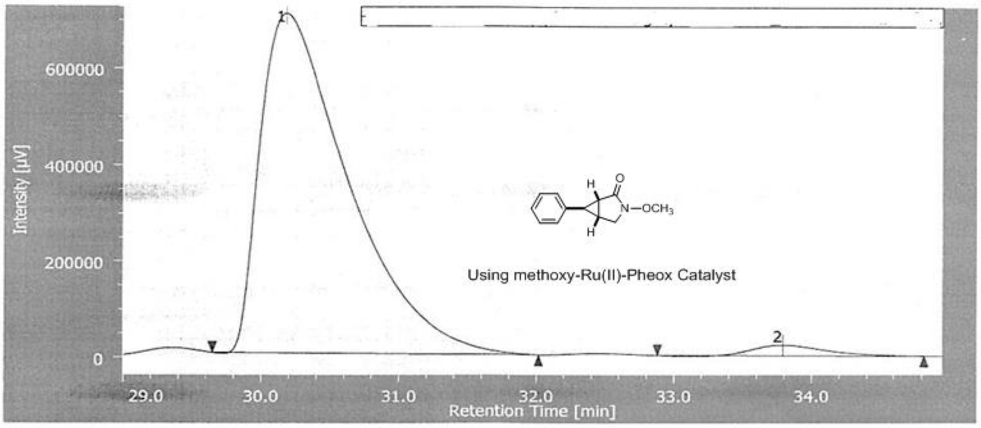

entry	tR[min]	area	area %
1	30.17	31355247	97.52
2	33.79	796566	2.48

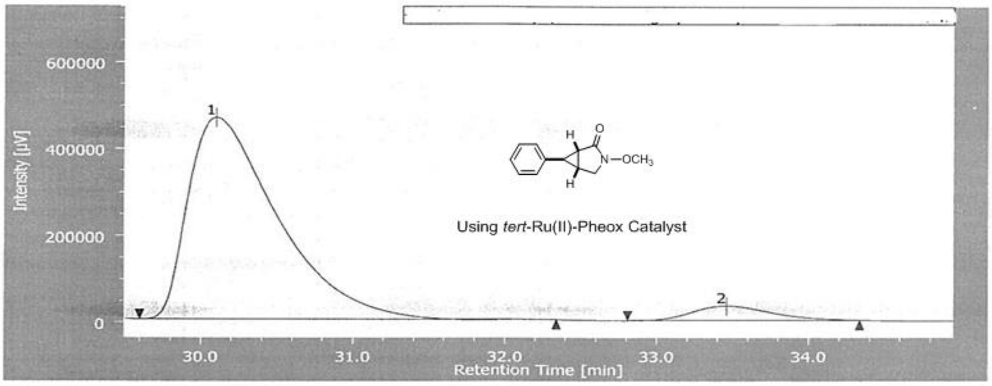

entry	tR[min]	area	area %
1	30.10	19008278	93.99
2	33.46	1215067	6.01

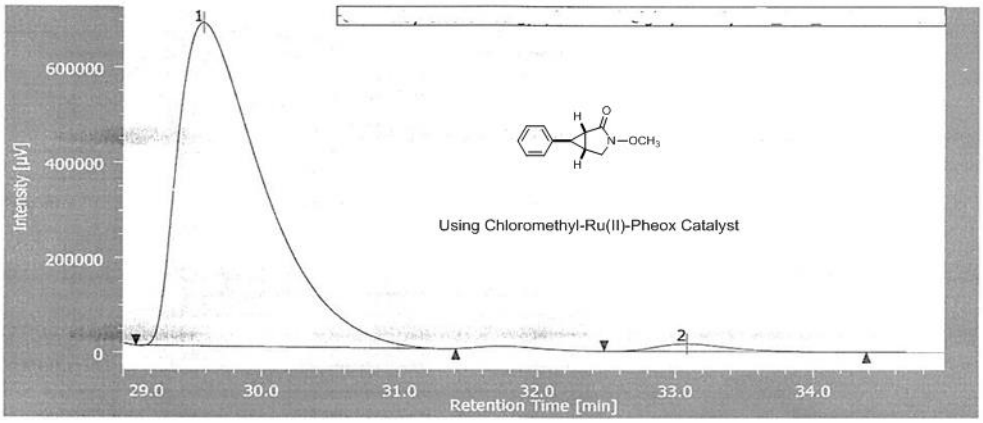

entry	tR[min]	area	area %
1	29.57	29252529	98.05
2	33.08	581720	1.95

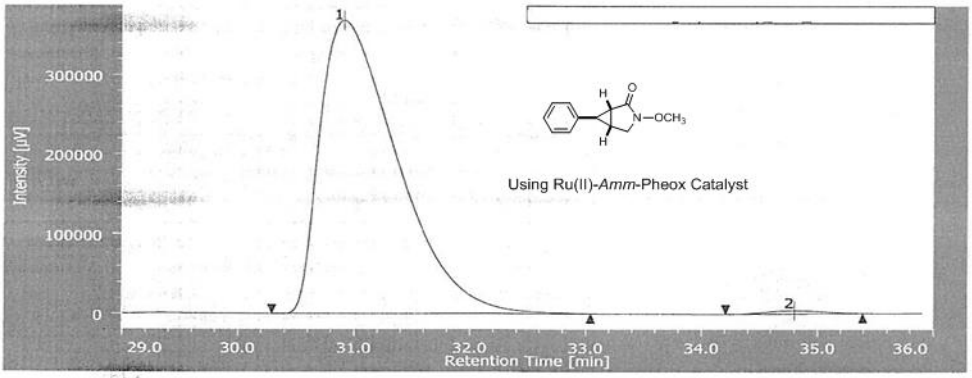

entry	tR[min]	area	area %
1	30.90	16956096	99.16
2	34.80	142997	0.84

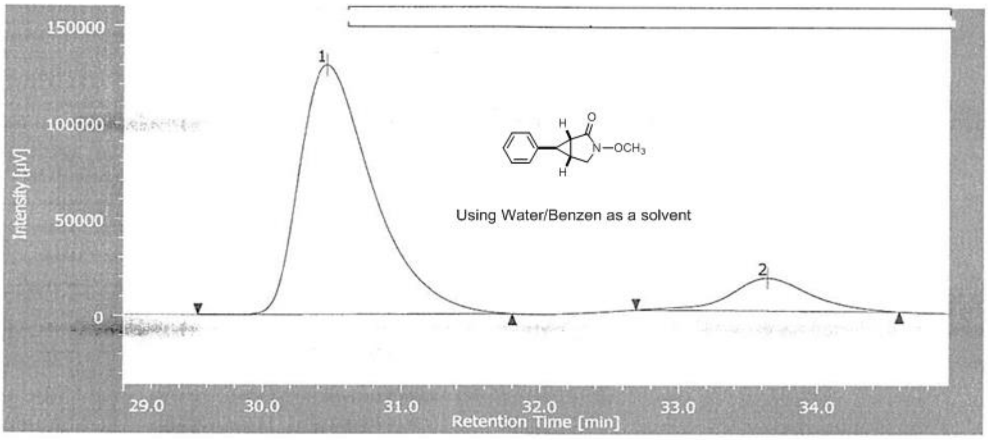

entry	tR[min]	area	area %
1	30.45	4737061	87.43
2	33.64	680996	12.57

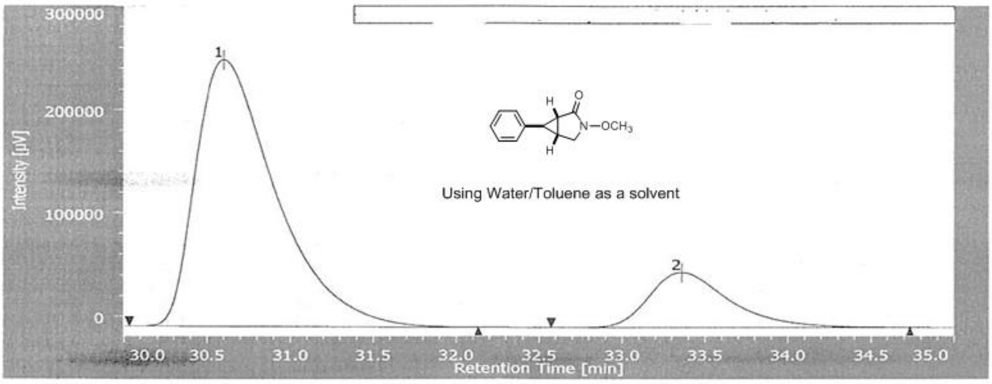

entry	tR[min]	area	area %
1	30.60	8643353	82.73
2	33.35	1803675	17.27

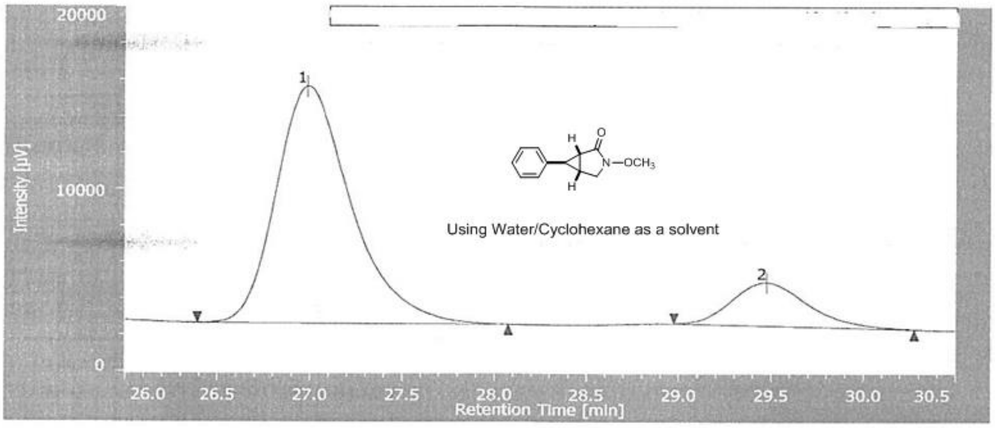

entry	tR[min]	area	area %
1	26.98	367747	84.38
2	29.47	68039	15.62

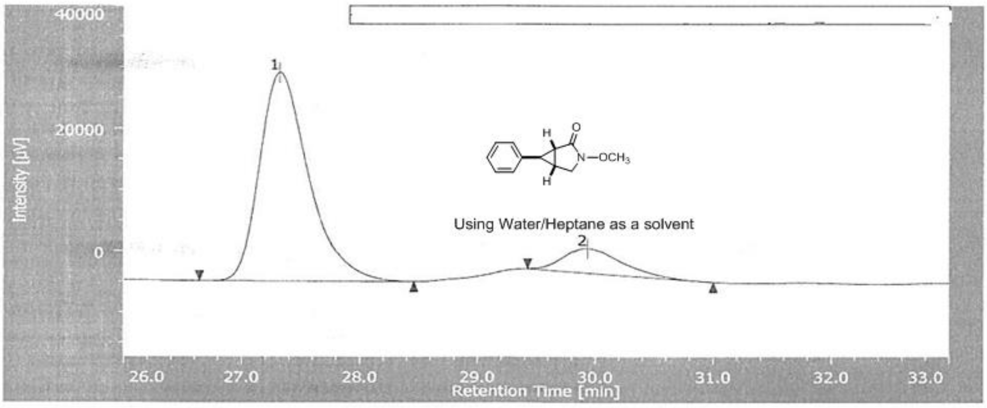

entry	tR[min]	area	area %
1	27.31	985736	87.57
2	29.93	139961	12.43

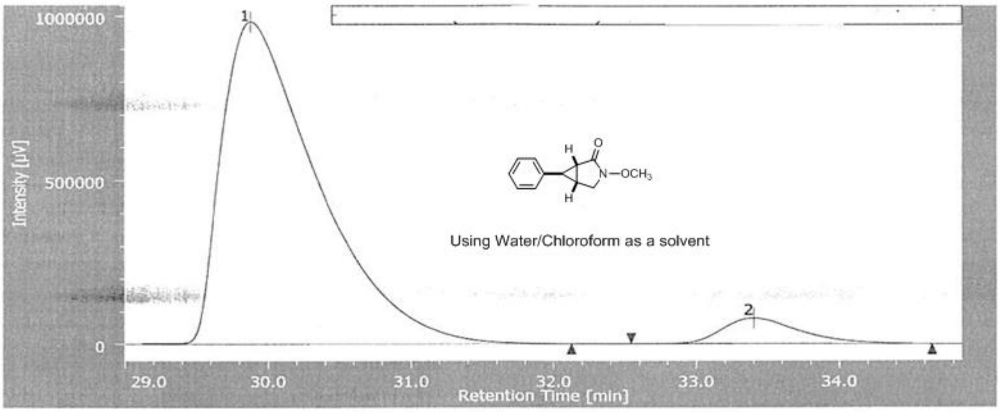

entry	tR[min]	area	area %
1	29.87	45848193	93.925
2	33.40	2965173	6.075

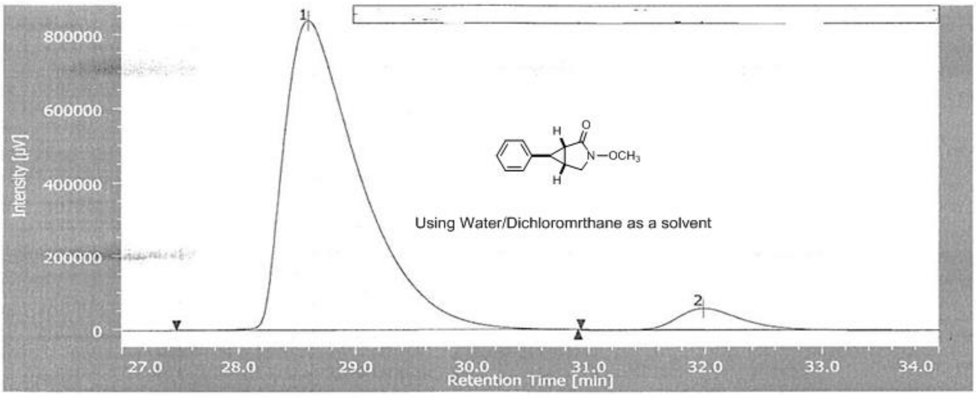

entry	tR[min]	area	area %
1	28.59	38042337	94.68
2	31.98	2137036	5.31

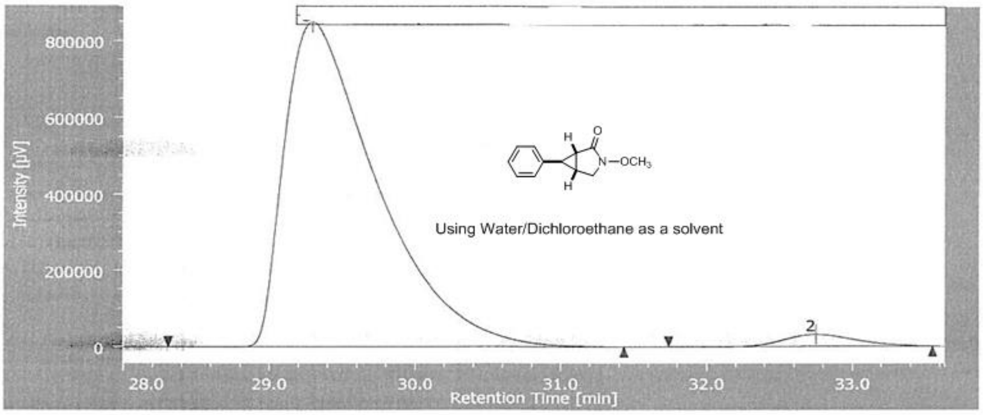

entry	tR[min]	area	area %
1	29.29	37492806	97.21
2	32.75	1075112	2.79

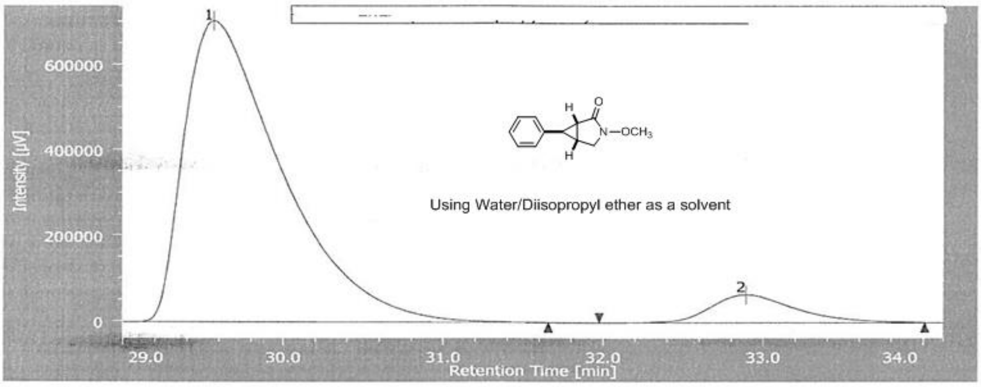

entry	tR[min]	area	area %
1	29.56	29761003	92.53
2	32.89	2401935	7.47

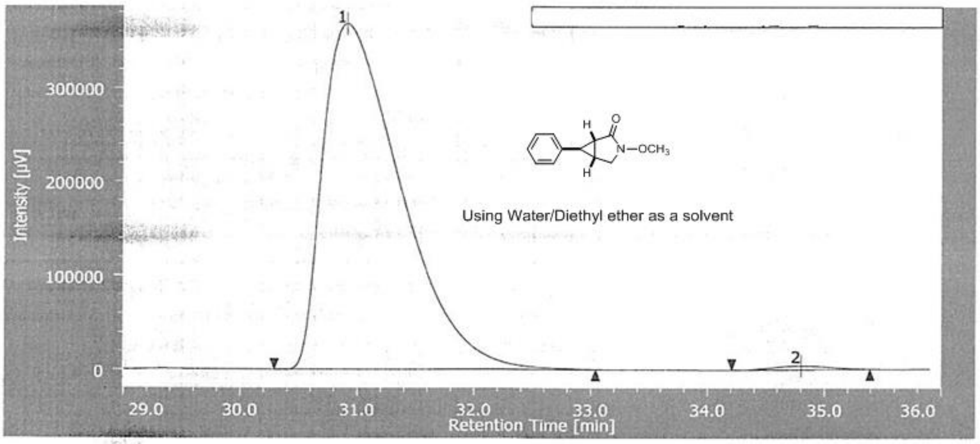

entry	tR[min]	area	area %
1	30.90	16956096	99.17
2	34.80	142997	0.83

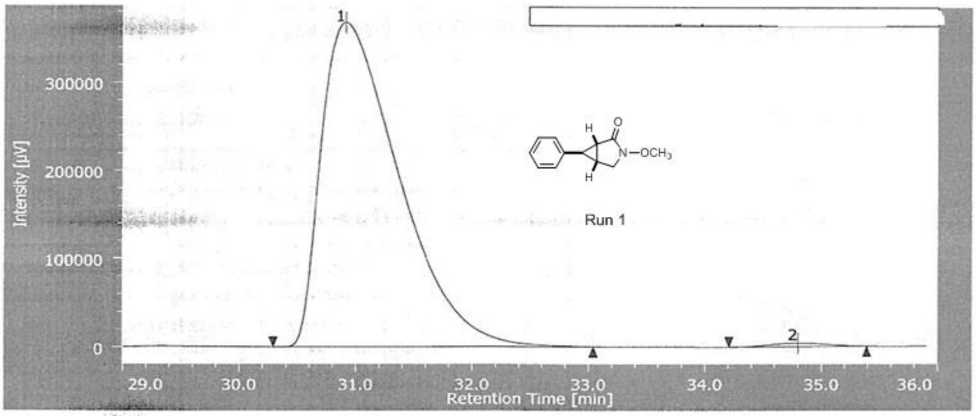

entry	tR[min]	area	area %
1	30.90	16956096	99.17
2	34.80	142997	0.83

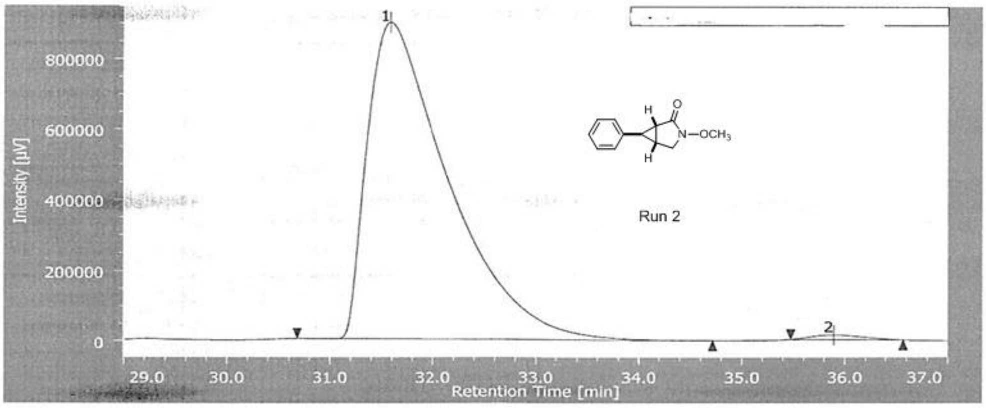

entry	tR[min]	area	area %
1	31.58	49236765	99.61
2	35.89	456987	0.39

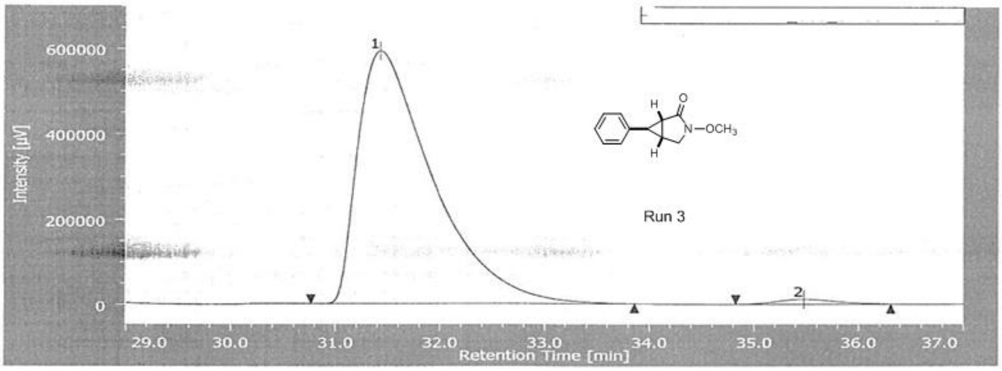

entry	tR[min]	area	area %
1	31.42	29461318	98.50
2	35.47	447790	1.50

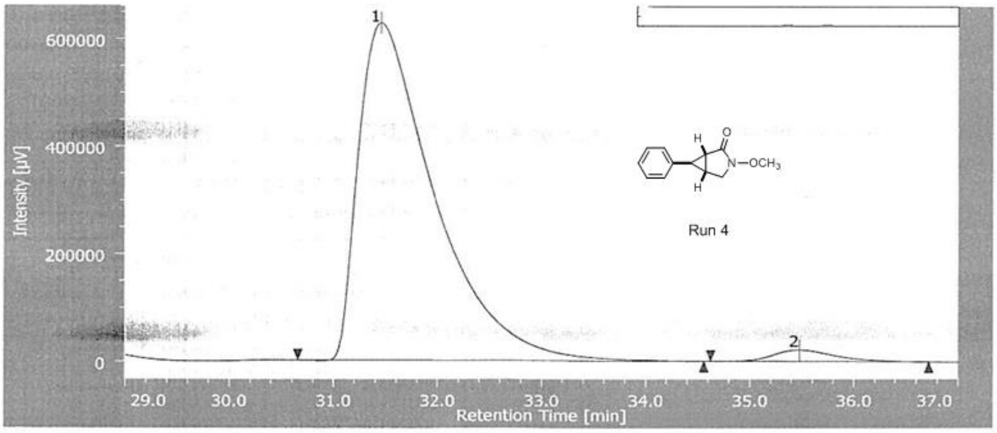

entry	tR[min]	area	area %
1	31.45	31761845	97.40
2	35.47	846789	2.60

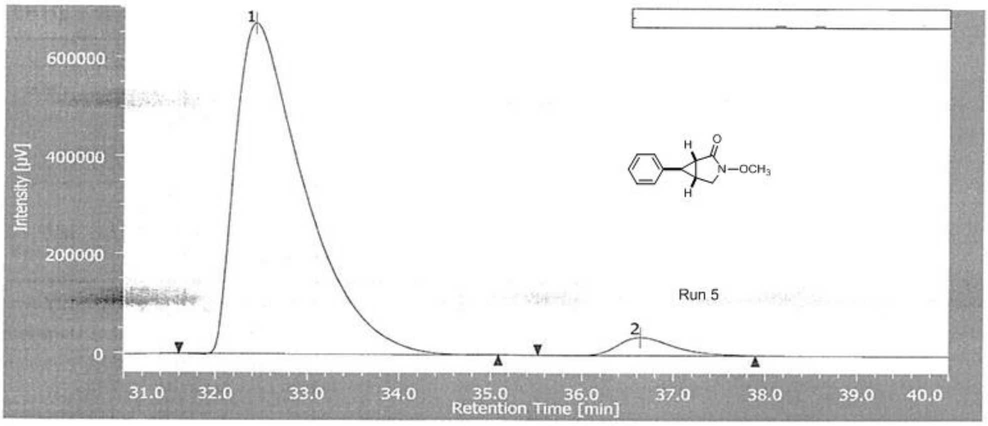

entry	tR[min]	area	area %
1	32.42	35202200	95.76
2	36.63	1557696	4.23

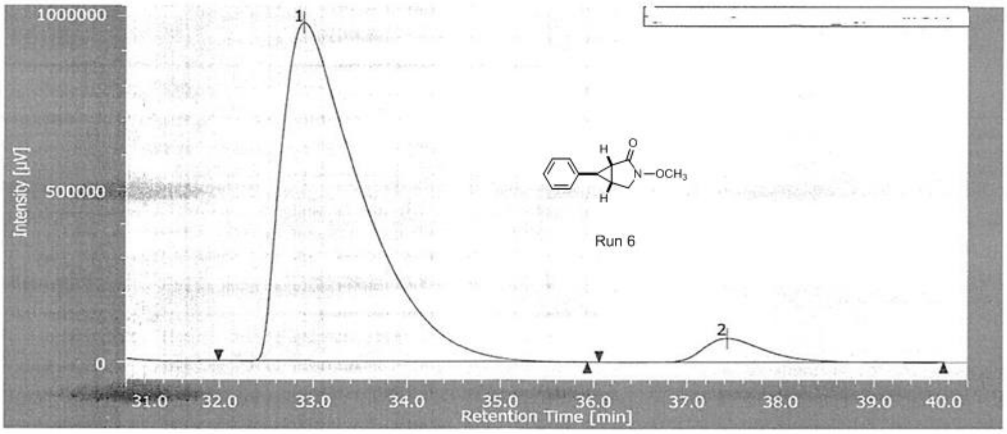

entry	tR[min]	area	area %
1	32.89	58005665	94.79
2	37.42	3191079	5.21

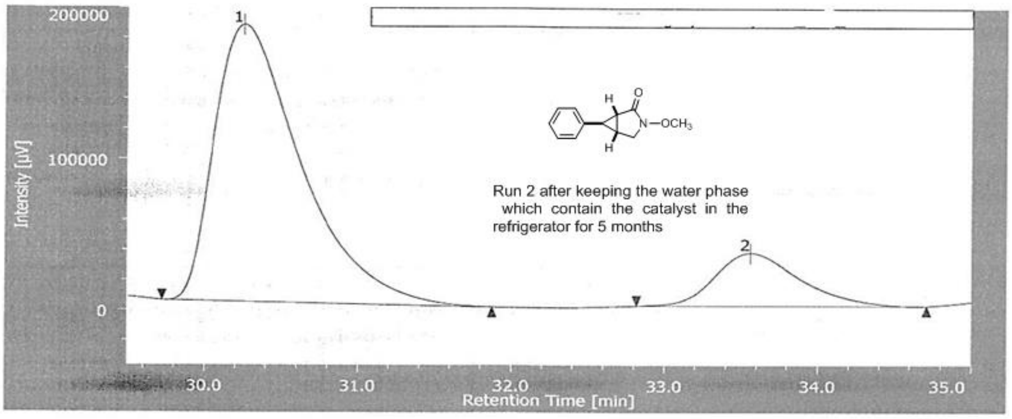

entry	tR[min]	area	area %
1	30.25	7184688	84.18
2	33.56	1349575	15.82

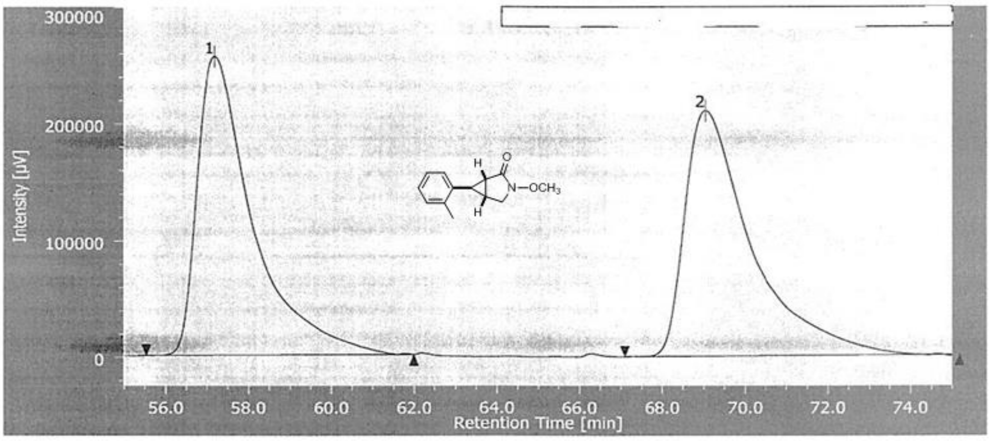

entry	tR[min]	area	area %
1	57.18	23931962	50.39
2	69.03	23559879	49.61

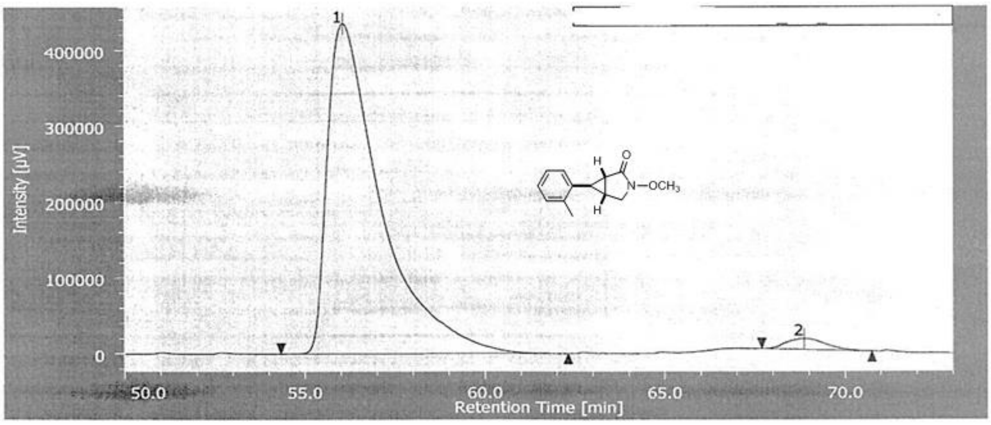

entry	tR[min]	area	area %
1	56.05	44528683	97.61
2	68.85	1089492	2.39

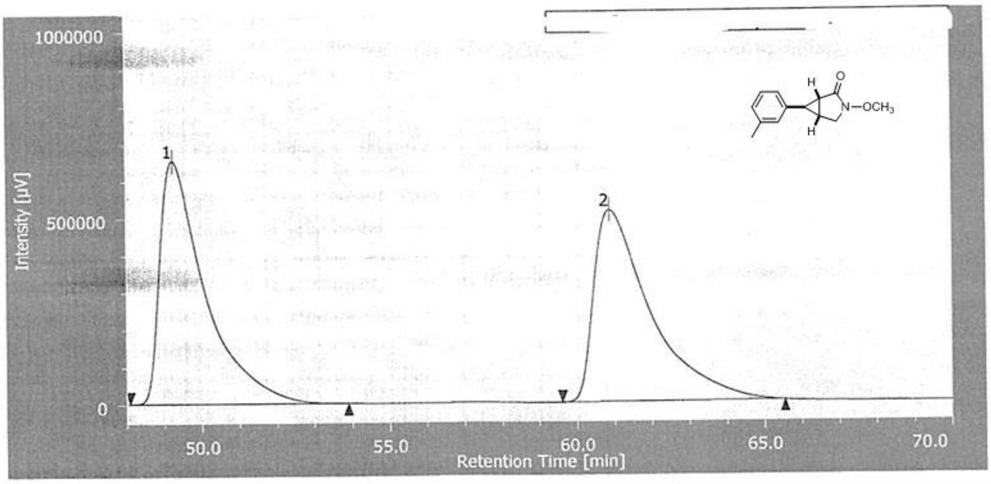

entry	tR[min]	area	area %
1	49.24	54674636	50.45
2	60.87	53699095	49.55

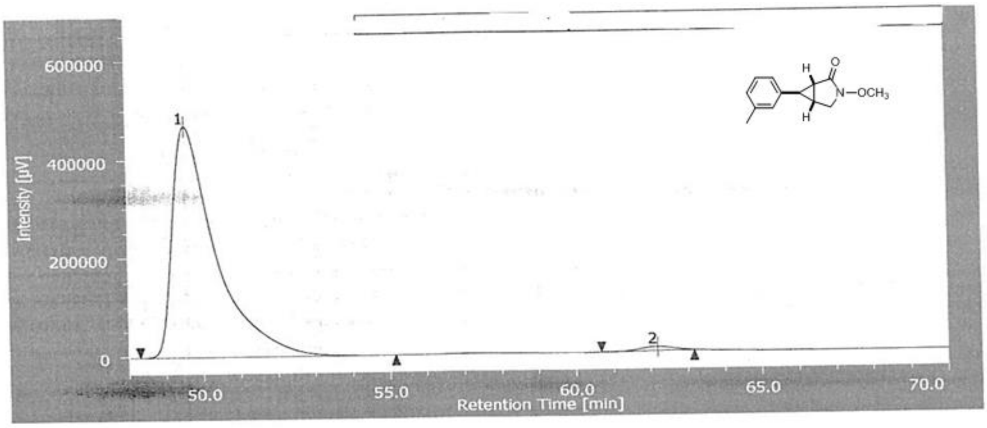

entry	tR[min]	area	area %
1	49.55	37895724	98.65
2	62.19	515166	1.34

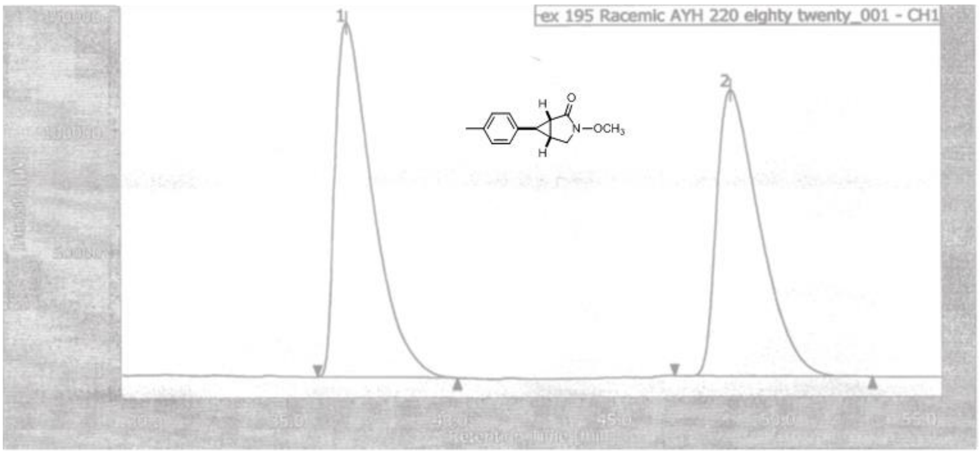

entry	tR[min]	area	area %
1	36.85	11709831	50.26
2	48.56	11584762	49.74

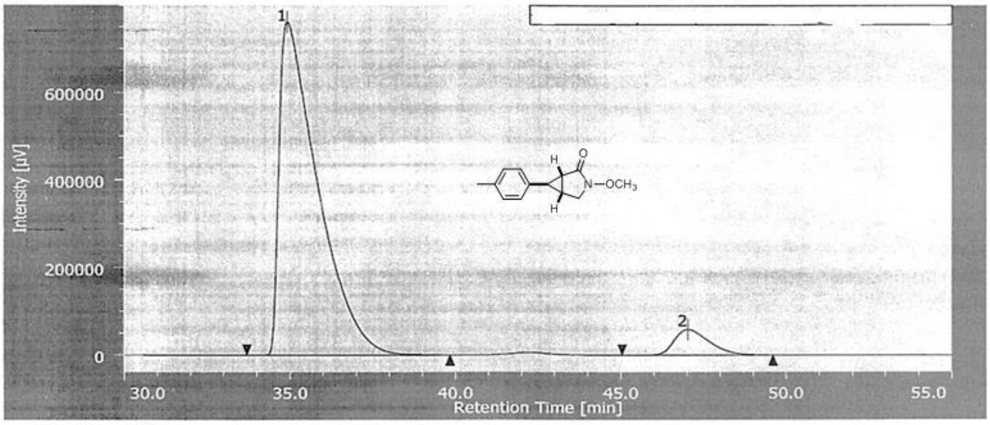

entry	tR[min]	area	area %
1	34.91	61559416	93.53
2	47.02	4254625	6.47

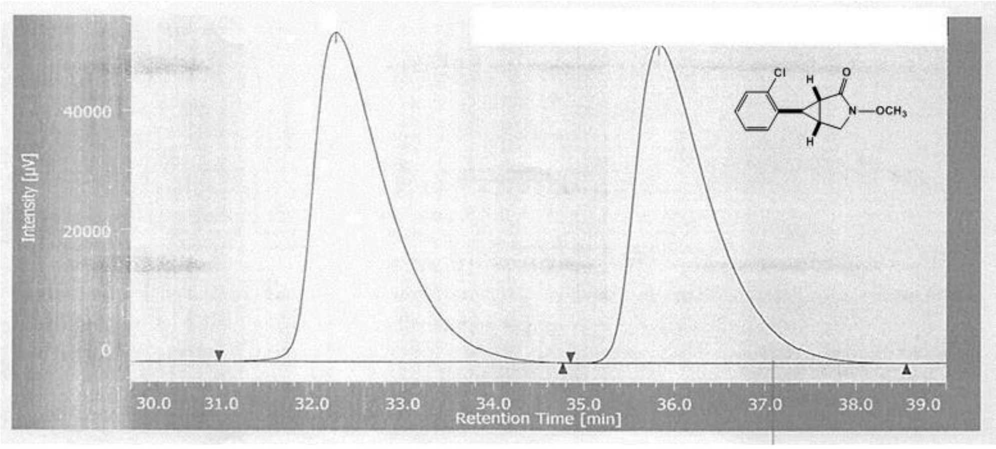

entry	tR[min]	area	area %
1	32.25	3138136	50.36
2	35.81	3093540	49.64

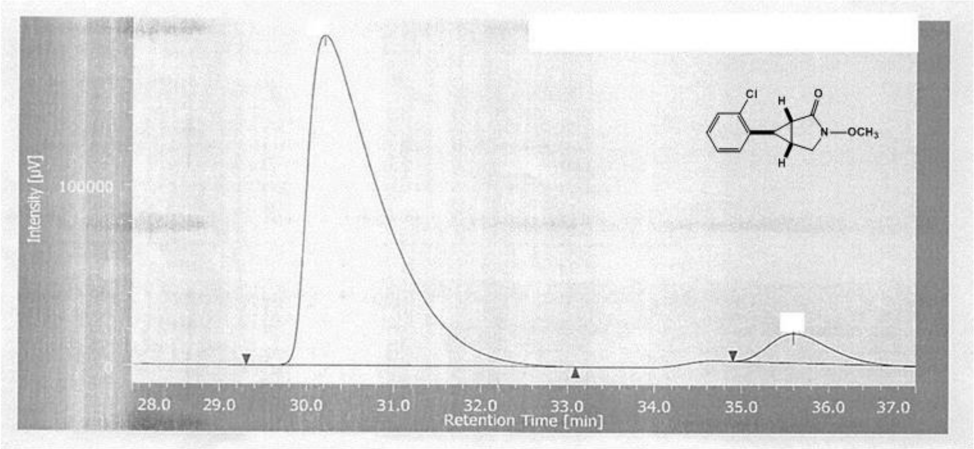

entry	tR[min]	area	area %
1	30.94	10590639	93.16
2	35.35	777500	6.83

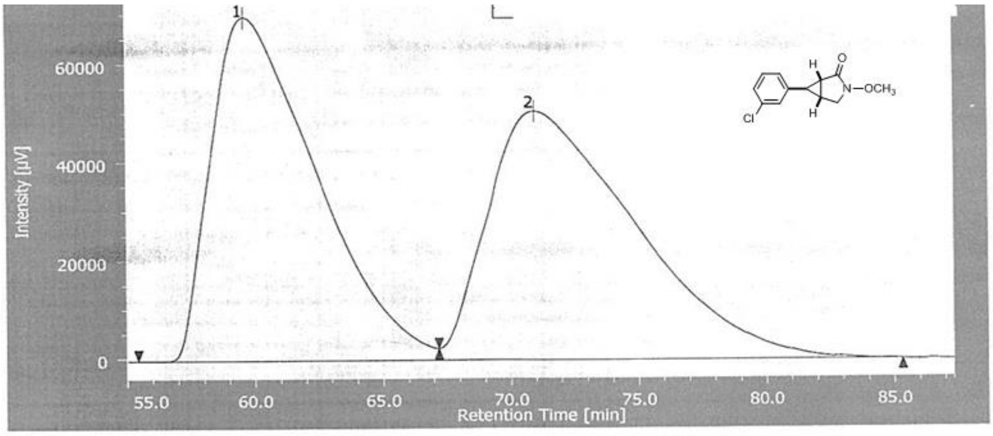

entry	tR[min]	area	area %
1	59.61	20018088	49.68
2	70.94	20273120	50.32

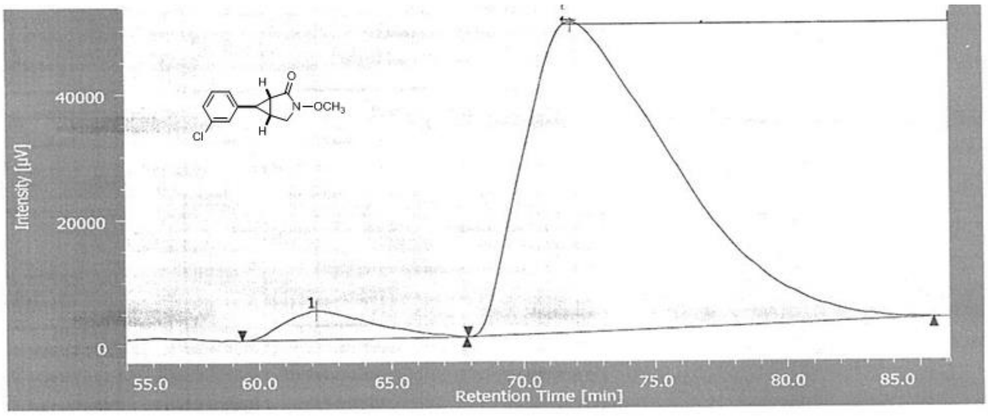

entry	tR[min]	area	area %
1	62.16	1071692	5.24
2	71.85	19381762	94.76

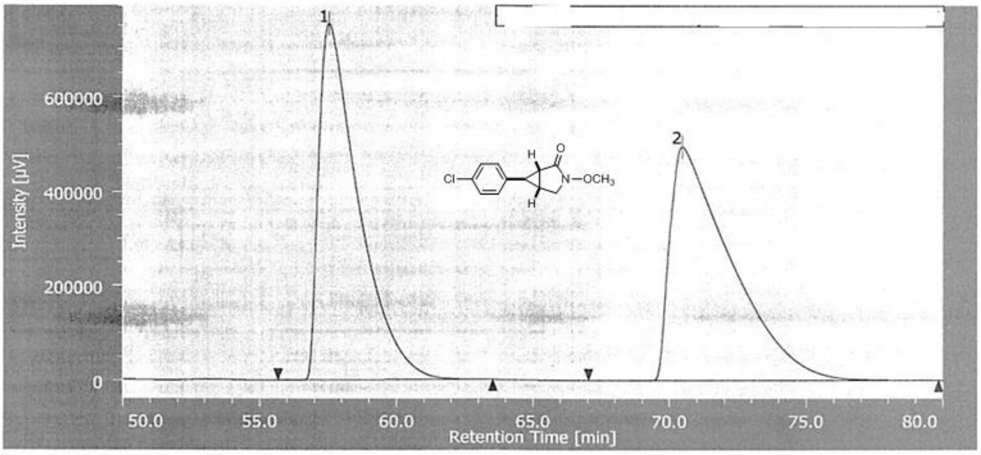

entry	tR[min]	area	area %
1	57.53	76070646	49.94
2	70.45	76242002	50.06

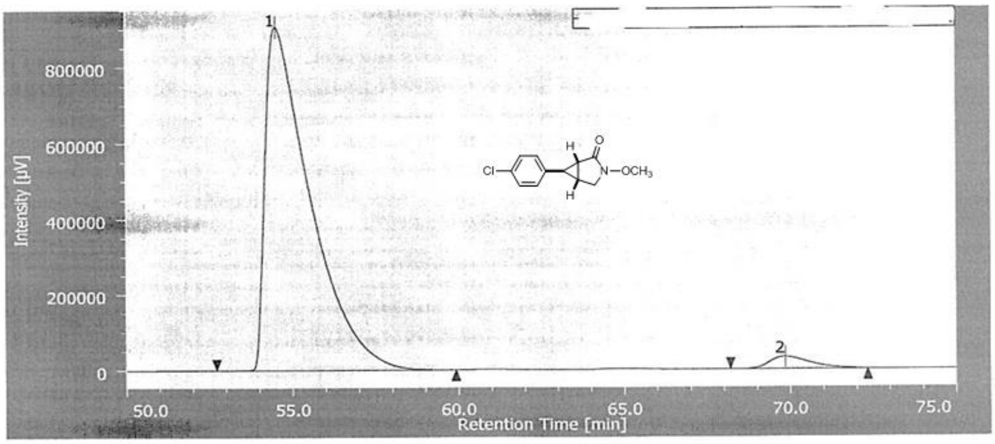

entry	tR[min]	area	area %
1	54.52	85496013	96.95
2	69.84	2686559	3.04

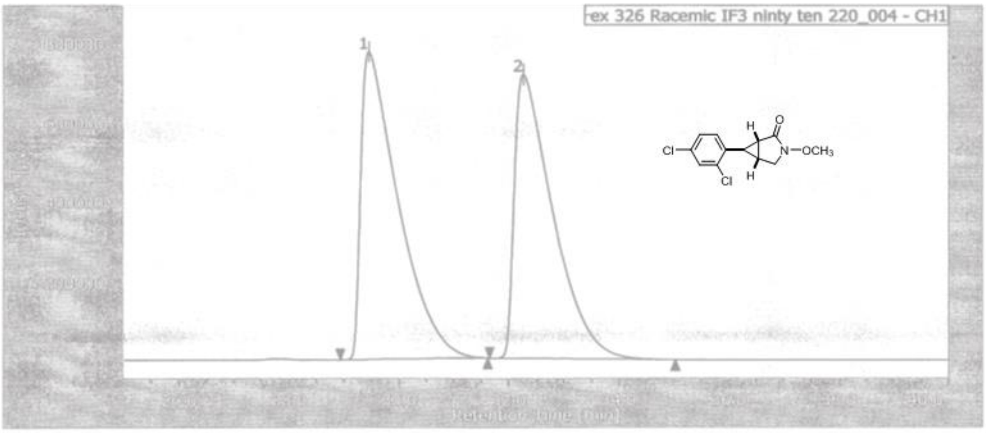

entry	tR[min]	area	area %
1	29.46	36252165	51.23
2	32.27	34515533	48.77

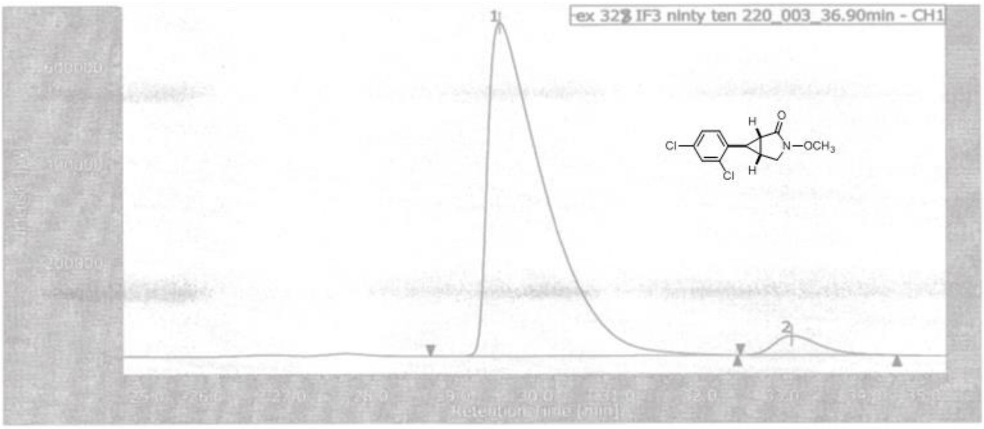

entry	tR[min]	area	area %
1	29.57	32849913	95.53
2	33.12	1535069	4.47

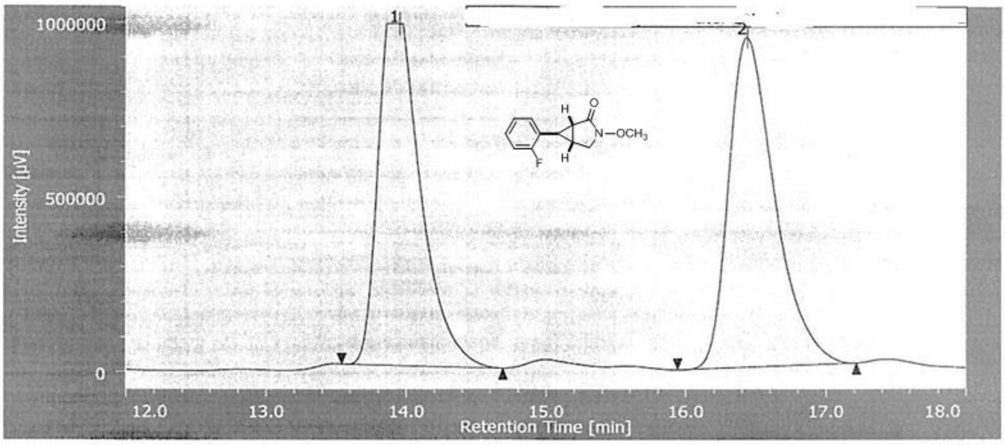

entry	tR[min]	area	area %
1	13.95	21116277	49.34
2	16.43	21679616	50.65

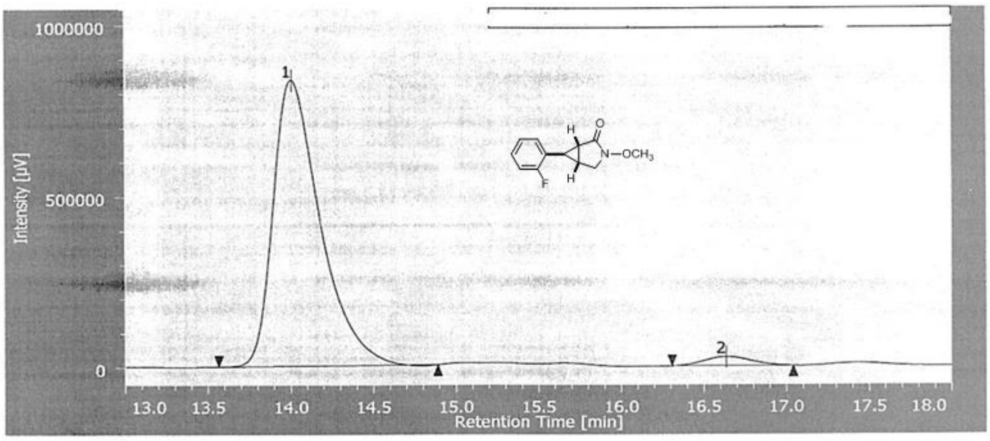

entry	tR[min]	area	area %
1	14.00	16537511	96.98
2	16.63	513845	3.02

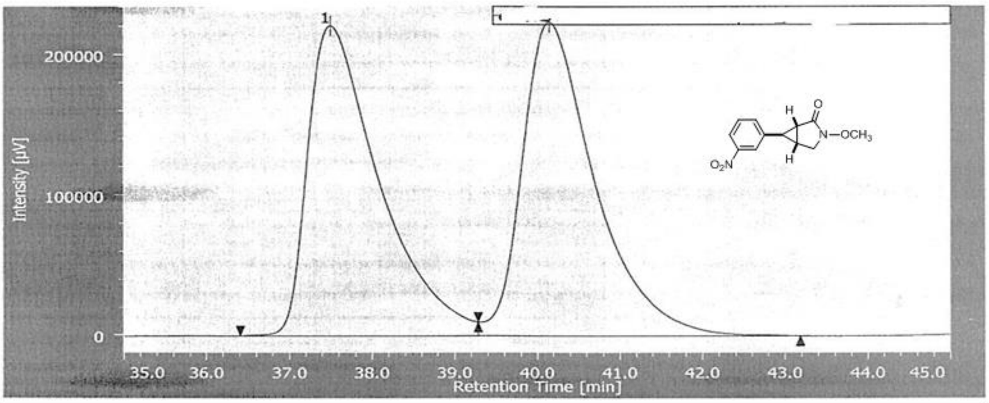

entry	tR[min]	area	area %
1	37.50	13316778	49.35
2	40.15	13669404	50.65

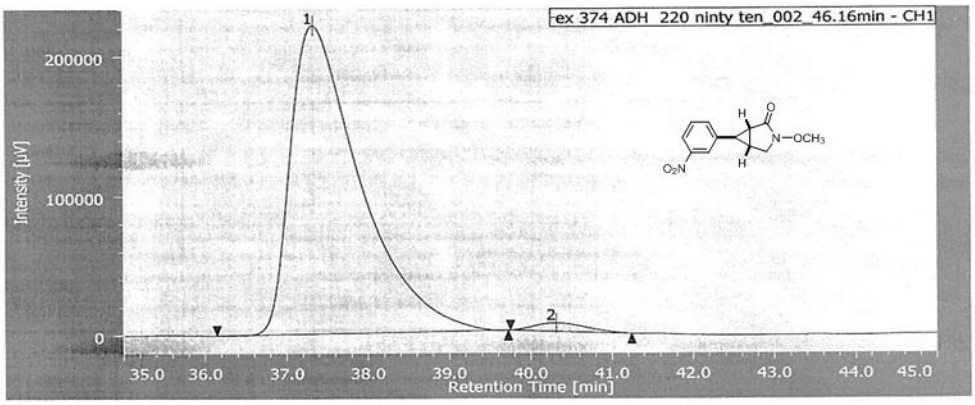

entry	tR[min]	area	area %
1	37.33	13151247	97.98
2	40.31	270389	2.02

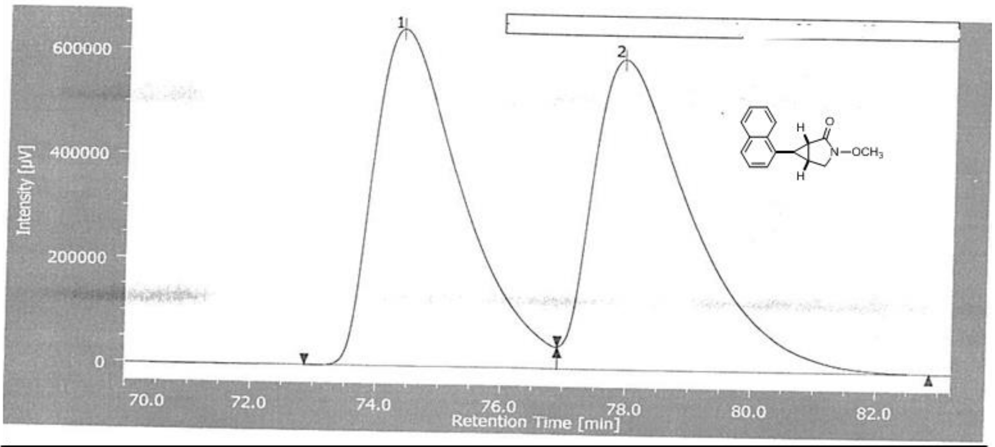

entry	tR[min]	area	area %
1	74.37	63405199	49.15
2	77.91	65598546	50.85

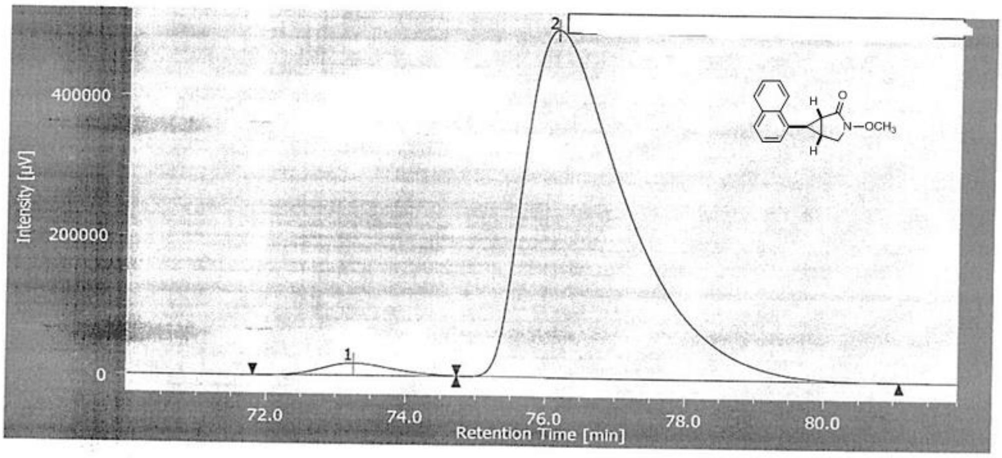

entry	tR[min]	area	area %
1	73.25	1243073	2.40
2	76.13	50644713	97.60

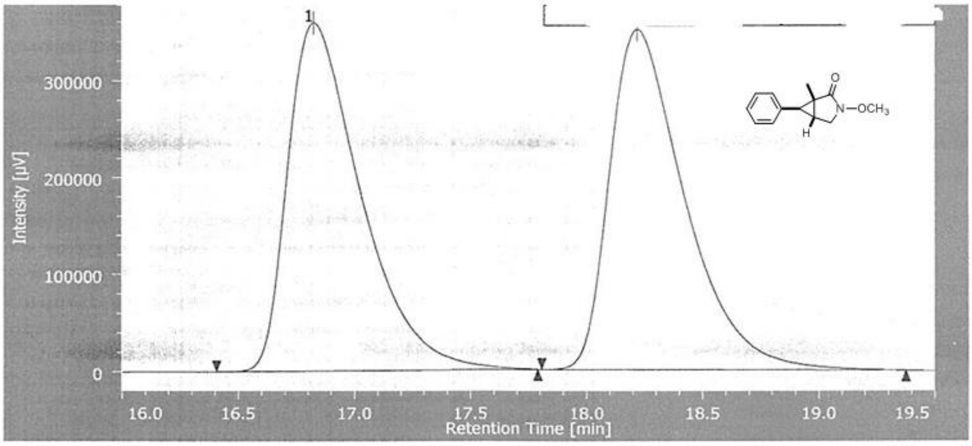

entry	tR[min]	area	area %
1	16.82	7849172	50.60
2	18.22	7885385	49.40

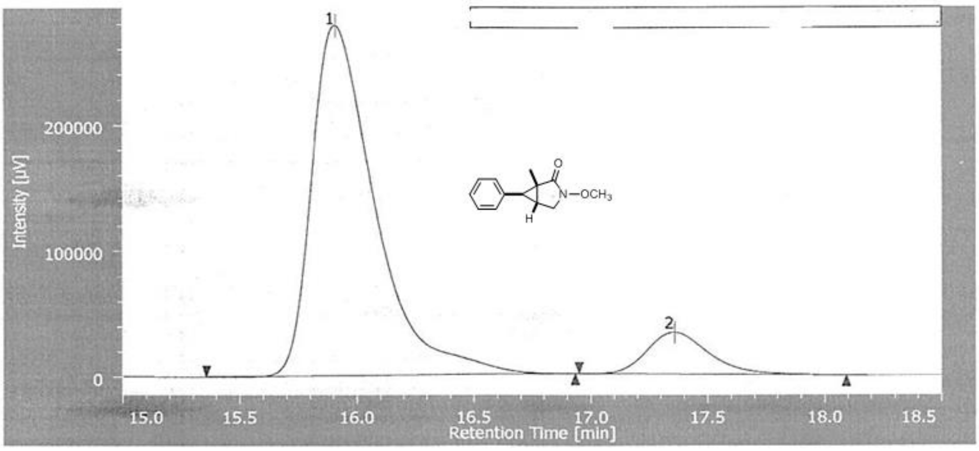

entry	tR[min]	area	area %
1	15.90	5221816	89.54
2	17.36	610287	10.46

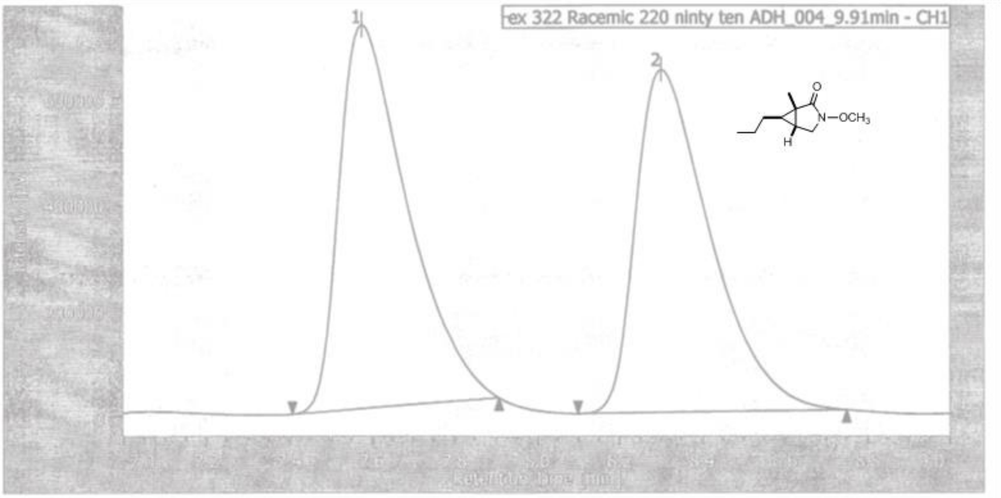

entry	tR[min]	area	area %
1	7.57	8296203	50.73
2	8.30	8058874	49.27

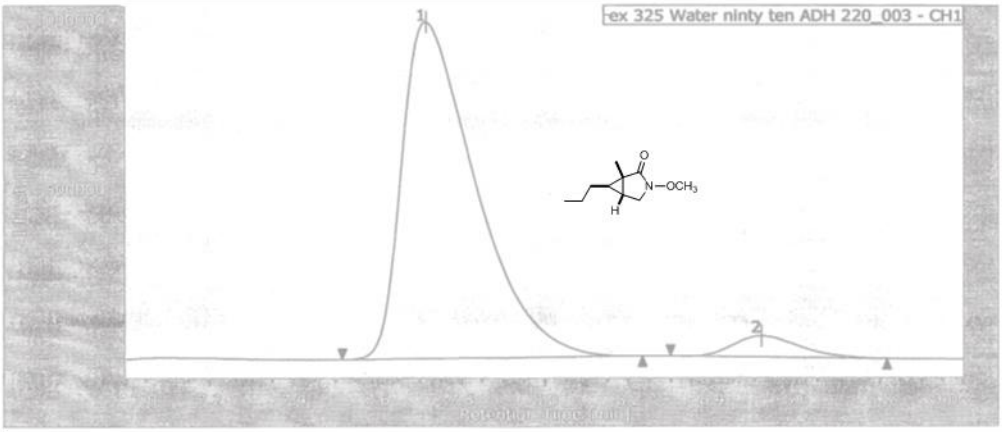

entry	tR[min]	area	area %
1	7.71	12038739	94.77
2	8.51	664794	5.23

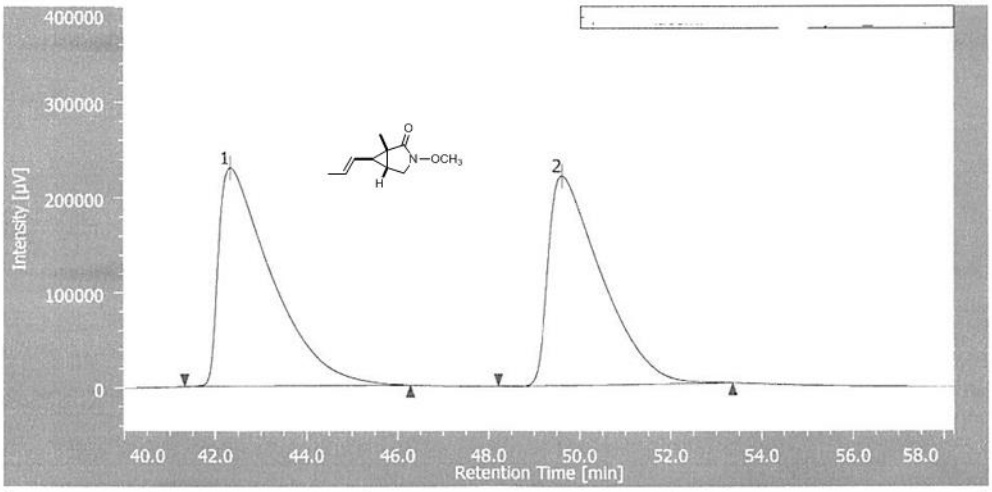

entry	tR[min]	area	area %
1	42.32	18562087	50.88
2	49.61	17921631	49.12

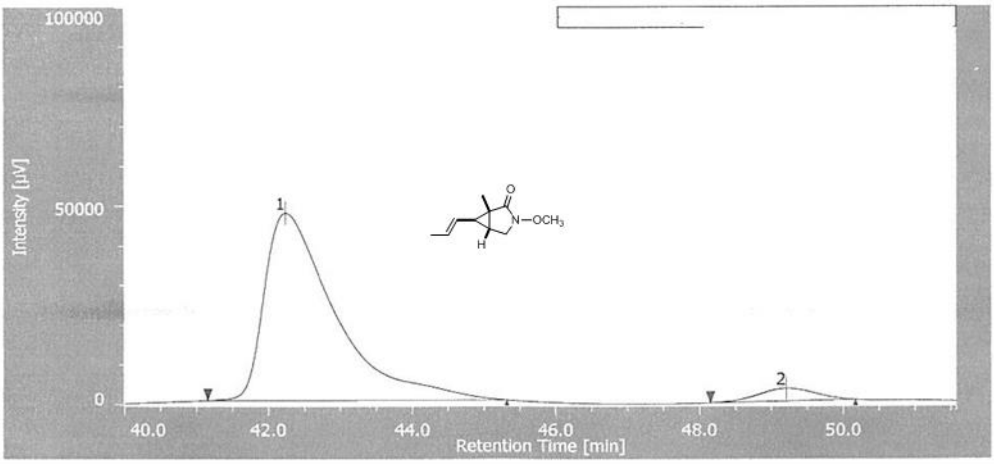

entry	tR[min]	area	area %
1	42.23	3234054	94.86
2	49.20	175230	5.14

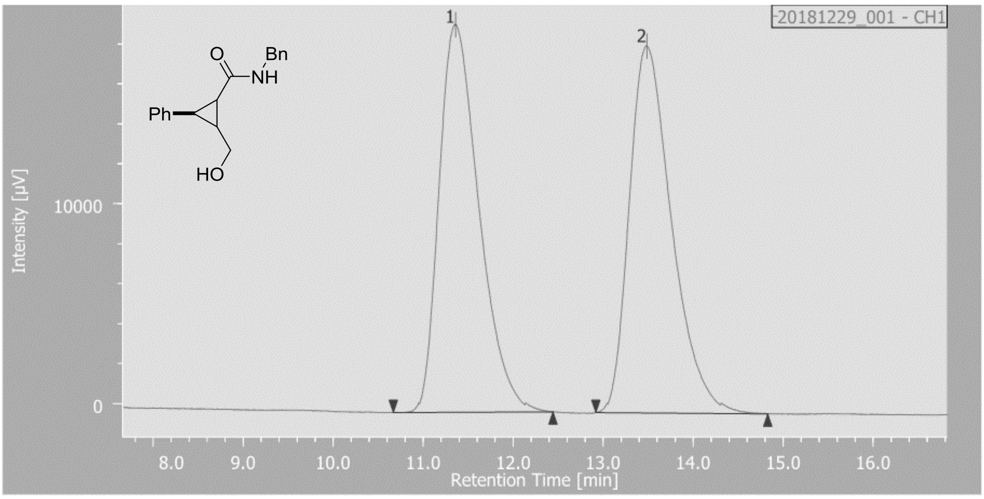

entry	tR [min]	area	area %
1	11.35	601642	50.06
2	13.48	600040	49.58

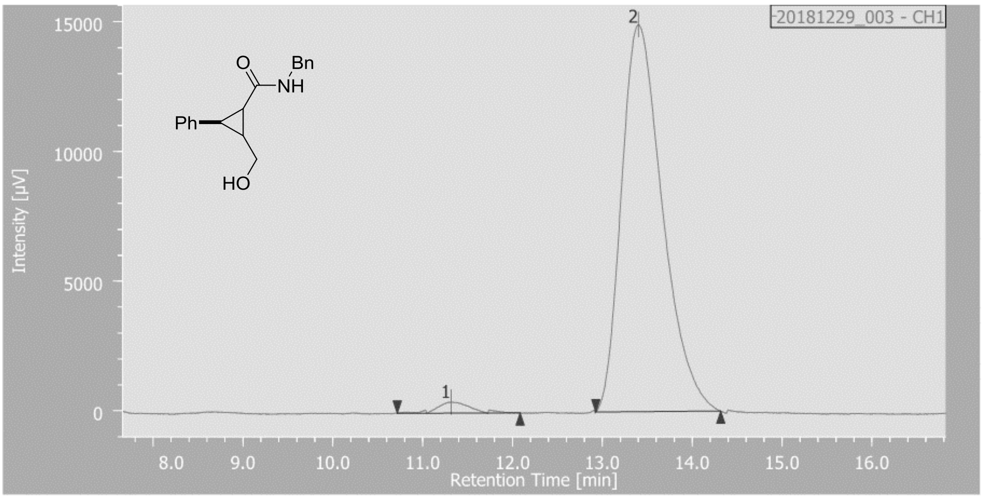

entry	tR [min]	area	area %
1	11.31	10074	2.08
2	13.40	474020	97.91

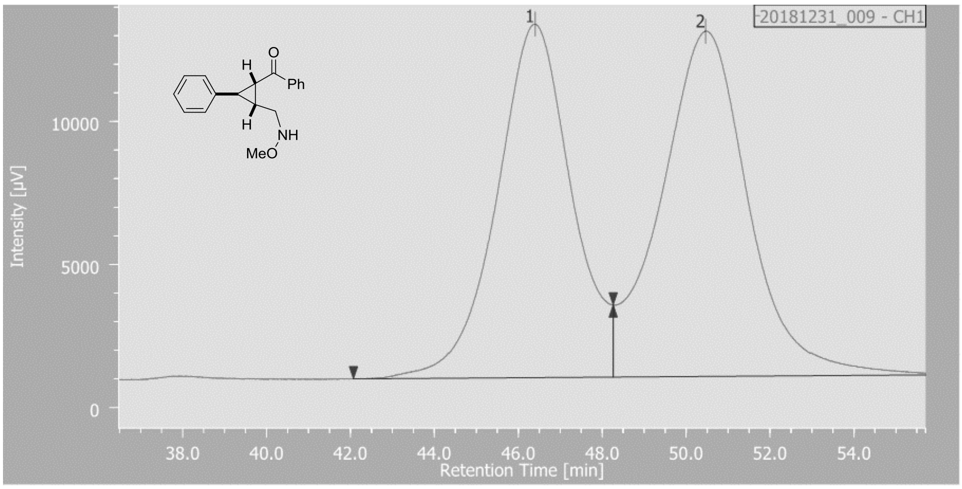

entry	tR [min]	area	area %
1	46.40	1527824	46.60
2	50.45	1750380	53.39

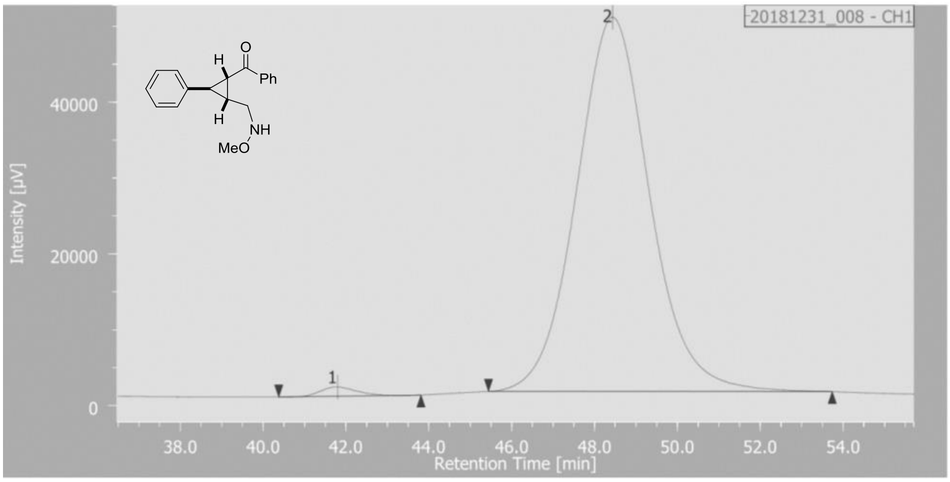

entry	tR [min]	area	area %
1	41.80	72701	1.18
2	48.43	6060994	98.85

12. References

[1] Bolsønes, M.; Bonge-Hansen, T. H.; Bonge-Hansen, T. *Synlett*, 2014, 25, 221–224.

[2] Abu-Elfotoh, A.-M.; Phomkeona, K.; Shibatomi, K.; Iwasa, S. *Angew. Chem. Int. Ed*. 2010, 49, 8439–8443.

[3] Abu−Elfotoh, A. –M.; Nguyen, D. P. T.; Chanthamath, S.; Phomkeona, K.; Shibatomi, K.; Iwasa, S., *Adv. Synth*. Catal. 2012, 354, 3435−3439.

[4] Nakagawa, Y.; Chanthamath, S.; Shibatomi, K.; Iwasa, S. *Org. Lett.* 2015, 17, 2792– 2795.

[5] Mandour, H. S. A.; Chanthamath, S.; Shibatomi, K.; Iwasa, S. *Adv. Synth. Catal*. 2017, 359, 1742– 1746.

[6] This part is partially taken from Dr. Mandour's Ph.D. theses (Toyohashi University of Technology, 2017).
